# The effect of non‐oral hormonal contraceptives on hypertension and blood pressure: A systematic review and meta‐analysis

**DOI:** 10.14814/phy2.15267

**Published:** 2022-05-04

**Authors:** Cindy Z. Kalenga, Sandra M. Dumanski, Amy Metcalfe, Magali Robert, Kara A. Nerenberg, Jennifer M. MacRae, Zahra Premji, Sofia B. Ahmed

**Affiliations:** ^1^ 157745 Cumming School of Medicine University of Calgary Calgary Alberta Canada; ^2^ Libin Cardiovascular Institute University of Calgary Calgary Alberta Canada; ^3^ Alberta Kidney Disease Network Calgary Alberta Canada; ^4^ 157744 Alberta Children's Hospital Research Institute Calgary Alberta Canada; ^5^ 157745 University of Victoria Victoria British Columbia Canada

## Abstract

Oral contraceptives (OC) are associated with increased risk of hypertension and elevated blood pressure (BP). Whether non‐oral hormonal contraceptives have similar associations is unknown. We sought to investigate the effect of non‐oral hormonal contraceptive (NOHC) use on the risk of hypertension and changes in BP, compared to non‐hormonal contraceptive and OC use. We searched bibliographic databases (MEDLINE, EMBASE, Cochrane Central Register of Controlled Trials) until August 2020. Studies reporting risk of hypertension or changes in systolic and diastolic BP with NOHC use compared with either non‐hormonal contraceptive or OC use. Abstract screening, full‐text review, data extraction, and quality assessment were completed in duplicate. For studies reporting dichotomous outcomes, we reported results as relative risk with 95% confidence intervals (CI). A random‐effects model was used to estimate pooled weighted mean difference and 95% CI of change in BP. Twenty‐five studies were included. A lower incidence of hypertension was observed with injectable contraceptive use compared to non‐hormonal contraceptive and OC use, although it was unclear if this was statistically significant. Compared to non‐hormonal contraceptive use, injectable contraceptive use was associated with increased BP (SBP: 3.24 mmHg, 95%CI 2.49 to 3.98 mmHg; DBP: 3.15 mmHg, 95%CI 0.09 to 6.20 mmHg), the hormonal intra‐uterine device use was associated with reduced BP (SBP: −4.50 mmHg, 95%CI −8.44 to −0.57 mmHg; DBP: −7.48 mmHg, 95% −14.90 to −0.05 mmHg), and the vaginal ring was associated with reduced diastolic BP (−3.90 mmHg, 95%CI −6.67 to −1.13 mmHg). Compared to OC use, the injectable contraceptive use was associated with increased diastolic BP (2.38 mmHg, 95%CI 0.39 to 4.38 mmHg). NOHC use is associated with changes in BP which differ by type and route of administration. Given the strong association between incremental increases in BP and cardiovascular risk, prospective studies are required.

## Graphical abstract

Oral contraceptives are associated with increased risk of hypertension and elevated blood pressure, though whether this also applies to non‐oral hormonal contraceptives is unknown. Our systematic review and meta‐analysis suggest that non‐oral hormonal contraceptive use is associated with changes in blood pressure, which differ by type and route of administration.

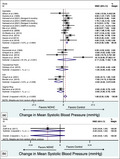

## INTRODUCTION

Cardiovascular disease is the leading cause of death in women worldwide (Garcia et al., 2016; Naghavi et al., 2017; Wenger et al., 2018; Wilmot et al., 2015). Despite overall improvements in cardiovascular mortality, the incidence and mortality of coronary heart disease in younger women (25–54 years old) are unchanged (Wilmot et al., 2015), highlighting the urgent need to investigate female‐specific cardiovascular risk factors in the premenopausal population.

Worldwide, an estimated 842 million women currently use contraception (United Nations, Department of Economic, & Social Affairs, Population Division, 2019). Oral contraceptive (OC) use is the most common hormonal contraceptive method, with 151 million reported users globally (United Nations, Department of Economic, & Social Affairs, Population Division, 2019). OC use is associated with an increased risk of hypertension and myocardial infarction (Bhupathiraju et al., 2016; Lewis et al., 1997; Rosenberg et al., 1980; Tanis et al., 2001). Elevated blood pressure (BP) contributes to cardiovascular morbidity and mortality (Lim et al., 2012) and OC use is associated with increases in BP (Ahmed et al., 2004; Davis et al., 2017), in a dose‐dependent manner (Meade et al., 1977). Although the exact mechanism is unclear, it is postulated that oral estrogen‐containing contraceptives undergo first‐pass hepatic metabolism, leading to upregulation of renin‐angiotensin‐aldosterone system (RAAS) activity (Ahmed et al., 2004; Deschepper, 1994; Goldhaber et al., 1984; Kang et al., 2001; Pond & Tozer, 1984), resulting in vasoconstriction, sodium reabsorption and ultimately increased BP (Fountain & Lappin, 2020).

Non‐oral hormonal contraceptives (NOHC) delivery systems avoid first‐pass hepatic metabolism and, in contrast to OC use, are not associated with increased RAAS activity (Odutayo et al., 2015). Systemic non‐oral hormonal contraception, such as the hormonal injectable, implant, and transdermal patch release estrogen and/or progestin throughout the body. In contrast, non‐systemic NOHC, including the hormonal intrauterine device (IUD) and vaginal ring releases hormones locally into the reproductive system. However, whether NOHC use is associated with an increased risk of hypertension or changes in blood pressure is unclear. Our study aimed to investigate the effect of non‐oral hormonal contraceptive, compared to non‐hormonal contraceptive (controls) and oral contraceptive use on the risk of hypertension and blood pressure.

## 1 MATERIALS AND METHODS

### 1.1 Protocol

The systematic review protocol is being reported according to the Preferred Reporting Items for Systematic Review and Meta‐analysis Protocols (PRISMA‐P) guidlines (Moher et al., 2015) and was registered in PROSPERO (CRD42018117258),

### 1.2 Information sources and literature search

Electronic searches of MEDLINE (via Ovid, 1946—August 13, 2020), EMBASE (via Ovid, 1974—August 13, 2020), and Cochrane Central Register of Controlled Trials (CENTRAL) (via Ovid, 1991—August 13, 2020) without language restrictions and limited to human subjects was conducted. The first portion of the search strategy on the exposure (Supplementary data Table S1) included MeSH subject headings and search terms for contraceptives and their formulations (i.e., “Estrogen”, “Estradiol”, “Contraceptives”, “Medroxyprogesterone Acetate”, “Levonorgestrel”, “Etonogesterel”, “Birth Control”, etc.) were combined using the “OR” operator and linked with the term “Non‐Oral” using the “AND” operator. In addition, MeSH subject headings and search terms for specific types of non‐oral hormonal contraceptives were included in the search strategy (i.e., “Mirena IUD”, “Levonorgestrel Release Intrauterine Device/System”, “Evra”, “DMPA Injectable”, “Nuva‐Ring”, “Implanon”, etc.) and linked to non‐oral hormonal contraceptive terms using the “OR” operator. The second portion of the search included terms related to the outcome variable. Specifically, MeSH subject headings and search terms for blood pressure and hypertension were linked using the “OR” operator. Finally, the linked exposure terms were combined with the linked outcome terms using the “AND” operator.

The literature search was drafted, and peer‐reviewed by a librarian using the Peer Review of Electronic Search Strategies checklist (Avila et al., 1996). The search was supplemented with a grey literature search using various methods, including scanning the reference lists of included studies, dissertations, conference papers, and ongoing research, and asking experts in the field. Study authors were contacted for missing data.

### 1.3 Study selection and data extraction

Titles and abstracts were assessed for eligibility for full‐text review by two independent reviewers (C.Z.K and S.M.D.). All conflicts were resolved through discussion with a third reviewer (S.B.A.). The same process was followed for screening potentially relevant full‐text articles. Data extraction was conducted in duplicate and the data spreadsheet included the following items: study identifiers (title, author, year of publication, location, setting), study characteristics (design, sample size, inclusion/exclusion criteria, follow‐up period), population characteristics (age, smoking status, BMI, ethnicity, baseline blood pressure), exposure characteristics (type and dose of NOHC), control characteristics (non‐hormonal contraceptive type and and type and dose of OC) and outcome characteristics (hypertension, systolic and diastolic blood pressure). The purpose of this study was to evaluate the effect of different forms of hormonal contraceptives on hypertension and blood pressure. As such, the control group were non‐hormonal contraceptive users rather than no use of contraceptives. The inclusion criteria were as follows: (1) study population was premenopausal women; (2) intervention was NOHC use; (3) comparator was either non‐hormonal contraceptive use (e.g., barrier methods, copper IUD, coitus interruption, etc.) or OC use; (4) outcome was hypertension or systolic blood pressure (SBP) and diastolic blood pressure (DBP); and (5) study design was observational study or randomized controlled trial (RCT).

### 1.4 Appraisal of methodological quality and risk of bias

Each included article was independently assessed for quality and risk of bias, using the Newcastle‐Ottawa Scale (Wells et al.,) for observational studies and the Cochrane Risk of Bias tool for RCTs (Sterne et al., 2019 ).

### 1.5 Data synthesis and analysis

For studies reporting dichotomous outcomes (i.e., a diagnosis of hypertension), results were reported as relative risk with 95% Confidence Interval (CI). Studies reporting continuous outcomes (SBP, DBP) with the average effect estimate and the associated measure of variance (i.e., standard deviation) were included in the analysis, with standard errors imputed when feasible. Summary estimates of the weighted mean differences in SBP and DBP were obtained using a random‐effects model, stratified by type of NOHC use. Methodological and statistical heterogeneity was evaluated using the I^2^ statistic (Goodman et al., 2011) and Cochrane’s Q tests of heterogeneity (Goodman et al., 2011). A two‐sided *p* < 0.05 was considered statistically significant for all analyses. All analyses were performed using Stata (version STATA/SE 16.1; Stata Corp LP, College Station, TX). Effect estimates were reported as relative risk (RR) for dichotomous outcomes and mean differences for continuous outcomes.

## 2 RESULTS

### 2.1 Literature search

**FIGURE 1:**
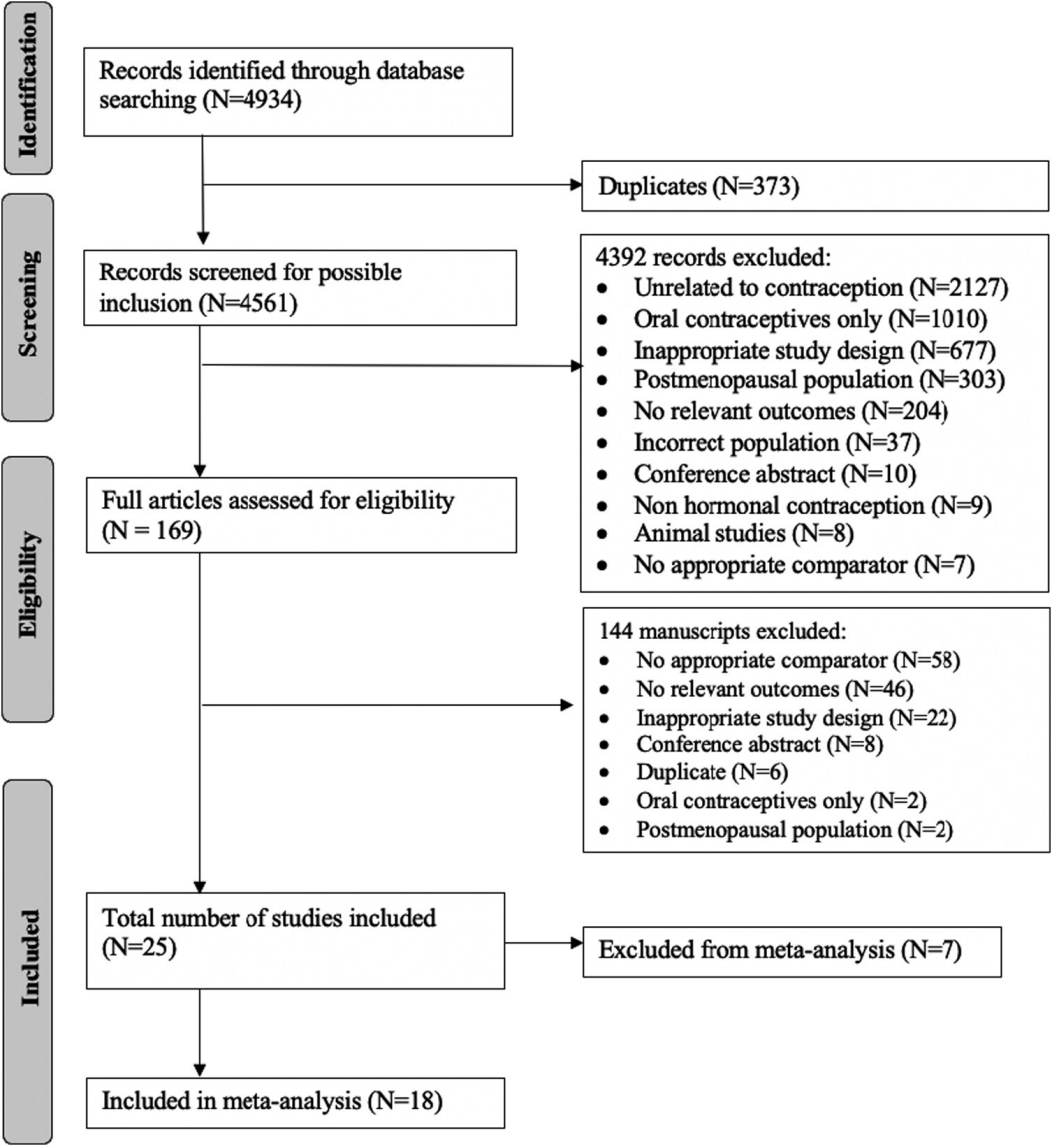
Prisma‐P flow diagram

The study selection flow is demonstrated in Figure 1. The literature search identified 4561 unique citations, of which 4392 were excluded after the title and abstract screening. Full‐text review of 169 citations identified 25 studies (12 prospective cohort studies, 10 cross‐sectional studies, and 3 RCTs) enrolling 7306 participants. Seven were excluded from the meta‐analyses due to lack of reporting of mean SBP and DBP; these studies reported either the change in BP, interquartile range or omitted the measure of variance. Agreement between the two independent reviewers was 92% at the title/abstract screening stage and 88% at the full‐text review stage.

### 2.2 Study characteristics

#### 2.2.1 Observational studies

In the 22 observational studies (Al‐Obaidy & Al‐Youzbaki, 2014; Asare et al., 2014; Avila et al., 1996; Barreiros et al., 2010; Bender et al., 2013; Cursino et al., 2018; Hameed et al., 2001; Haroon & Naveed, 2014; Kurunmaki, 1983; Lizarelli et al., 2009; Mia et al., 2004; Morin‐Papunen et al., 2008; Odutayo et al., 2015; Ortayli et al., 2001; Oyelola, 1993; Shen et al., 1994; Sivin et al., 1981; Taneepanichskul et al., 1999; Wilson et al., 1984; Xiang et al., 2007; Yasmin et al., 1993; Yildirim et al., 1997) (12 studies including controls, 2 including OC users, and 8 studies including both comparators), a total of 6934 patients were included with the mean age ranging from 22.9 to 42.2 years. Study publication dates ranged from 1983 to 2015 with the majority of studies published after 2000. There was notable heterogeneity in the study setting including 12 different countries. Prospective cohort studies ranged from 3 months to 6 years in duration, with 70% of studies following participants for at least 1 year. Two studies reported on the development of hypertension with NOHC use, while 14 studies reported the mean SBP and DBP with NOHC use and were included in the meta‐analyses. Five routes of administration of NOHC were included: Injectable (13 studies, *n* = 5142), implant (6 studies, *n* = 1212), transdermal patch (1 study, *n* = 35), hormonal IUD (3 studies, *n* = 979), and vaginal ring (4 studies, *n* = 857). Additional study characteristics are outlined in Table 1.

**TABLE 1:** Summary of study characteristics

Authors (year)	Country	Study population	Exposure group				Comparator group				
			N	Mean Age	Route	Formulation	N	Mean age	Route	Formulation	Study length (months)
Prospective cohort studies											
Kurunmaki (1983)	Finland	Healthy women; admitted to clinic for termination of pregnancy	38	26.4	Implant	LNG	38	26.4	NHC	NR	12
Yildirim et al. (1997)	Turkey	Multiparous women with no hypertension; age 19–40; no contraindications to use of hormonal contraceptives; weight 45–80 kg	51	30.5	Implant	LNG	68	30.8	OC	Ethinyl estradiol +norgestrel	12
Shen et al. (1994)	China	Women with no hypertension or diabetes; regular menstrual cycles; no recent hormonal contraceptive use; on no drugs known to affect BP or interact with hormonal contraceptives	267	30.8	Implant	LNG	259	29.8	NHC	Stainless Steel IUD	11
Ortayli et al. (2001)	Turkey	Women with recent termination of pregnancy; no systemic diseases, genital tract problems, or uterine fibroids	50 50	27.9 28.8	Implant IUD	LNG	50	33.7	NHC	Withdrawal, Abstinence	12
Bender et al. (2013)	USA	Healthy women with intention to change their lifestyle; BMI >30; no recent hormonal contraceptive use; no history of PCOS, DM, HTN, CVD, dyslipidemia or liver disease	8 9	29.5 29.2	Implant IUD	Etonogestrel LNG	8	28.5	NHC	Copper IUD	6
Wilson et al. (1984)	Scotland	Women that attended family planning clinic; used no OC use in last 6 months; BP <140/90	20	NR	Injectable	Norethisterone oenanthate	20	NR	NHC	NR	12
Avila et al. (1996)	Brazil	Women with heart disease who received contraception	27	24.6	Injectable	MPA	35	24.4	OC	EE and gestodene	24
Hameed et al. (2001)	Pakistan	Healthy, married, multiparous women; age 25–40; on no contraception	40	NR	Injectable	DMPA and Norigest	40	NR	NHC	NR	3, 6
Xiang et al. (2007)	USA	Postpartum women with prior gestational DM; no chronic vascular disease, HTN, DM	94	29.8	Injectable	DMPA	448 429	31.3 28.9	NHC OC	NHC: NR OC: EE + norenthindrone/LNG	72
Cursino et al. (2018)	Brazil	Women with BMI <30; age 18–40; not previously on DMPA; no DM, HTN, PCOS, or other systemic diseases; on no drugs known to interact with hormonal contraceptives	15	28.7	Injectable	DMPA	15	28.3	NHC	Copper IUD	12
Sivin et al. (1981)	Various	Healthy women; age 18–35; no contraindications to use of hormonal contraceptive	1103	NR	Vaginal Ring	LNG and estradiol	1103	NR	NHC	NR	12
Barreiros et al. (2010)	Brazil	Women who attended pregnancy prevention session; no recent hormonal contraceptive use; no contraindications to estrogen	75	24.4	Vaginal Ring	Etonorgestrol and EE	75	24.4	NHC	NR	12
Cross sectional studies											
Asare et al. (2014)	Ghana	Women; age 20–49; no history of CVD or predisposing conditions	5 47	32.2 29.9	Implant Injectable	Etonogestrel DMPA	24 19	29.2 33.1	NHC OC	NHC: NR OC: Norinthindrone or EE +LNG	N/A
Oyelola (1993)	Nigeria	Women of low socioeconomic status; no FHx of CVD	16	38.4	Injectable	DMPA	18 18	35 36	NHC OC	NHC: NR OC: EE +norgestrel	N/A
Yasmin et al. (1993)	Bangladesh	Women of low socioeconomic status; age 20–40; no HTN, DM, liver disease, heart disease	15	NR	Injectable	Noristerat	60 32	NR	NHC OC	NHC: NR OC: EE +norgestrel	N/A
Taneepanichskul et al. (1999)	Thailand	Women using study contraceptives for 5+ years; age 37–50; no chronic disease (except HTN); no smoking or alcohol use	50	42.7	Injectable	DMPA	50	43.2	NHC	Copper IUD	N/A
Mia et al. (2004)	Bangladesh	Healthy non‐obese women using study contraceptives for 3–5 years; age 20–35; no DM or heart disease; no smoking	140	NR	Injectable	DMPA	60	NR	NHC	Abstinence, Copper IUD, Barrier, Timing	N/A
Lizarelli et al. (2009)	Brazil	Women with regular menstrual cycles using study contraceptives for 1+ year; age 18–30; no systemic disease or obesity; no smoking, alcohol or drug use	25	22.9	Injectable	DMPA	50 25	23.4 23.7	NHC OC	NHC: NR OC: EE +LNG	N/A
Al‐Obaidy et al. (2014)	Iraq	Healthy married women using study contraceptives; age 19–40; regular menstrual cycles; BMI <30; no DM, HTN, liver disease, hematologic disease; no smoking or alcohol use	38	30.9	Injectable	DMPA	44	30.1	NHC	NR	N/A
Haroon et al. (2014)	Pakistan	Women attending family planning clinics using study contraceptives	30	31	Injectable	DMPA or norethisterone oenanthate	30 30	32.1 29.9	NHC OC	NHC: NR OC: EE +norgestrel	N/A
Odutayo et al. (2015)	Canada	Healthy women using study contraceptives, age <40; no DM, HTN, CVD, kidney disease; no smoking	10	30	Transdermal patch	Norelgestromin	10	24	NHC OC	NHC: NR OC: EE +LNG	N/A
Morin‐Papunen et al. (2008)	Finland	Women on no DM medications	168	31	IUD	LNG	1959 687	31 31	NHC OC	NHC: Metal IUD, Barrier, Abstinence, Other OC: Various	N/A
Randomized controlled trials											
Battaglia et al. (2010)	Italy	Women with PCOS and BMI >30; age >18; no recent hormonal contraceptive use; no smoking or regular exercise; no DM, kidney or liver disease; no other gynecologic abnormalities	18	24.4	Vaginal Ring	EE +etonogestrel	19	23.4	OC	OC: EE +drospirenone	6
Mohamed et al. (2011)	Egypt	Women with regular menstrual cycles; age 17–42; no contraindications to use of hormonal contraceptives, recent hormonal contraceptive use or gynecologic abnormalities.	239	29.7	Vaginal Ring	EE + etonogestrel	245	30.9	OC	OC: EE +dropirenone	12
Zueff et al. (2017)	Brazil	Women with BMI 30–40; age 18–40; no contraindications to hormonal contraceptive use;no recent hormonal contraceptive use; no smoking, alcohol or drug use	50	31.2	IUD	LNG	40	31.8	NHC	Copper IUD, Barrier	12

Abbreviations: BMI, body mass index;.CEE, conjugated equine estrogen; CVD, cardiovascular disease; DBP, diastolic blood pressure; DM, diabetes mellitus; DMPA, depot medroxyprogesterone acetate; EE, ethinyl estradiol; HTN, hypertension; Hx, history; IUD, intrauterine device; LNG, levonorgestrel; NR, not reported; OC, oral contraceptive; PCOS, polycystic syndrome; SBP, systolic blood pressure.

#### 2.2.2 RCTs

In the 3 RCTs (Battaglia et al., 2010; Mohamed et al., 2011; Zueff et al., 2017) (1 study including controls, 2 including OC users), a total of 372 participants were included with the mean age ranging from 23.4 to 31.8 years. Publication dates ranged from 2010 to 2017 with significant heterogeneity in the study setting with studies being conducted in three different countries. One study included women using the hormonal IUD (*n* = 90) and two studies included women using the vaginal ring (*n* = 282). All three studies reported the mean SBP and DBP and were included in the meta‐analysis. Additional details regarding study characteristics are outlined in Table 1.

### 2.3 Study quality

Quality assessment of the observational studies demonstrated that most studies were of moderate or low quality (average score of 5.04 points on the New Castle Ottawa Scale, Supplementary Data Tables S2 and S3). Consistent quality indicators were cohort selection, assessment of outcomes, and length of follow‐up. However, less than half of the prospective cohort studies reported subjects lost to follow‐up. The risk of bias of the three RCTs was variable (Supplementary Data Table S4). Only one of three studies fully addressed its randomization and allocation processes, while two studies had a low risk for attrition and reporting bias.

### 2.4 Outcomes

#### 2.4.1 Hypertension

##### NOHC use compared to non‐hormonal contraceptive use

Of the 22 observational studies, hypertension incidence was reported in only one study although the criteria for hypertension were not defined. There were five participants with hypertension among 50 participants using injectable contraception (10%) and seven participants with hypertension among 43 participants (14%) using non‐hormonal contraception (copper IUDs); statistical comparisons were not reported (Taneepanichskul et al., 1999). None of the RCTs reported hypertension incidence.

##### NOHC use compared to OC use

Hypertension incidence was reported in one observational study and the criteria for hypertension was not defined. Two of 27 (7.4%) participants using injectactable contraception were reported to have had hypertension and 4 of 35 (11.4%) participants using the OC were reported to have hypertension; statistical comparisons were not reported(Avila et al., 1996) None of the RCTs reported hypertension incidence.

#### 2.4.2 Systolic blood pressure: Observational studies

##### NOHC use compared to non‐hormonal contraceptive use

**FIGURE 2:**
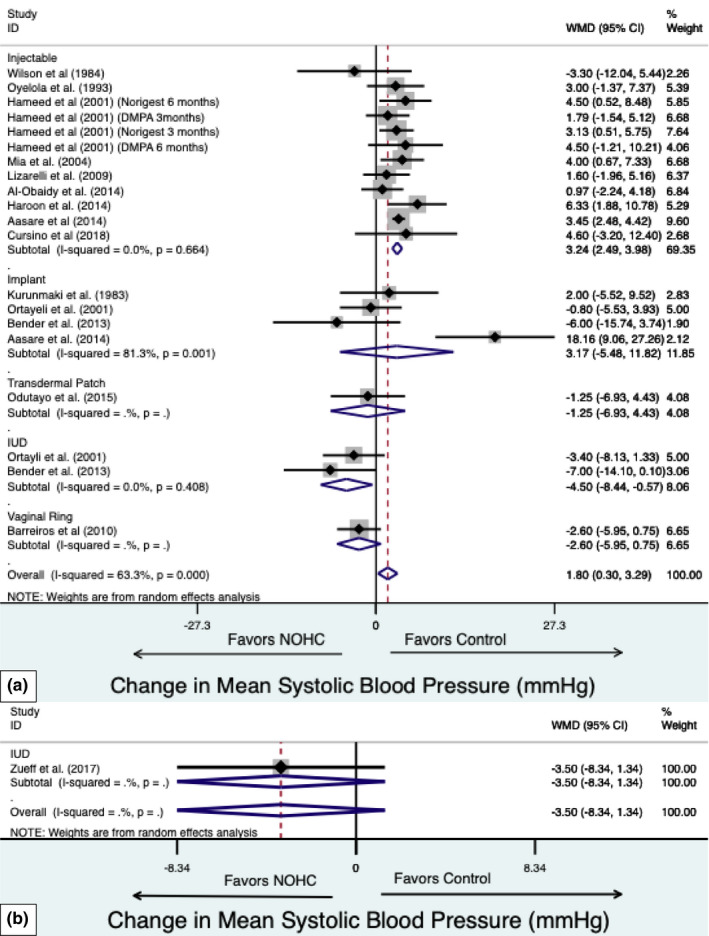
Forest plot of weighted mean difference (WMD) of the systolic blood pressure between non‐oral hormonal contraceptives (NOHC) and non‐hormonal contraceptive controls in (a) observational studies and (b) randomized controlled trials. The study specific WMD is denoted by black diamonds and the black lines indicate the 95% CI. The combined WMD by NOHC type and overall is represented by a blue diamond, the diamond width indicates the 95% CI

Injectable contraceptive use was associated with increased SBP (3.24 mmHg; 95% CI, 2.49 to 3.98 mmHg. Figure 2). No difference in SBP was observed with subdermal implant contraceptive use (3.17 mmHg; 95% CI, −5.48 to 11.82 mmHg). IUD use was associated with decreased SBP (−4.50 mmHg; 95% CI, −8.44 to −0.57 mmHg) and SBP with vaginal ring use was not different (−2.60 mmHg; 95% CI, −5.95 to 0.75 mmHg) compared to controls. Statistical heterogeneity among these studies was significant (I^2^ = 64%, *p* < 0.05).

##### NOHC use compared to OC use

Compared to OC use, injectable and implant contraceptive use was not associated with differences in SBP (Injectable: −0.56 mmHg [−2.09, 0.96]; Implant: 6.29 mmHg [−7.25, 19.83], Supplementary Data Figure S2).

#### 2.4.3 Systolic blood pressure: RCTs

No differences were observed in SBP with hormonal IUD use compared with non‐hormonal contraceptive use controls (−3.50 mmHg; 95% CI, −8.34 to 1.34 mmHg, Figure 2) and OC use (−6.05 mmHg; 95% CI, ‐17.61 to 5.51 mmHg, Supplementary Figure S2).

#### 2.4.4 Diastolic blood pressure: Observational studies

##### NOHC use compared to non‐hormonal contraceptive use

Injectable contraception use was associated with increased DBP (3.15 mmHg; 95% CI, 0.09–6.20 mmHg, Supplementary Figure S1) compared to non‐hormonal contraceptive use (controls). No difference in DBP was observed between subdermal implant use and non‐hormonal contraceptive use (3.17 mmHg; 95% CI, −3.60 to 9.94 mmHg). Both hormonal IUD (−7.48; 95% CI, −14.90 to −0.05 mmHg) and vaginal ring use (−3.90 mmHg; 95% CI, −6.67 to −1.13 mmHg) were associated with reduced DBP compared to non‐hormonal contraceptive use. Statistical heterogeneity among these studies was significant (I^2^ = 93.7%, *p* < 0.05).

##### NOHC use compared to OC use

**FIGURE 3:**
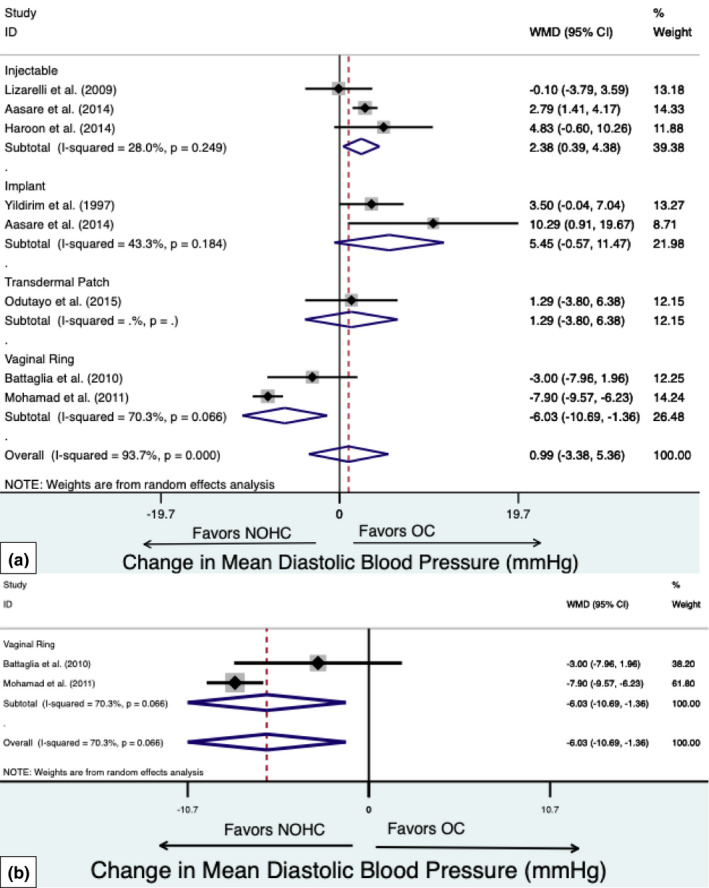
Forest plot of weighted mean difference (WMD) of the diastolic blood pressure between non‐oral hormonal contraceptives (NOHC) compared to oral contraceptive users in (a) observational studies and (b) randomized controlled trials. The study specific WMD is denoted by black diamonds and the black lines indicate the 95% CI. The combined WMD by NOHC type and overall is represented by a blue diamond, the diamond width indicates the 95% CI

Injectable contraception users demonstrated an increased DBP of 2.38 mmHg (95% CI, 0.39–4.38 mmHg. Figure 3) compared to OC users. No difference in DBP were observed with implant (5.45 mmHg; 95% CI, −0.57 to 11.47 mmHg) use compared to OC use. There was no significant heterogeneity among these studies (I^2^ = 27.5% *p* > 0.05).

#### 2.4.5 Diastolic blood pressure: RCTs

##### NOHC use compared to non‐hormonal contraceptive use

No differences were observed in DBP with hormonal IUD use compared with non‐hormonal contraceptive use (−3.50 mmHg; 95% CI, −8.34 to 1.34 mmHg) (Supplementary Figure S1).

##### NOHC use compared to OC use

Vaginal ring use compared to OC use was associated with a decrease in DBP (−6.03 mmHg; 95% CI, −10.69 to ‐5.51 mmHg, Figure 3). There was no significant heterogeneity among these studies (I^2^ = 70.3% *p* > 0.05).

## 3 DISCUSSION

We conducted a systematic review and meta‐analysis to summarize the associations between non‐oral hormonal contraceptive use, risk of hypertension and changes in blood pressure compared to non‐hormonal contraceptive use and oral contraceptive use. Our findings suggest variability in the association between systemic hormonal contraception and measures of blood pressure, whereas non‐systemic hormonal contraception was associated with decreased blood pressure. The key findings were: (1) Injectable hormonal contraceptive use was associated with a reduced risk of hypertension compared to either non‐hormonal contraceptive or oral contraceptive use, although the statistical signficance was unclear; (2) injectable hormonal contraceptive use was associated with increased measures of SBP and DBP compared to both non‐hormonal contraceptive and oral contraceptive use; (3) the implant and the transdermal contraceptives were not associated with changes in BP compared to non‐hormonal contraceptive or OC use; (4) hormonal IUD use was associated with decreased SBP compared to non‐hormonal contraceptive use; (5) vaginal ring use was associated with decreased measures of DBP compared to non‐hormonal contraceptive and OC use. Taken together, our results suggest that different forms of hormonal contraception are associated with varying impacts on blood pressure. Putting this into clinical context, there is a stepwise increase in cardiovascular risk with increasing SBP levels beginning at an SBP level as low as 90 mmHg (Whelton et al., 2020). Given the average female reproductive lifespan is approximately 37 years (Bjelland et al., 2018), contraceptive choice may have important cardiovascular health implications.

While OC use is associated with both hypertension (Chasen‐Taber et al., 1996; Chiu & Lind,; Nichols et al., 1993; Shufelt & LeVee, 2020; Stampfer et al., 1990) and a modest increase in blood pressure (Ahmed et al., 2004; Davis et al., 2017; Lewis et al., 1997; Meade et al., 1977), few studies have examined the effects of NOHC use on blood pressure. Previous studies have shown that OC use is associated with both higher blood pressure and circulating levels of RAAS components as well as a more robust blood pressure response to Angiotensin II challenge compared to healthy controls (Ahmed et al., 2004). In contrast, a cross‐sectional study comparing 10 normotensive transdermal patch contraceptive users to 10 OC users and 15 controls demonstrated similar blood pressure values across groups. Transdermal patch contraceptive users had lower levels of circulating RAAS components and an impaired ability to maintain blood pressure in response to lower body negative pressure compared to controls and OC users (Odutayo et al., 2015). These results suggest that non‐oral, and specifically transdermal, routes of estrogen administration may result in RAAS suppression though this remains speculative.

Our results suggest that the effects of NOHC use on blood pressure differ, at least in part, based on the route of administration. Injectable contraception was potentially associated with a lower incidence of hypertension, but increased measures of SBP and DBP, compared to non‐hormonal contraceptive and OC use, while use of the subdermal implant was associated with similar SBP and DBP compared to non‐hormonal contraceptive and OC use. Conversely, non‐systemic forms of NOHC such as the hormonal IUD and vaginal ring were associated with lower measures of blood pressure. We hypothesize that these findings may also be, in part, due to the type, dose, and presence or absence of estrogen and progestins present in the different forms of contraception (Grow, 2002; Sitruk‐Ware, 2000).

The pharmacokinetics and efficacy of different estrogens vary considerably, which likely translates into different vascular and metabolic effects. (Grow, 2002; Sitruk‐Ware, 2000) However, ethinyl estradiol was the only type of synthetic estrogen present in the contraceptives included in this study. The dose of synthetic estrogens in the OC has been shown to modify cardiovascular risk (Boldo & White, 2011); this may at least partially explain the decreased BP observed with hormonal IUD (which does not contain any synthetic estrogen) use in our study. However, other progestin‐only contraceptives in our study, such as the injectable contraceptive, were associated with greater blood pressure, highlighting the potentially important role of progestin type. Progestins are synthetic forms of endogenous progesterone and can be derived from either 17‐a‐hydroxyprogesterone or 19‐nortestosterone (Ghatge et al., 2005; Oelkers, 1996) resulting in wide variation in both progestational and androgenic activity (Sarna et al., 2009; Sitruk‐Ware, 2004; Sitruk‐Ware et al., 2004). High androgenic activity, predominately present in testosterone‐derived progestins, is associated with higher cardiovascular risk (Nath & Sitruk‐Ware, 2009; Sitruk‐Ware, 2000). Depot medroxyprogesterone acetate (DMPA) is derived from natural progesterone but has high androgenic potency (Ghatge et al., 2005; Nath & Sitruk‐Ware, 2009; Sitruk‐Ware, 2000). In vitro (Zerr‐Fouineau et al., 2009), in vivo (Williams et al., 1998) and clinical human data have shown that DMPA inhibits the cardioprotective benefits of estrogen (Gupta, 2003) and is independently associated with endothelial dysfunction, including reduced flow‐mediated dilation (Lizarelli et al., 2009; Sorensen et al., 2002). In keeping with this literature, our results suggest that contraceptives containing DMPA (e.g., injectable contraception), are associated with increased measures of blood pressure, while progestins with low androgenic activity, such as levonorgestrel (e.g., hormonal IUD), are associated with decreased measures of blood pressure.

This study has strengths and limitations. First, hypertension and blood pressure were secondary outcomes in studies investigating the safety and efficacy of NOHC use. Furthermore, there was a lack of clarity on the definition of hypertension and variation in the methods of blood pressure measurement across studies. Second, there was significant heterogeneity in the study populations with some studies restricting enrolment to healthy women or women with specific comorbidities with significant variation in socioeconomic position. It is important to note the potential bias in the I^2^ statistic when the number of studies is small, therefore the point estimate I^2^ should be interpreted cautiously (Hippel, 2015). However, given the widespread and global use of hormonal contraception, this may increase the generalizability of this review’s results. Of note, the majority of included studies were of low to moderate quality and the outcomes of interest were continuous variables making an evaluation of publication bias challenging (Doleman et al., 2020). Third, the duration of included studies varied from 3 to 12 months, while in cross‐sectional studies, the exposure time to specific contraceptives was not always reported. Therefore, we were unable to compare the exposure time across studies. Finally, the formulation of the study contraceptives varied widely in the type of progestin as well as the dose of synthetic estrogen and progestin. However, our search strategy used broad search terms with no language restrictions and provides a comprehensive review of the current literature on the associations between non‐oral hormonal contraception, risk of hypertension, and changes in blood pressure.

This systematic review and meta‐analysis found that compared to non‐hormonal contraceptive and OC use, injectable NOHC use was potentially associated with a decreased risk of hypertension but increased blood pressure while hormonal IUD and vaginal ring use were associated with decreased measures of blood pressure. Given the important cardiovascular effects of small changes in blood pressure even within the normal range (Whelton et al., 2020), the findings of this study may have widespread public health implications given the significant duration of the female reproductive lifespan. Large‐scale prospective studies will need to be performed before recommendations regarding clinical practice can be made, and individual preference remains the most important factor when choosing the most appropriate form of contraception.

## AUTHOR CONTRIBUTIONS

CZK, SMD, and SBA contributed to the concept and design of the study, statistical analyses, and manuscript preparation; JMM, KN, AM, PZ and MR contributed to interpretation of results and revision of manuscript drafts. All authors were involved in the interpretation of the results and the revision of the manuscript and approved the submitted version of the manuscript.

## Supporting information



Fig S1

Fig S2

Table S1

Table S2

Table S3

Table S4
