# Supplementary material for: The effect of non‐oral hormonal contraceptives on hypertension and blood pressure: A systematic review and meta‐analysis
Source: Physiol Rep. 2022 May 4;10(9):e15267. doi: 10.14814/phy2.15267 (PMC9069167; doi:10.14814/phy2.15267)
Supplement: Supplementary file 1 — Fig S1 [file PHY2-10-e15267-s001.docx]

Figure 1. Forest plot of weighted mean difference (WMD) of the diastolic blood pressure between non-oral hormonal contraceptives (NOHC) compared to controls in (A) observational studies and (B) randomized controlled trials. The study specific WMD is denoted by black diamonds and the black lines indicate the 95% CI. The combined WMD by NOHC type and overall is represented by a blue diamond,

the diamond width indicates the 95% CI.
