# Supplementary material for: The effect of non‐oral hormonal contraceptives on hypertension and blood pressure: A systematic review and meta‐analysis
Source: Physiol Rep. 2022 May 4;10(9):e15267. doi: 10.14814/phy2.15267 (PMC9069167; doi:10.14814/phy2.15267)
Supplement: Supplementary file 3 — Table S1 [file PHY2-10-e15267-s005.docx]

Supplementary Data

Table 1: Medline Search Strategy

1. estrogen.mp. or exp Estrogens/ or estradiol.mp. or exp Ethinyl Estradiol/ or exp Estradiol/ or exp Ethinyl Estradiol-Norgestrel Combination/
2. medroxyprogesterone Acetate.mp. or exp Medroxyprogesterone Acetate/ or exp Norgestrel/ or norelgestromin.mp. or gestagen.mp. or levonorgestrel.mp. or exp Levonorgestrel/ or exp Progestins/ or progest*.mp.
3. contraception.mp. or exp Contraception/ or ((hormone* or estrogen or oestrogen) adj1 (contracept* or therap*)).mp. or exp Contraceptive Agents, Female/ or contraceptive.mp. or exp Contraceptive Agents/ or ovulation inhibition.mp. or exp Ovulation Inhibition/ or birth control.mp.
4. 1 or 2 or 3
5. (nonoral or non-oral or non oral).mp.
6. 4 and 5
7. (patch* adj2 (transdermal or contracept* or birth control)).mp.
8. (Ortho-evra or xulane).mp.
9. ((vaginal ring adj (etonogestrel or levonorgestrel or ethinylestradiol or estrogen or estradiol)) or (ring adj2 (contracept* or vaginal))).mp.
10. Nuvaring.mp.
11. (intra-uterine adj2 (device* or system* or method* or levonorgestrel or levonorgestrel-releasing or reversible or contracept* or hormon*)).mp.
12. (intrauterine adj2 (device* or system* or method* or levonorgestrel or levonorgestrel-releasing or reversible or contracept* or hormon*)).mp.
13. intrauterine device.mp. or exp Intrauterine Devices/ or long acting reversible contraception.mp. or exp Long- Acting Reversible Contraception/ or exp Intrauterine Devices, Medicated/ or intrauterine system.mp. or exp Intrauterine Devices/ or levonorgestrel releasing.mp.
14. (Mirena or Skyla or Kyleena or Liletta).mp.
15. exp Medroxyprogesterone Acetate/ or medroxyprogesterone Acetate injection.mp. or (inject* adj2 (projest* or hormon* or contracept*)).mp.
16. (Depo-provera or Depo-subQ Provera 104).mp.
17. (etonogestrel subdermal implant or ((subdermal or subcutaneous) adj2 progest*)).mp.
18. (Implanon or nexplanon).mp.
19. ((birth control adj (vaginal ring or shot or implant or patch)) or (progest* adj (contracep* or long- acting))).mp.
20. 6 or 7 or 8 or 9 or 10 or 11 or 12 or 13 or 14 or 15 or 16 or 17 or 18 or 19
21. exp Essential Hypertension/ or exp Hypertension/ or hypertension.mp. or hypertens*.mp. or HTN.mp. or BP.mp.
22. blood pressure.mp. or exp Blood Pressure/ 23 21 or 22

24 20 and 23

25 animals/ not humans/ 26 24 not 25
