# Supplementary material for: The effect of non‐oral hormonal contraceptives on hypertension and blood pressure: A systematic review and meta‐analysis
Source: Physiol Rep. 2022 May 4;10(9):e15267. doi: 10.14814/phy2.15267 (PMC9069167; doi:10.14814/phy2.15267)
Supplement: Supplementary file 4 — Table S2 [file PHY2-10-e15267-s006.docx]

Table 2: Quality assessment of the cohort studies using New Castle Ottawa Scale

**Selection**

**Comparability**

**Outcome**

**First Author (Year)**

**1**

**2 3 4**

**5**

**6**

**7**

**8**

**Total**

|  |  |
| --- | --- |
| Sivin et al. (1981) 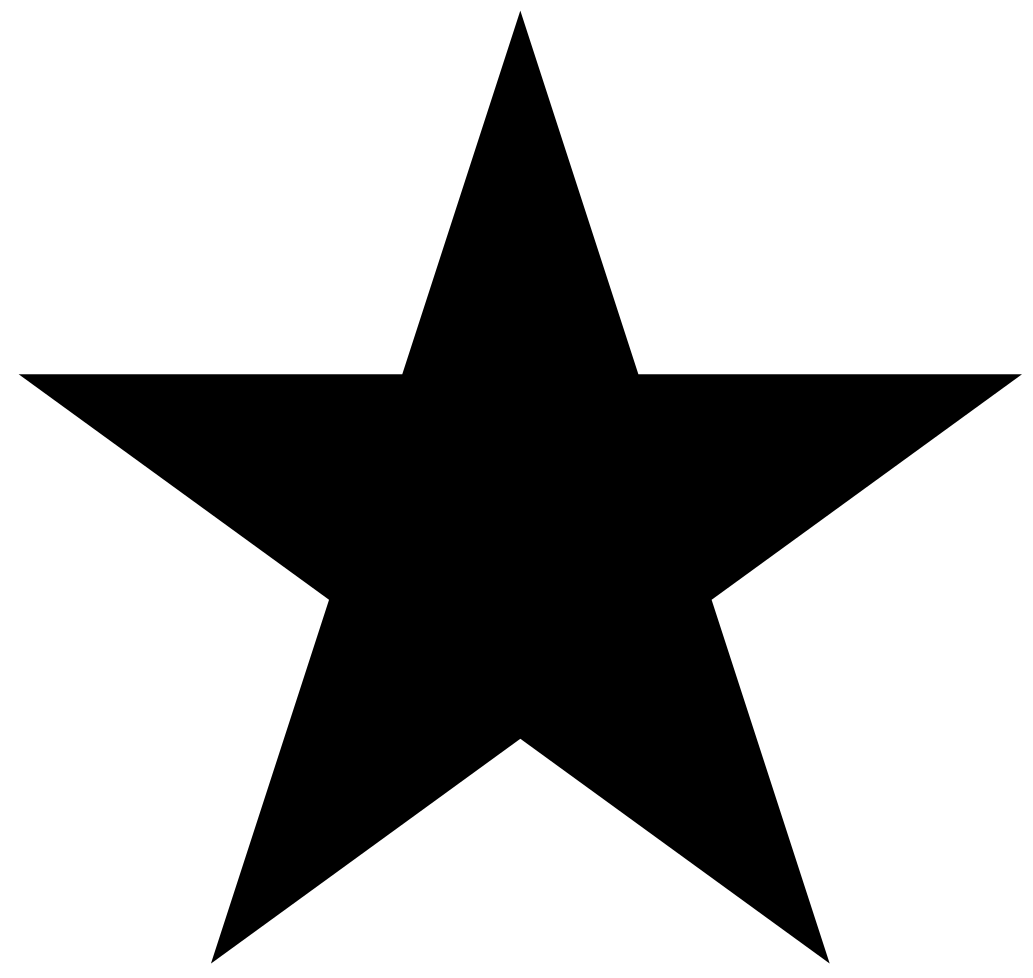 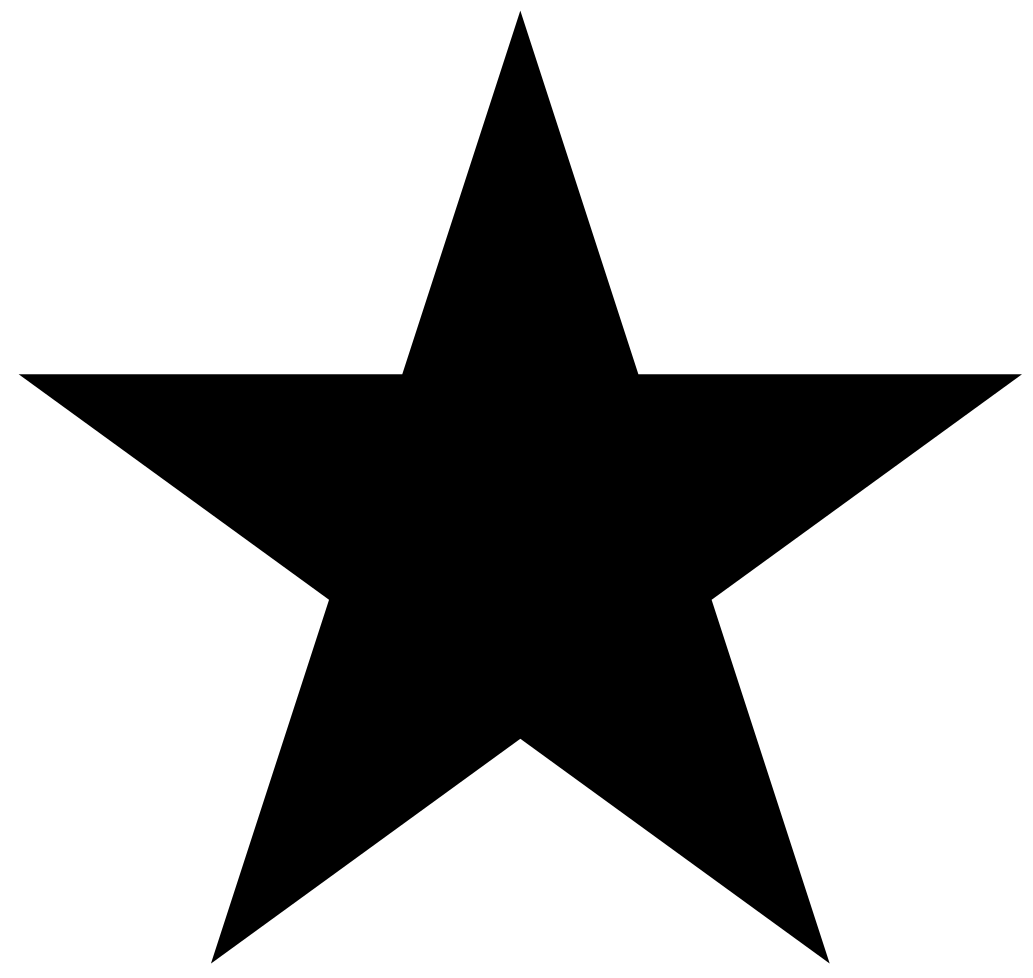 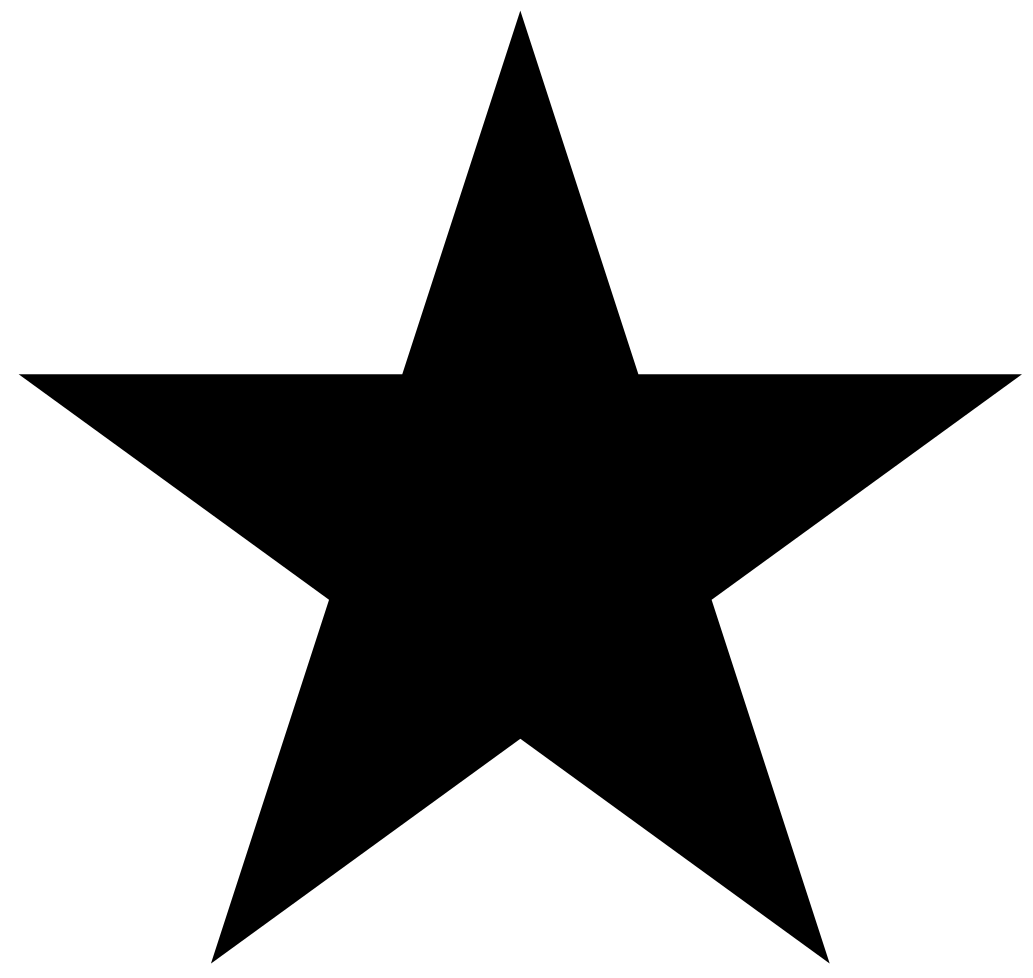 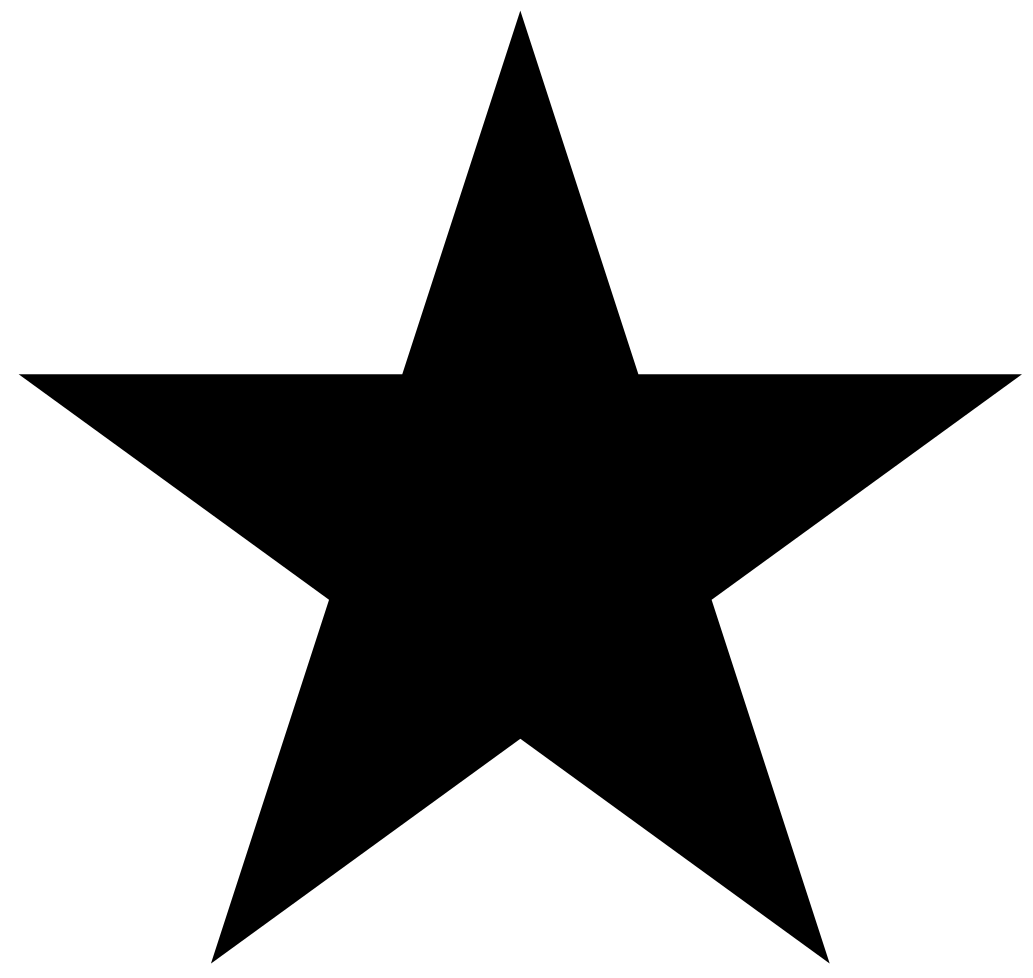 | 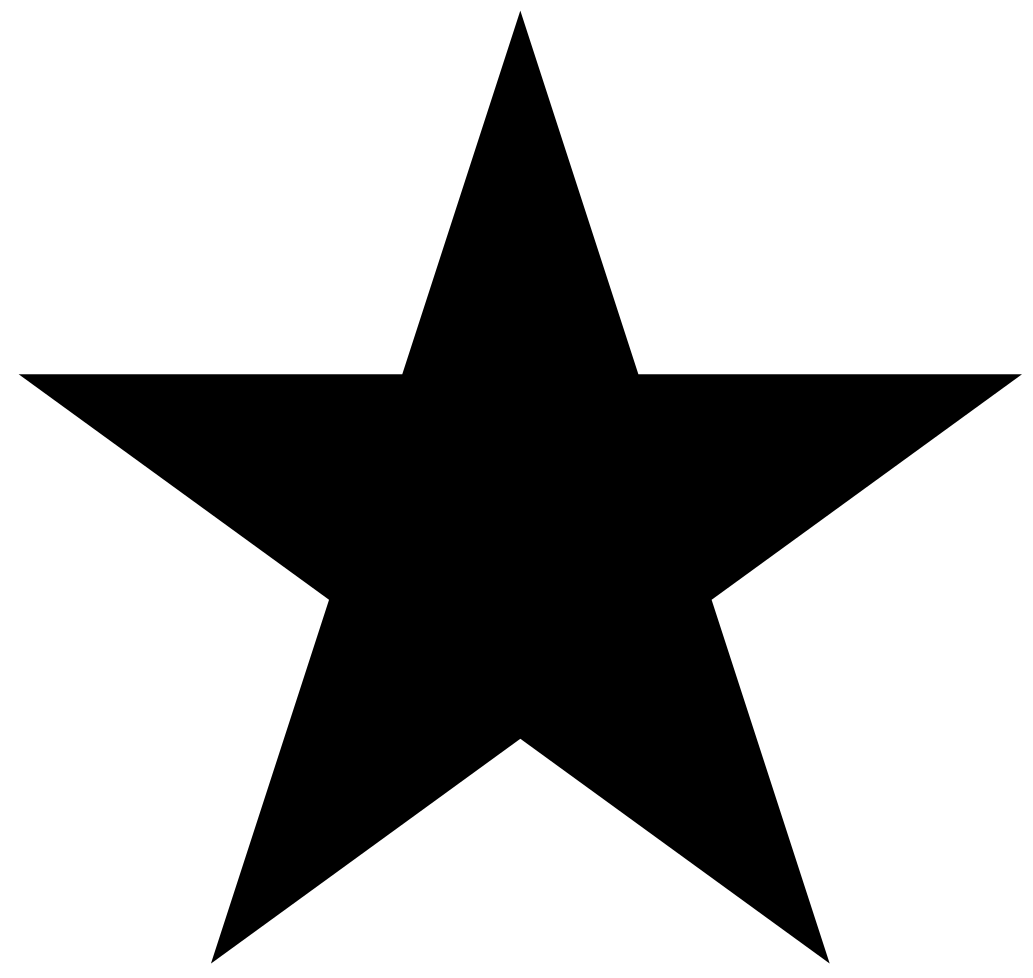 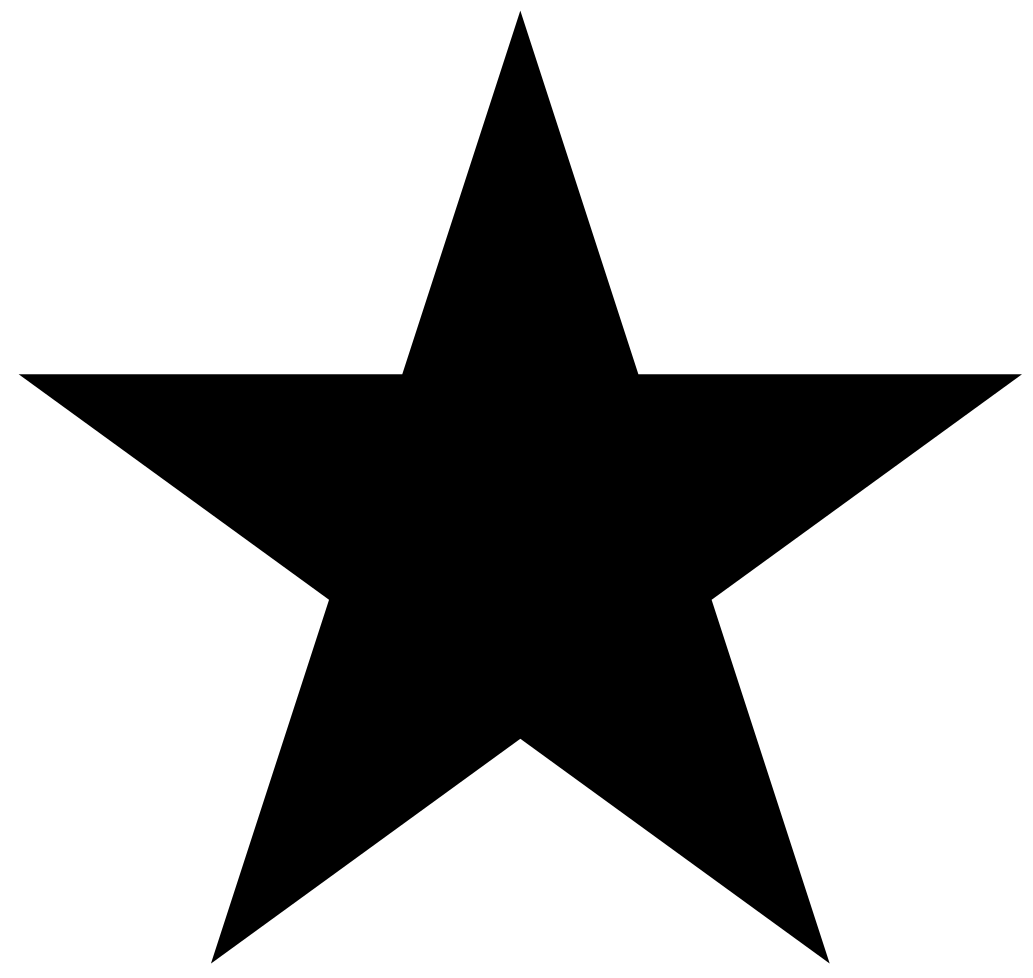 **6** |
| Kurunmaki et al. (1983) 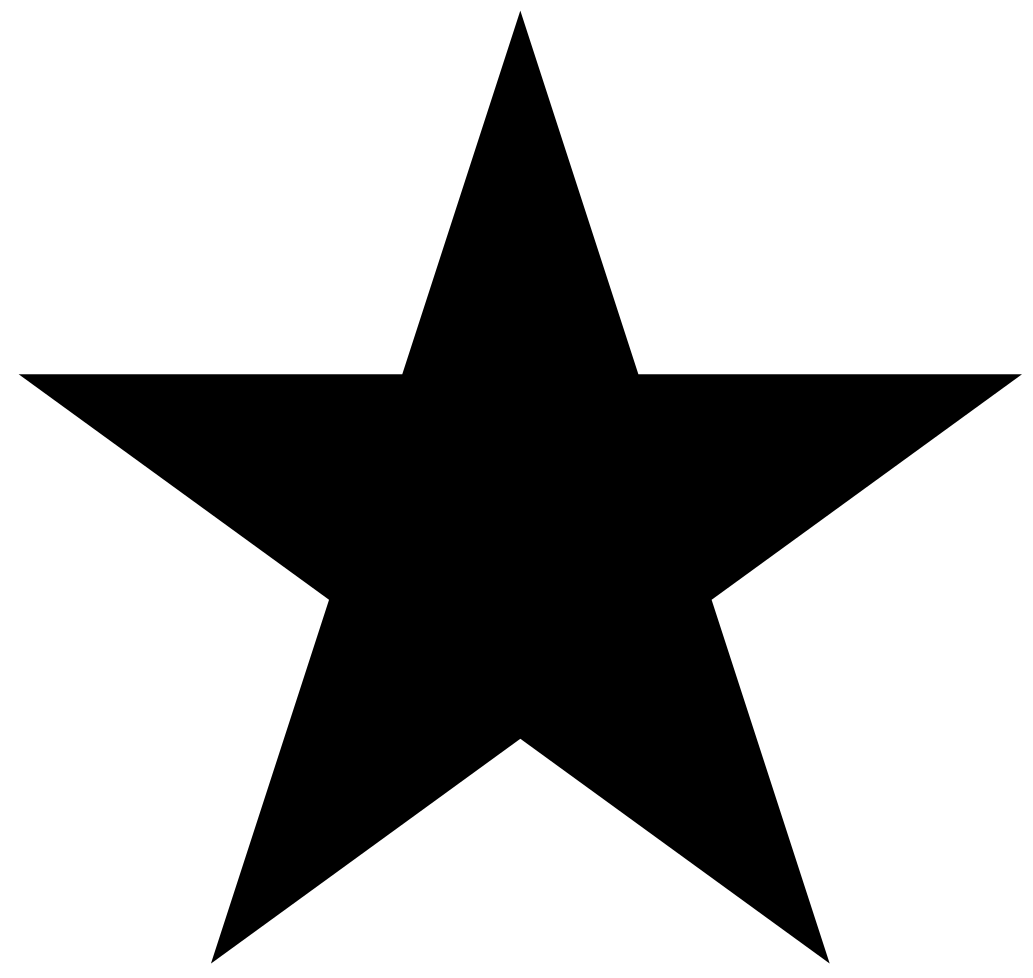 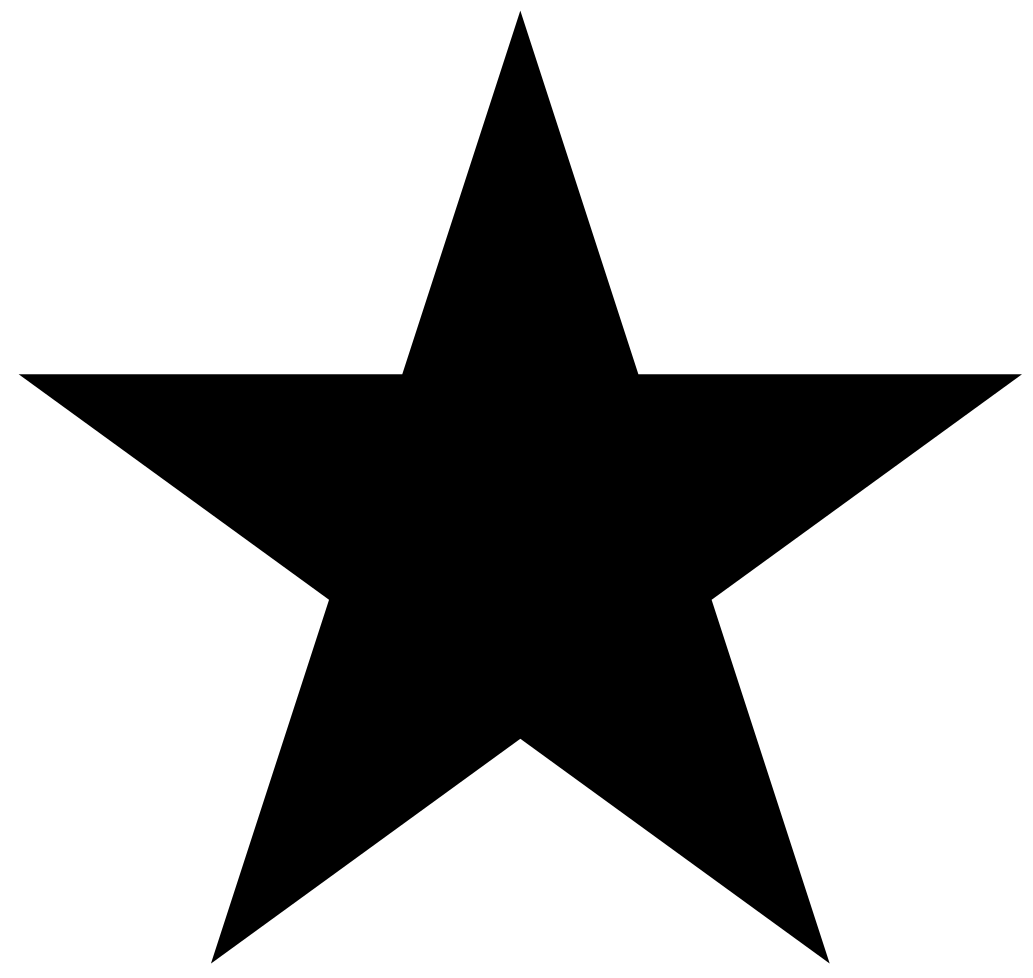 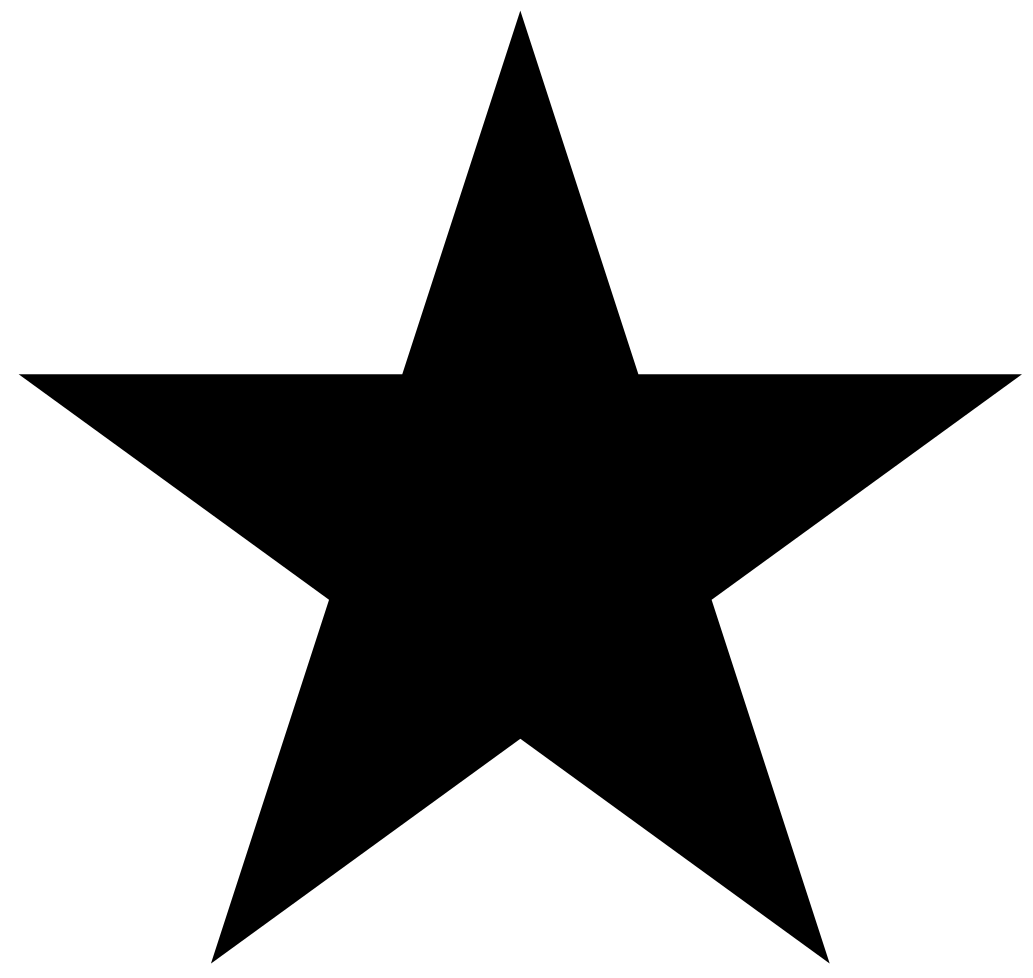 | 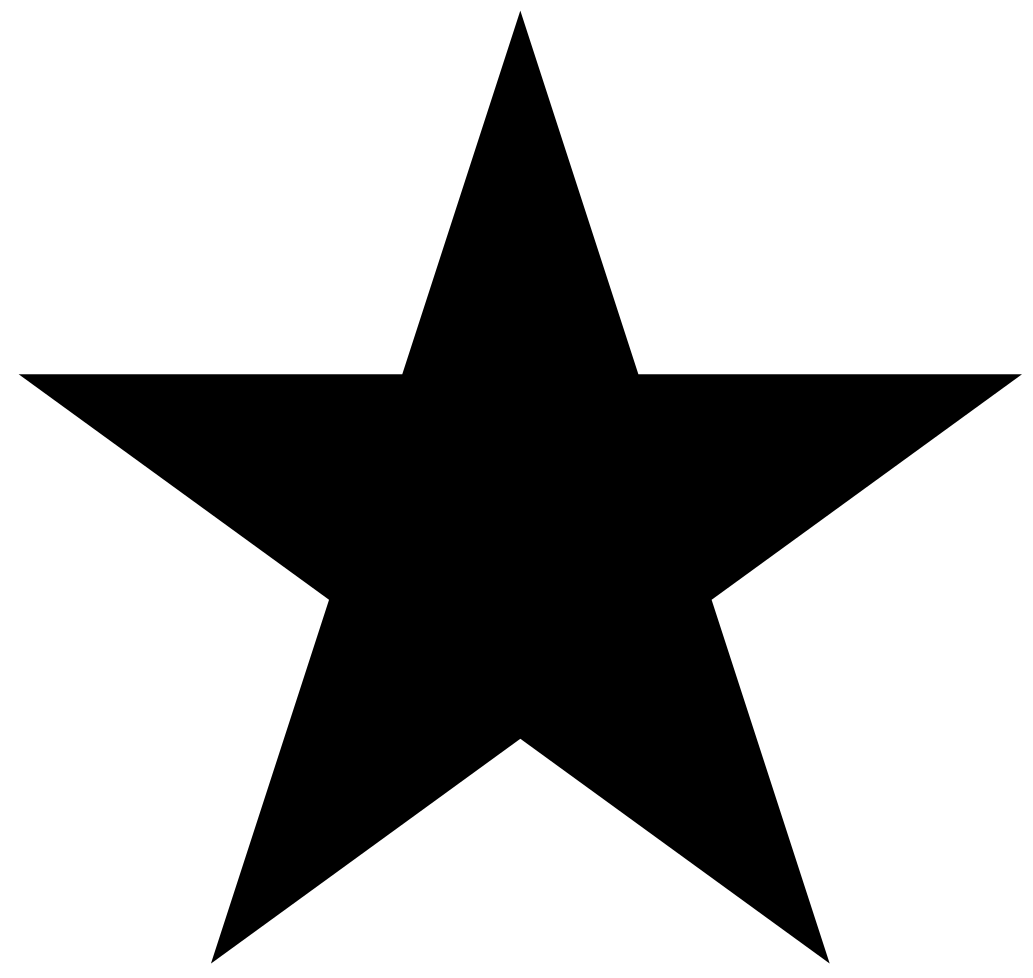 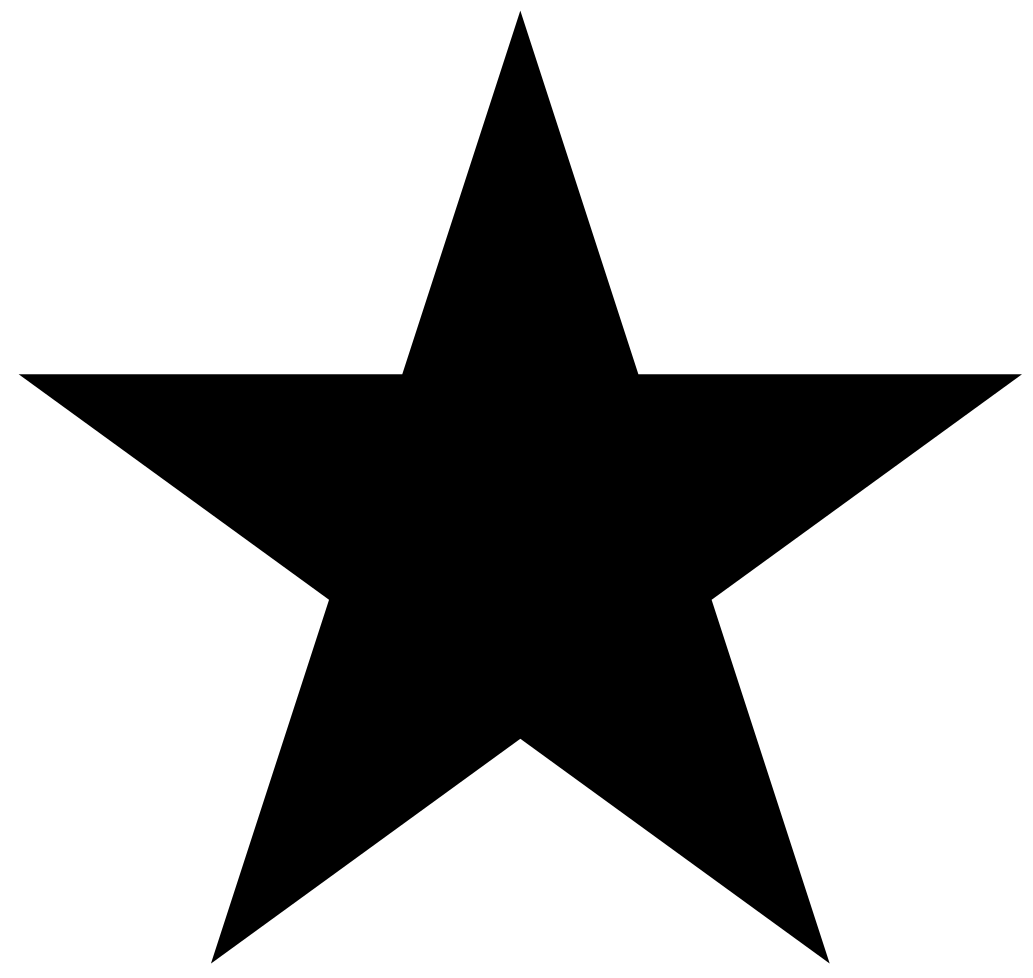 **5** |
| Wilson et al. (1984) 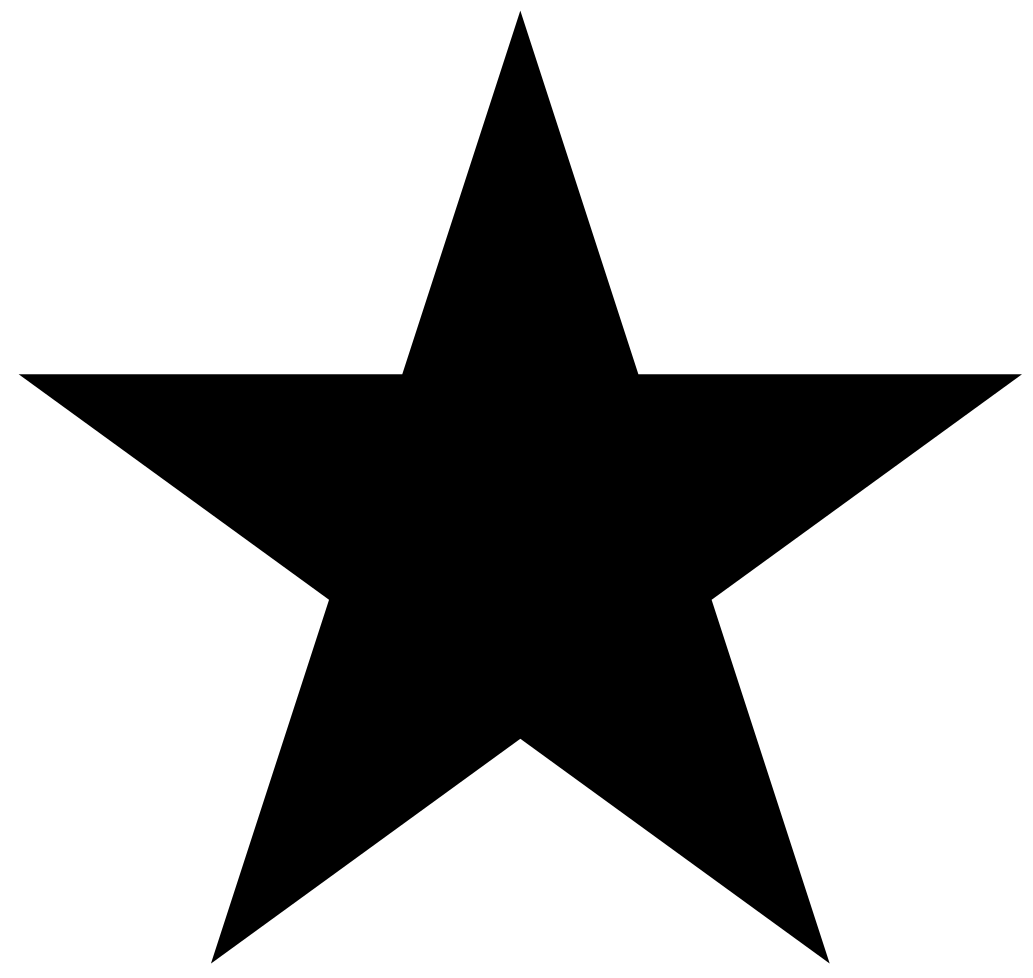 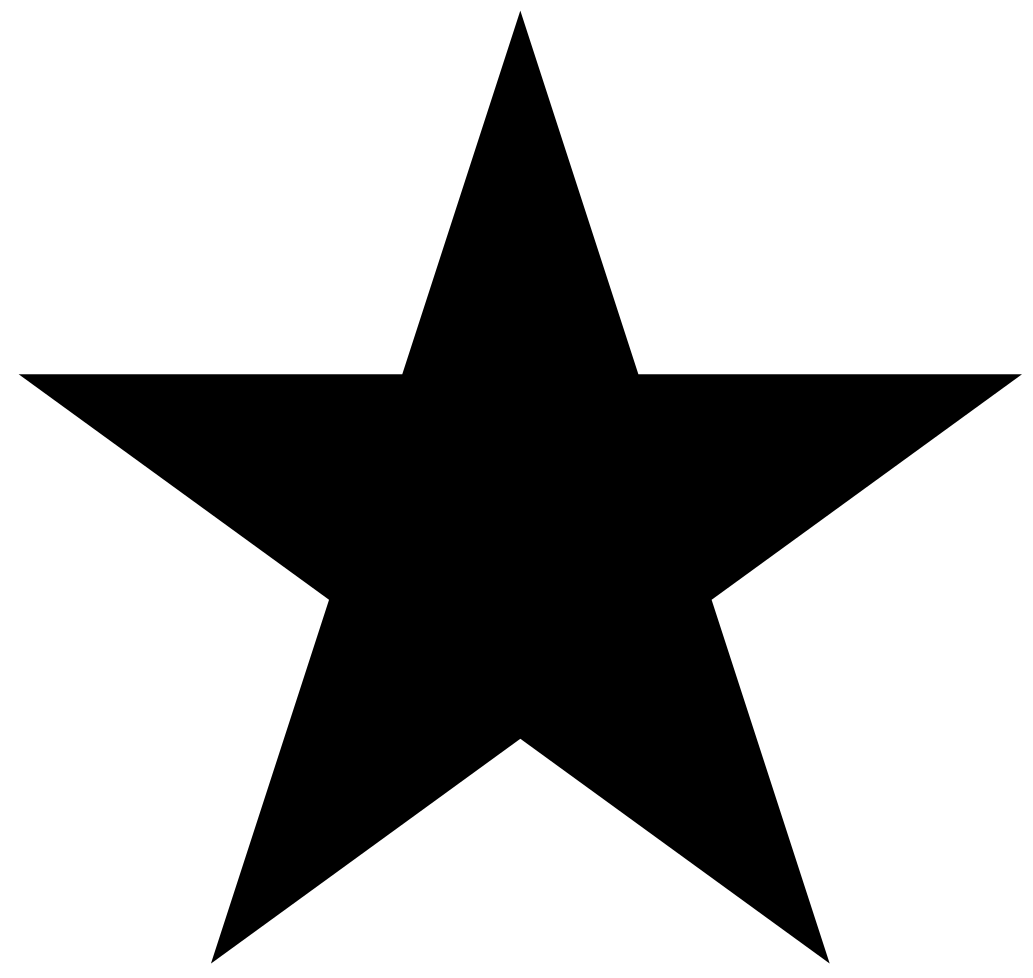 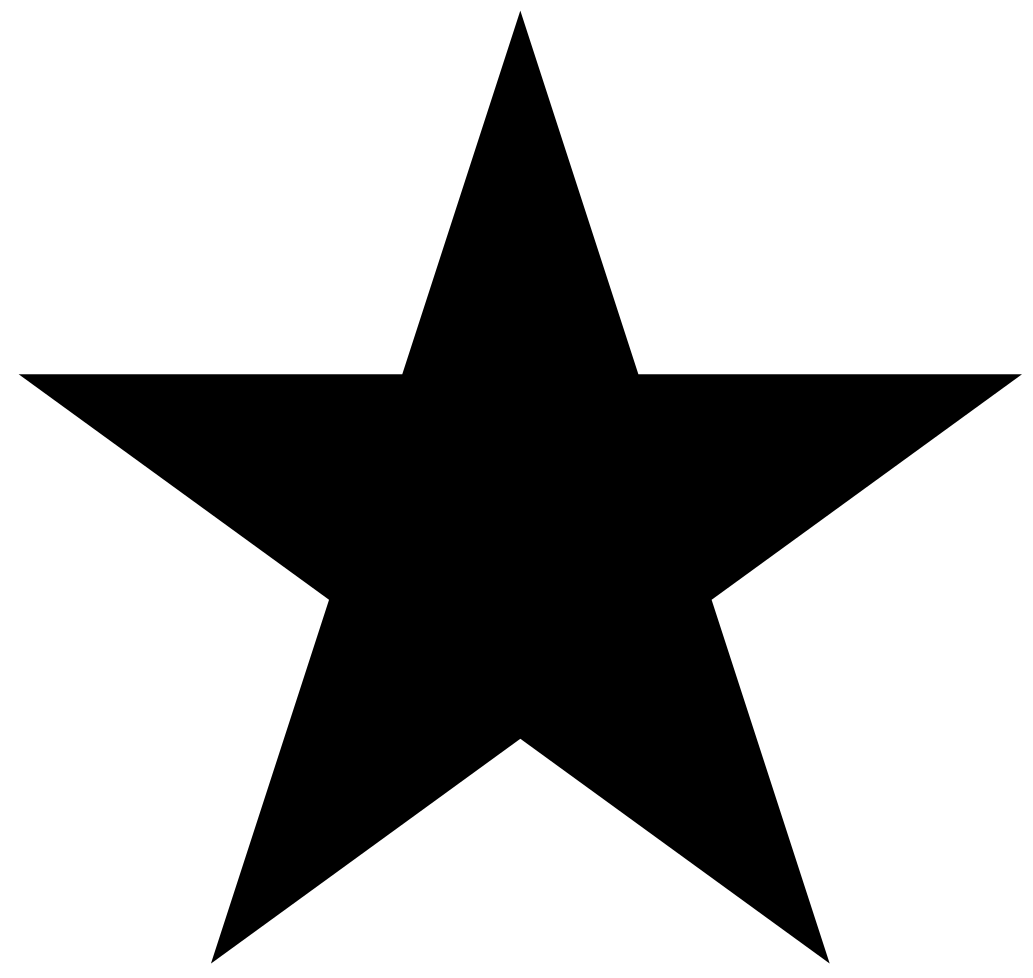 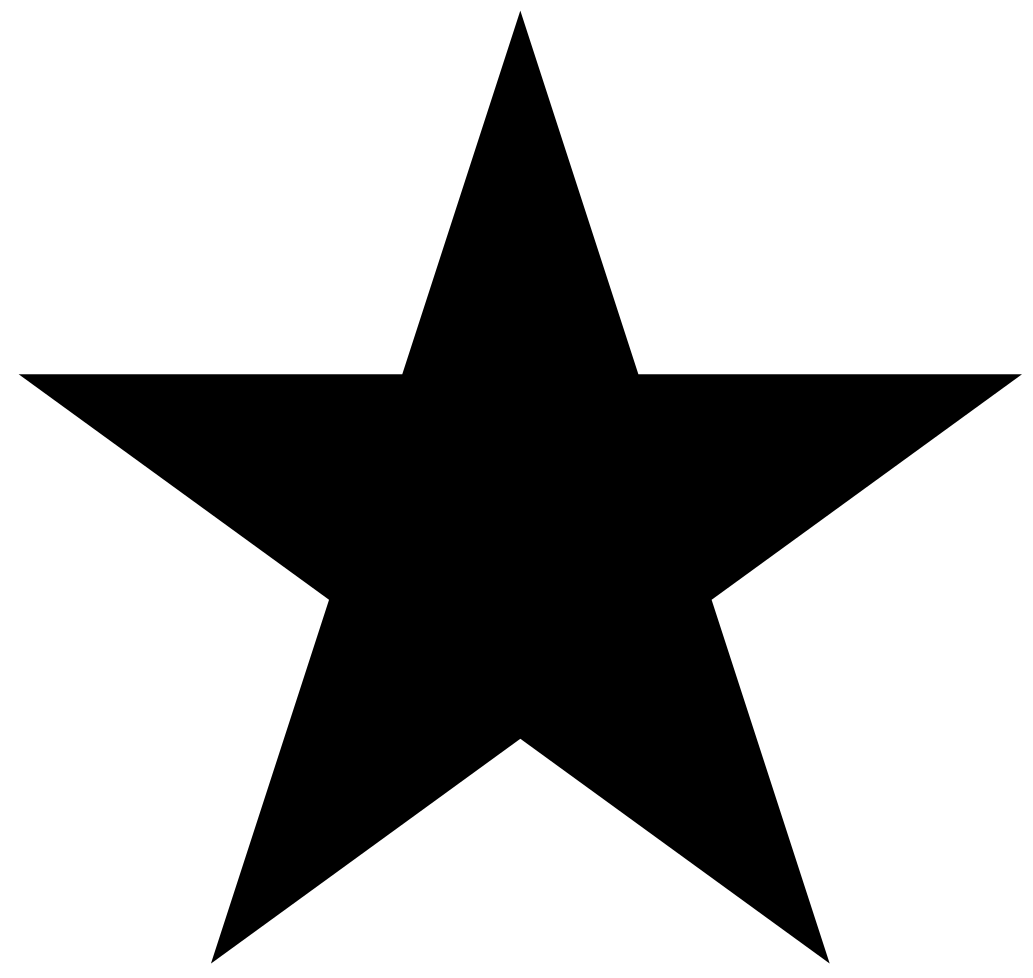 | 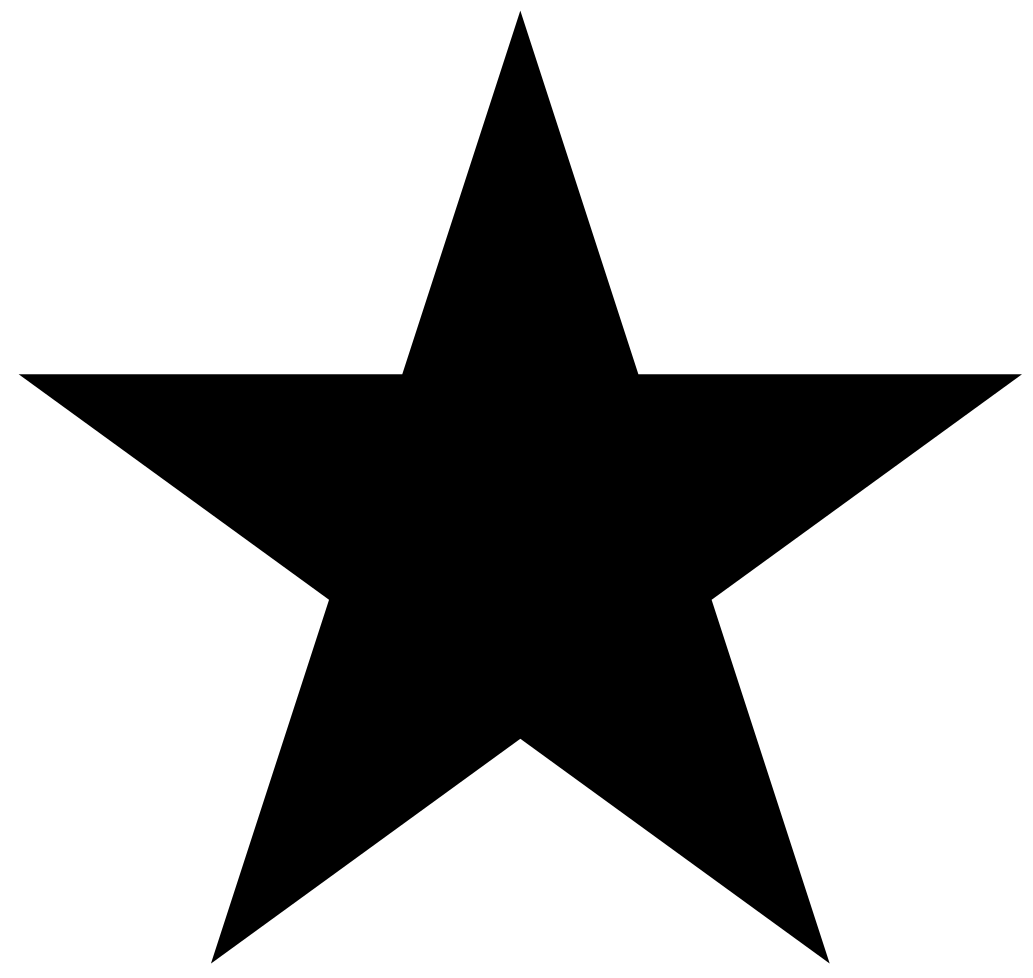 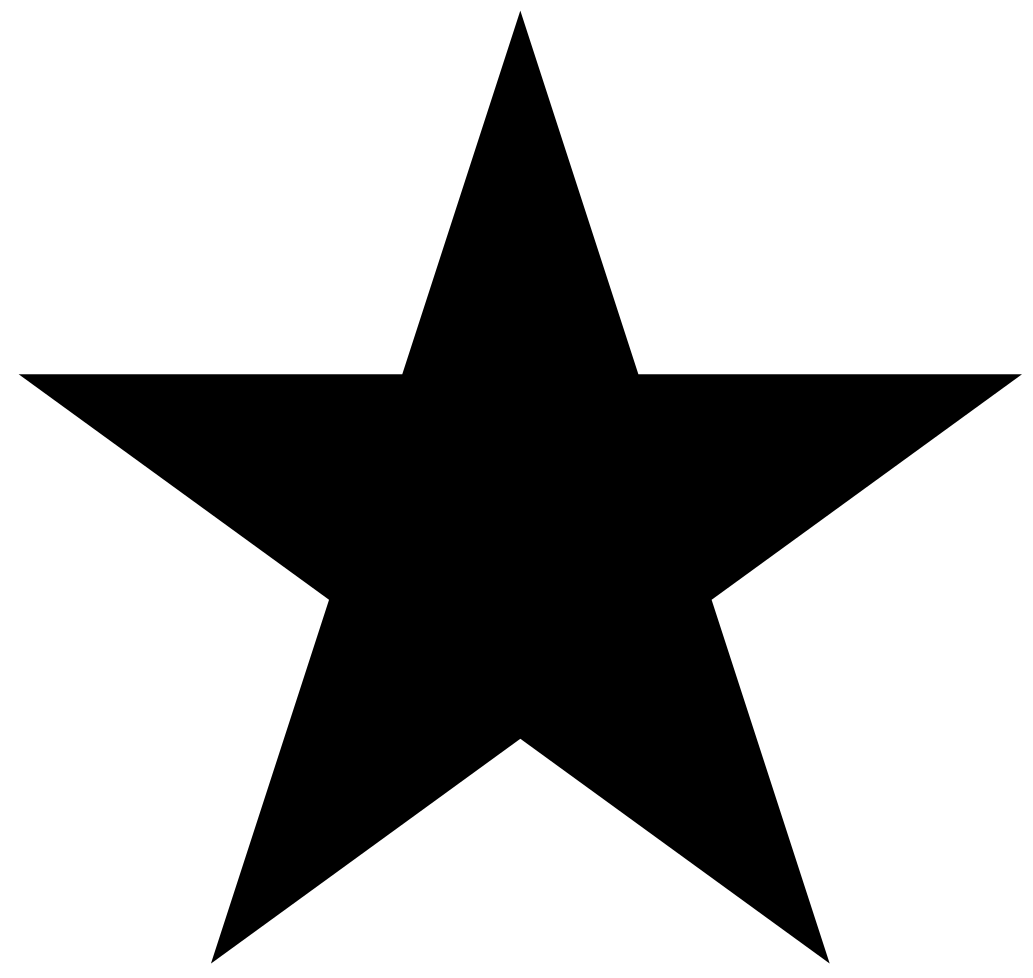 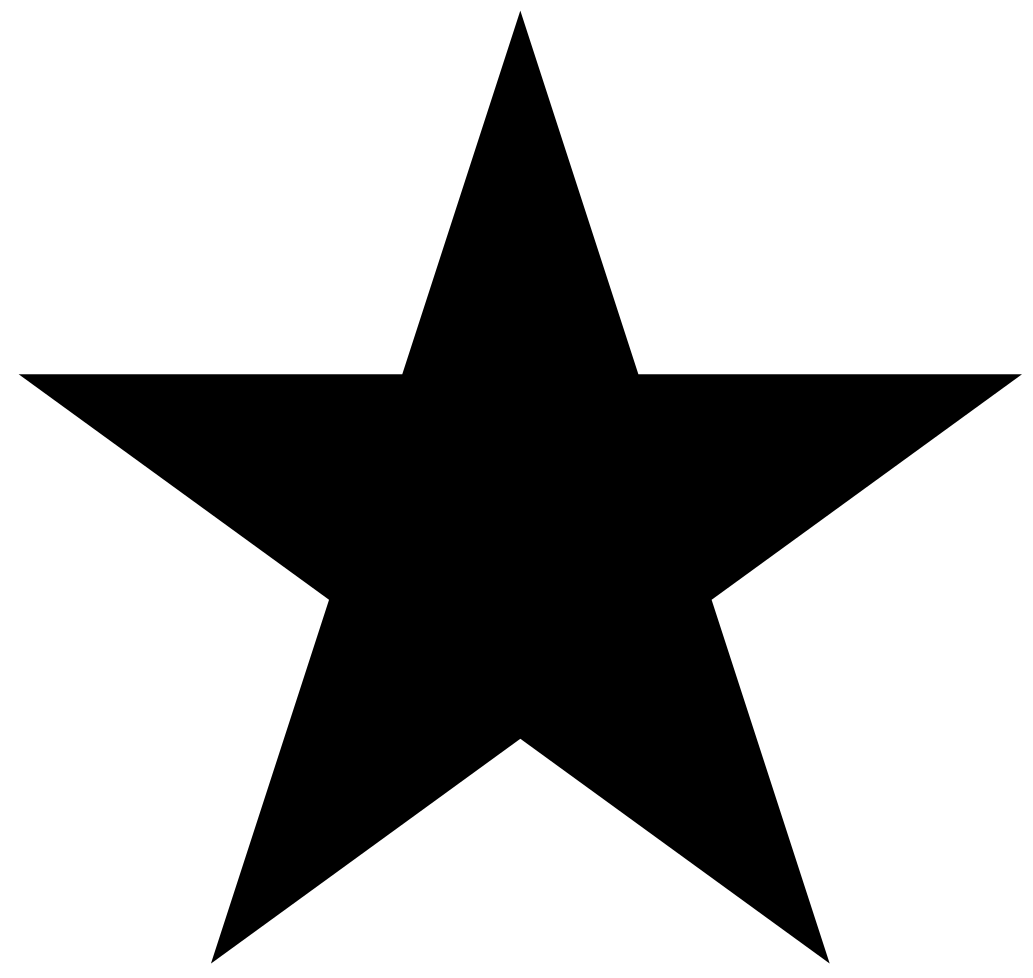 **7** |
| Qifang et al. (1994) 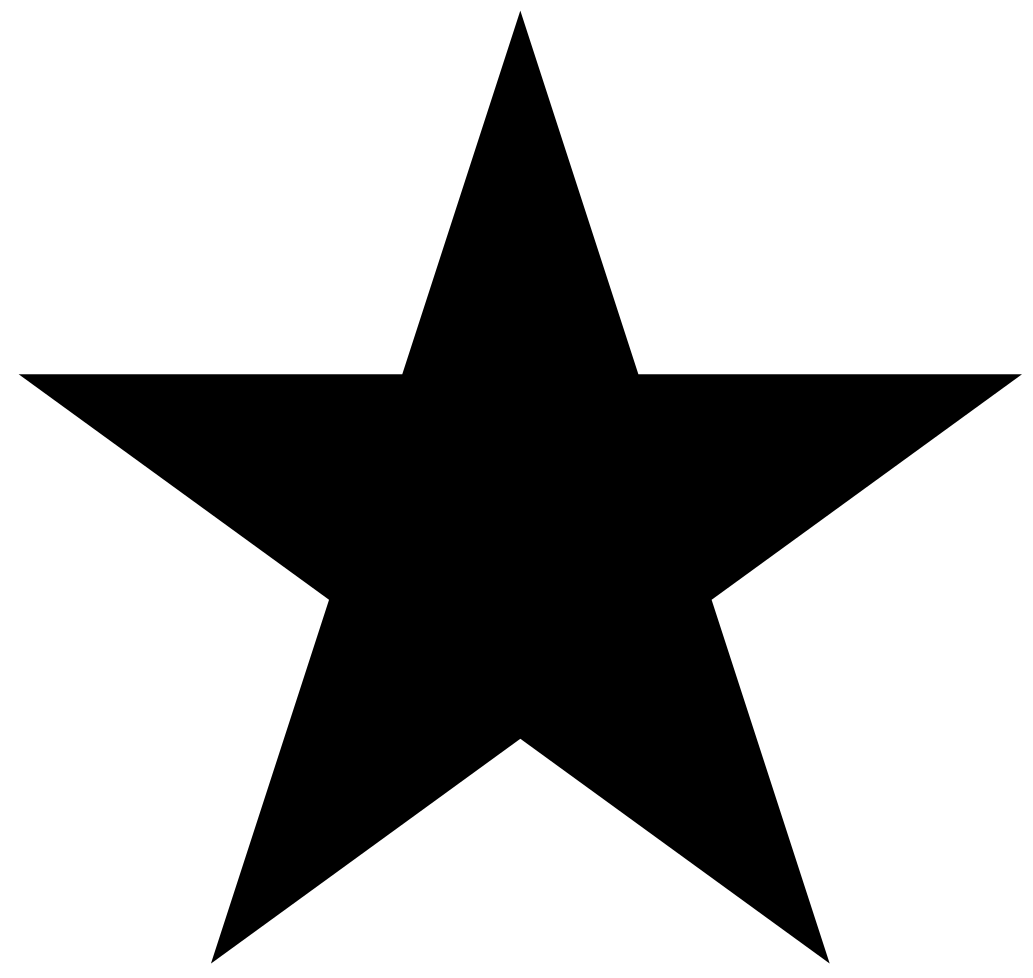 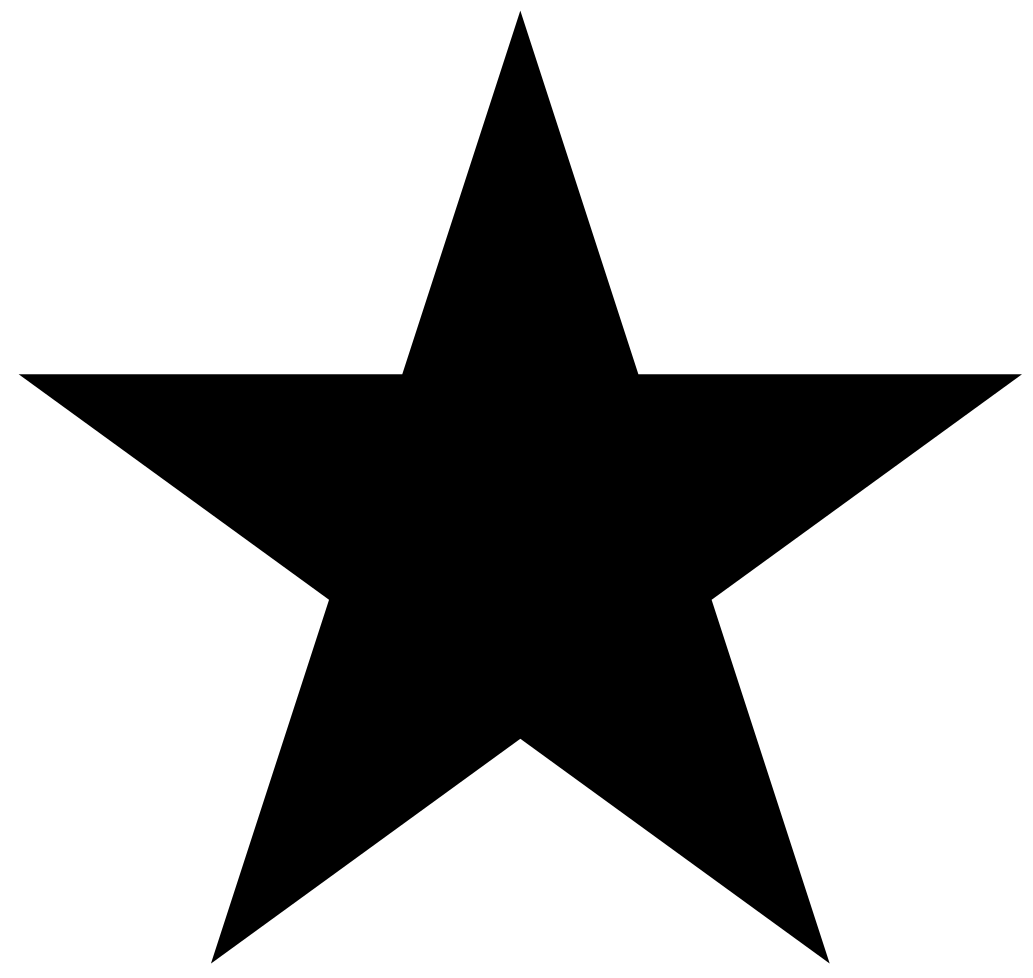 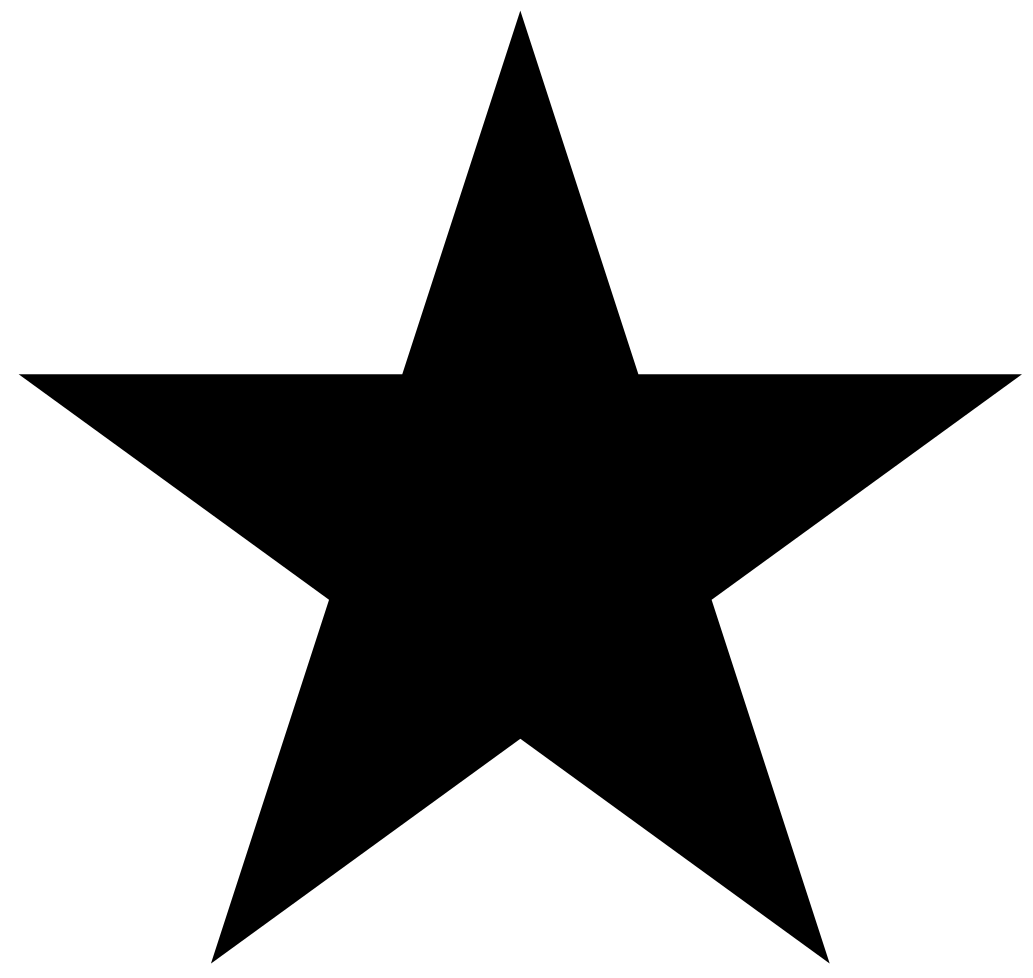 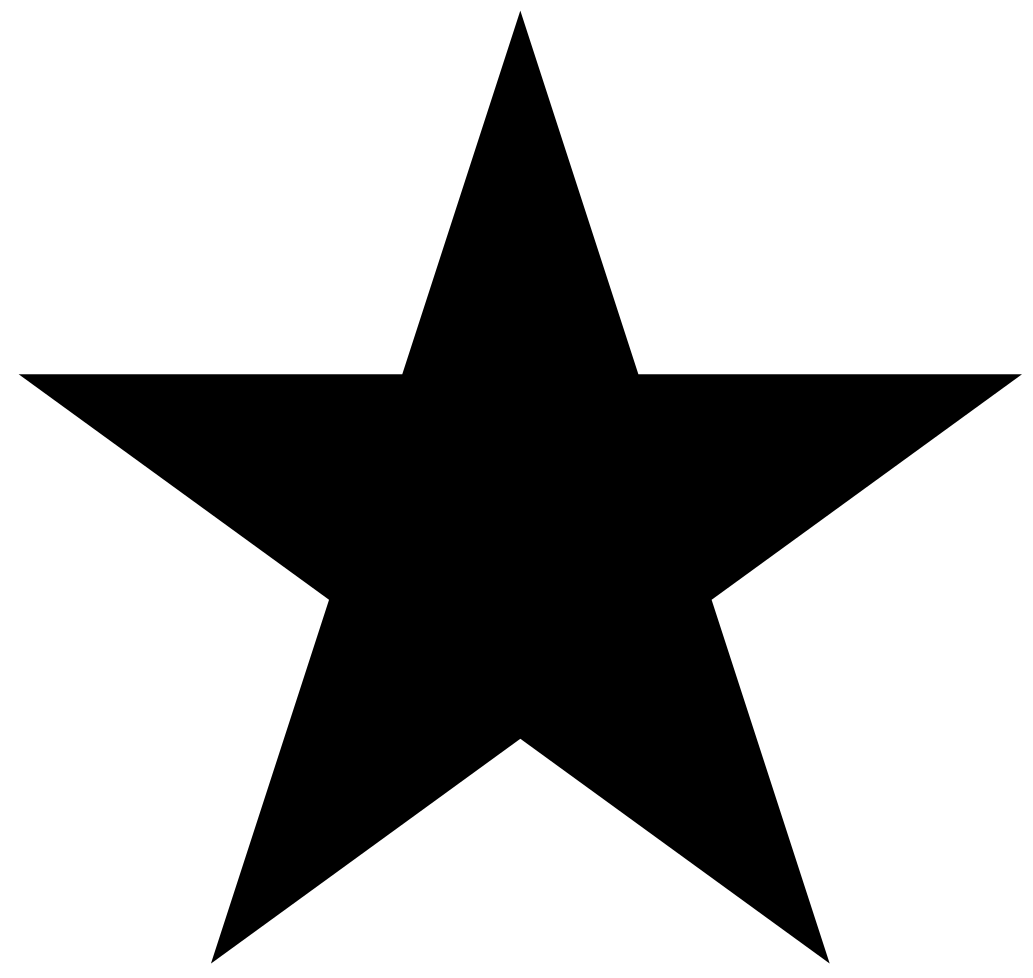 | 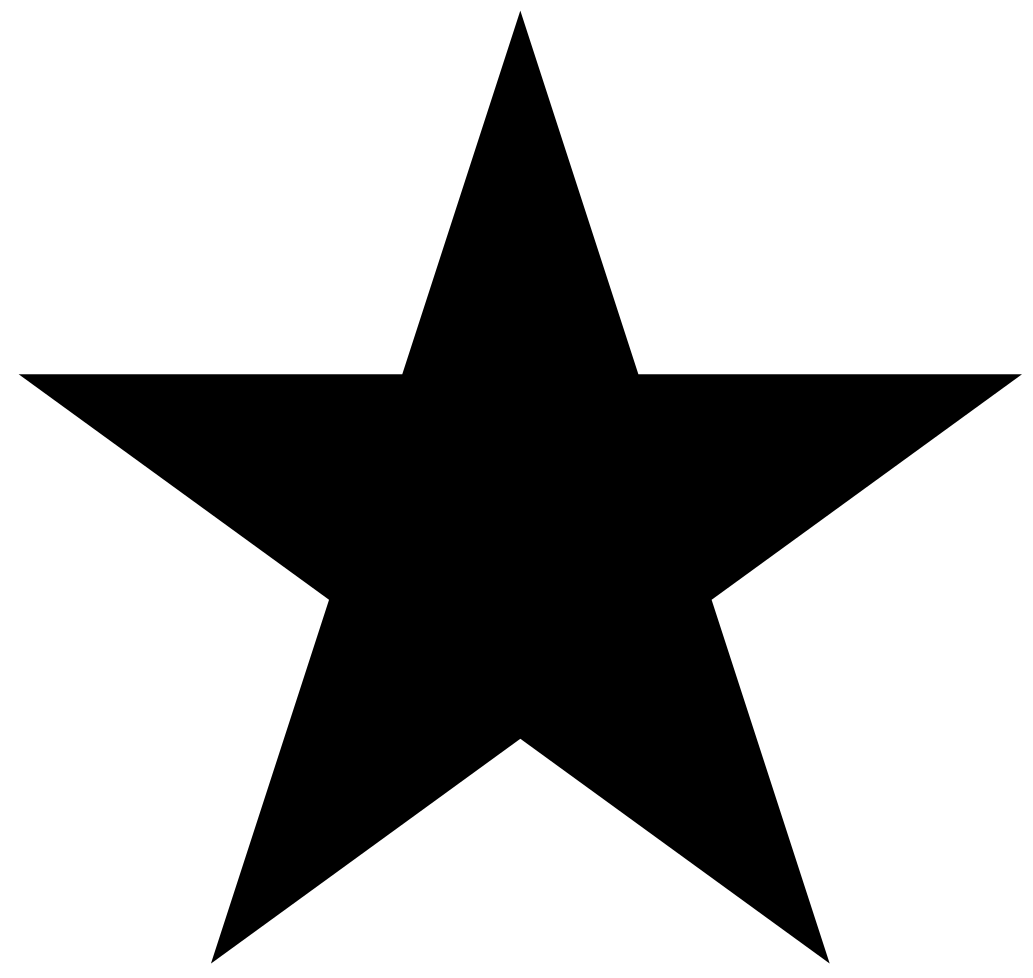 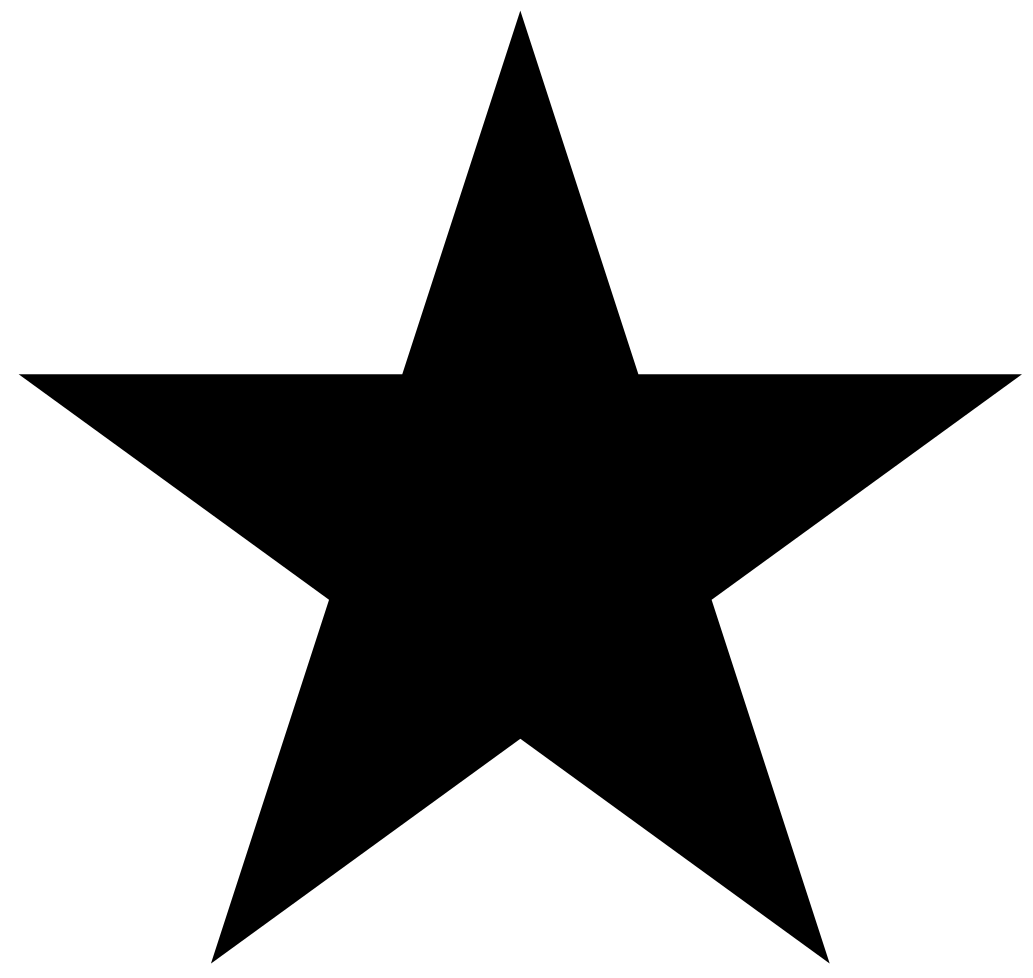 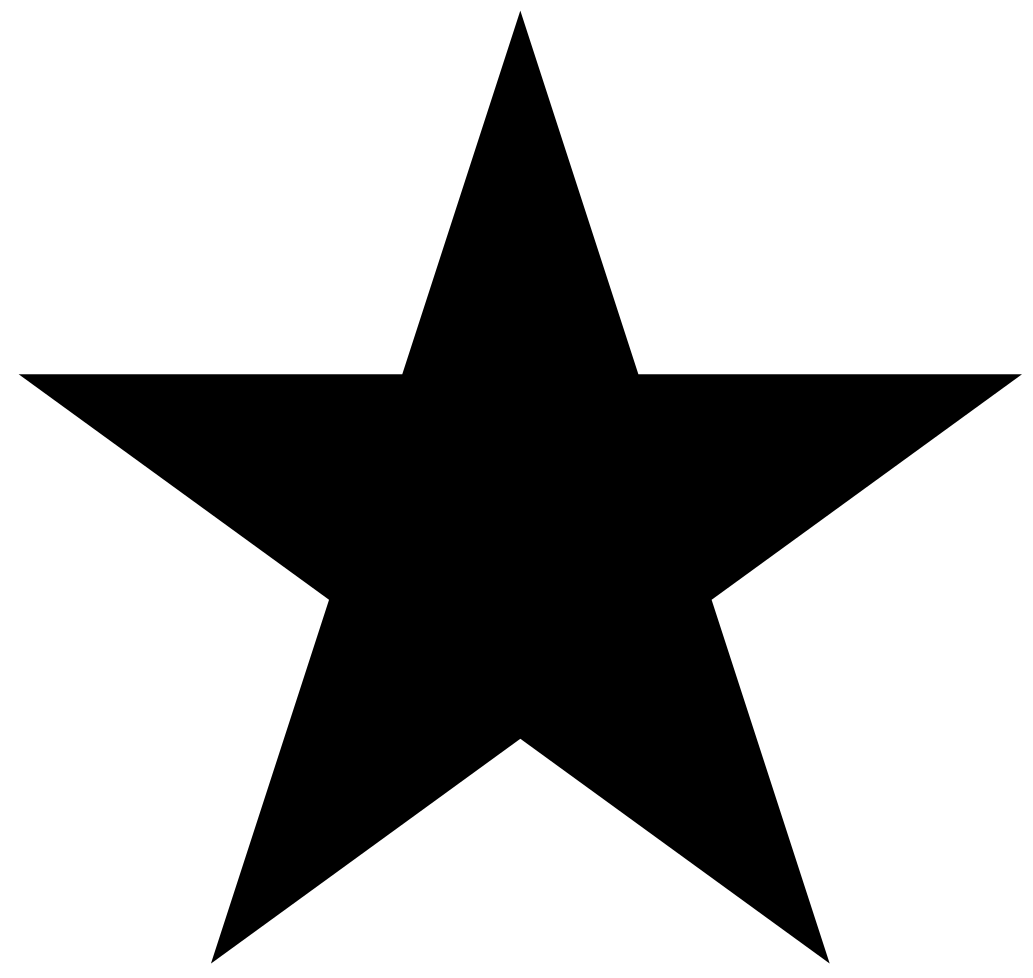 **7** |
| Avila et al. (1996) 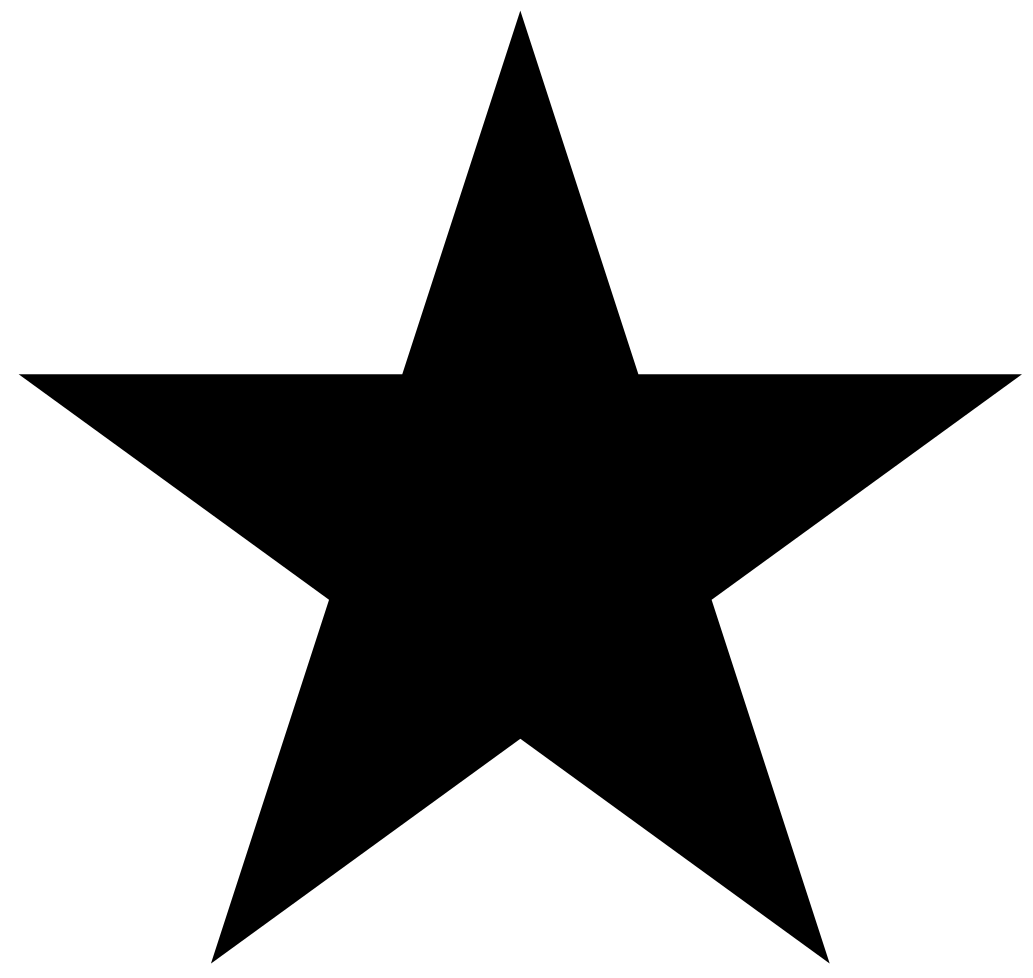 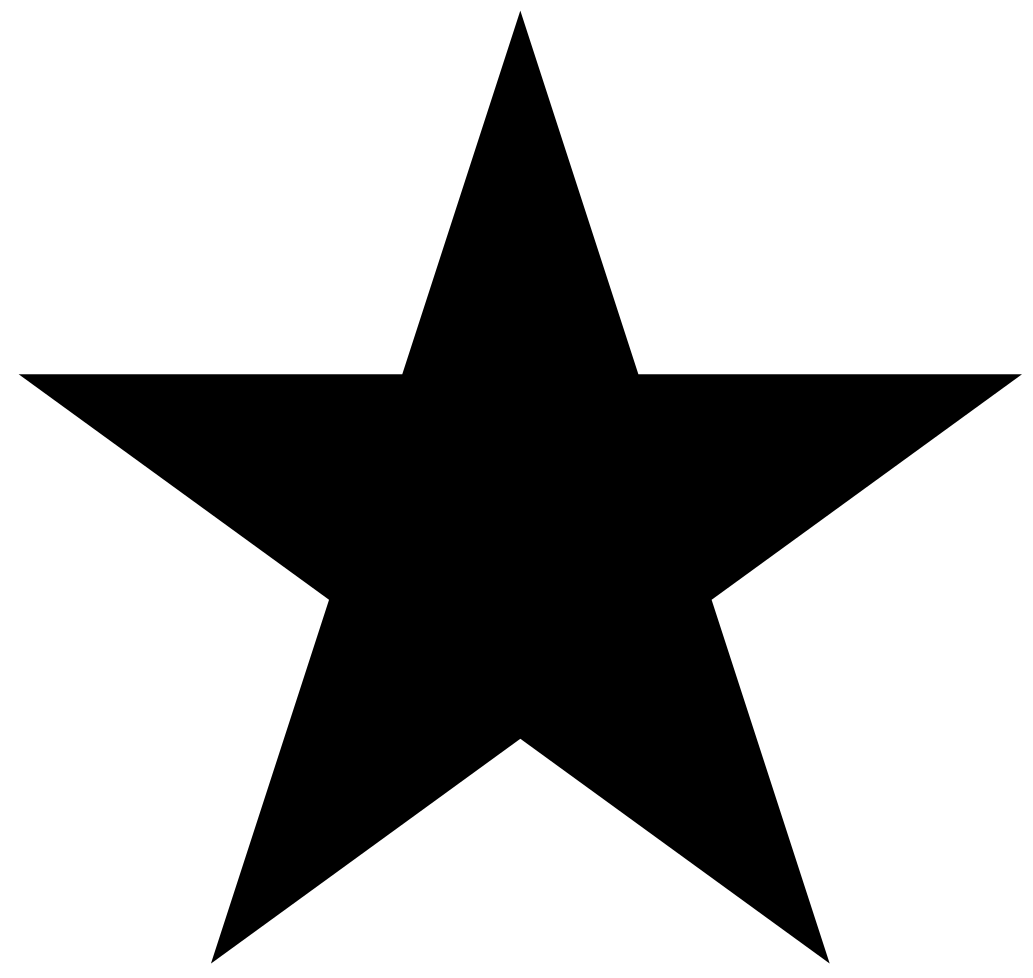 | 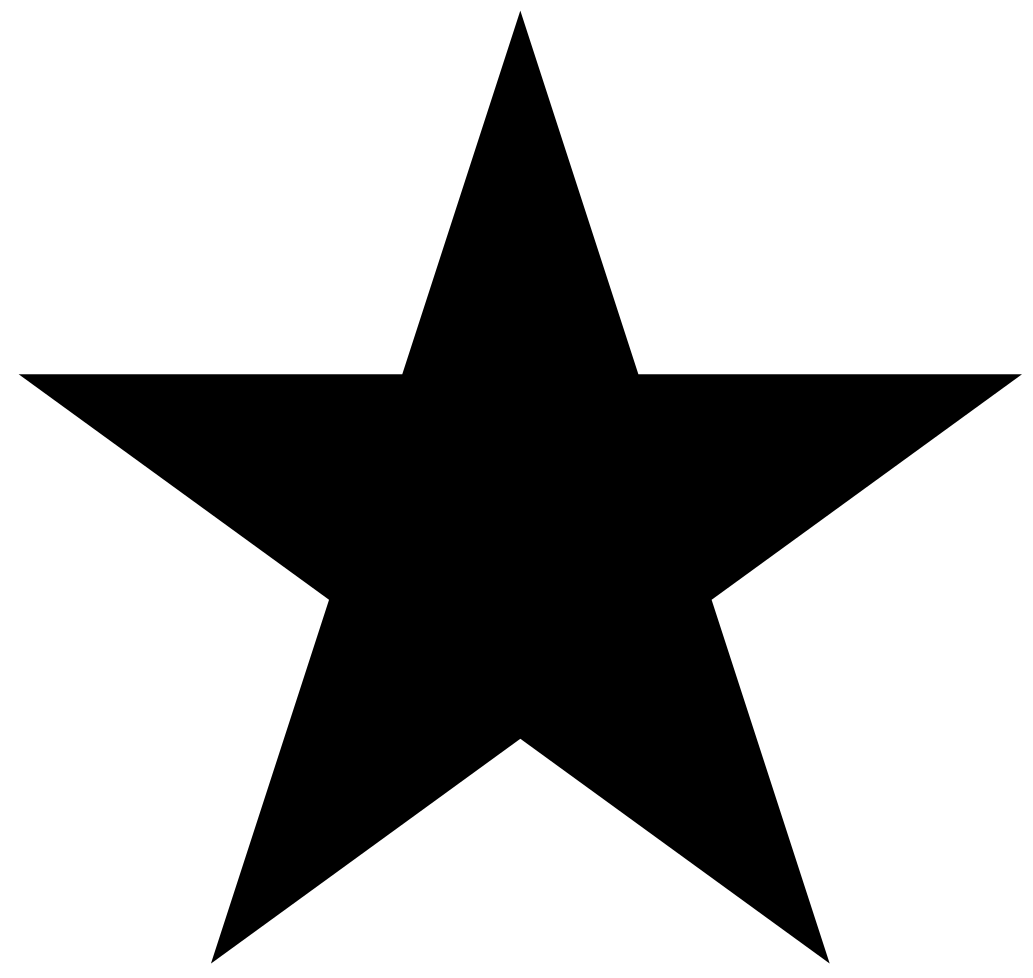 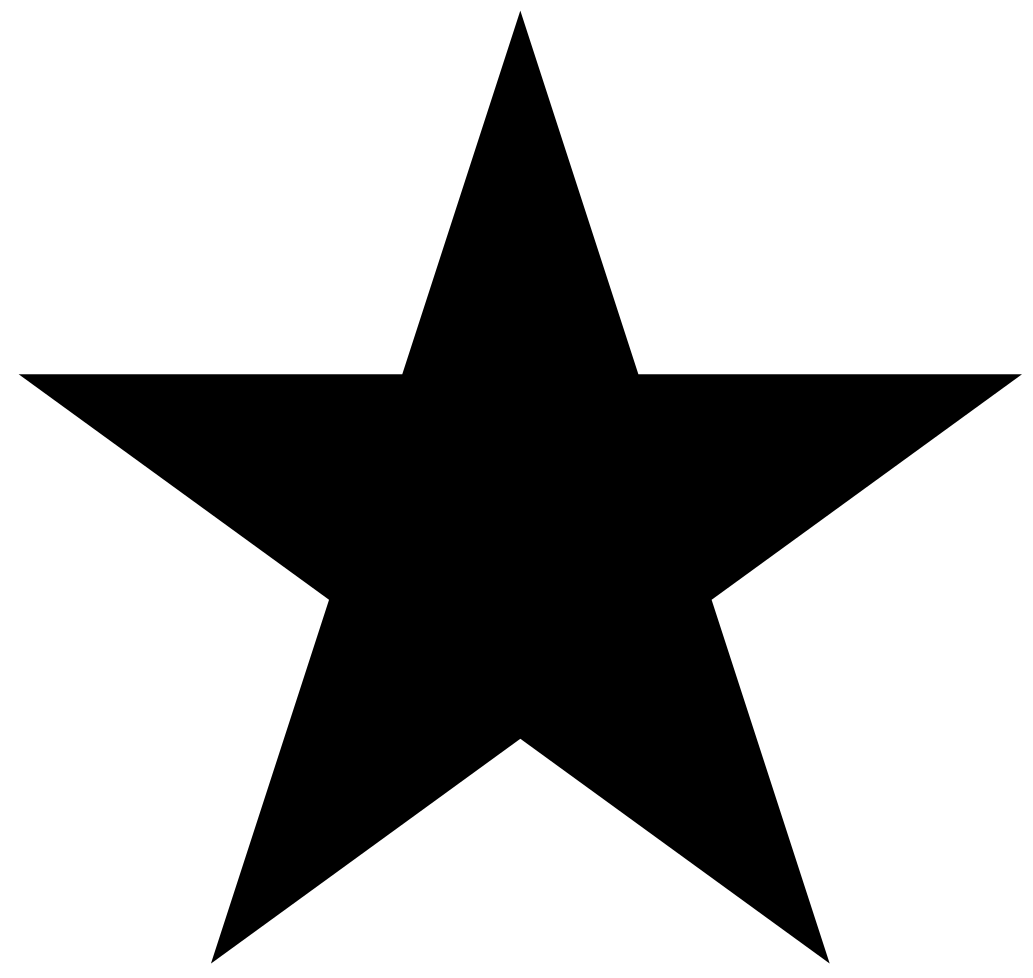 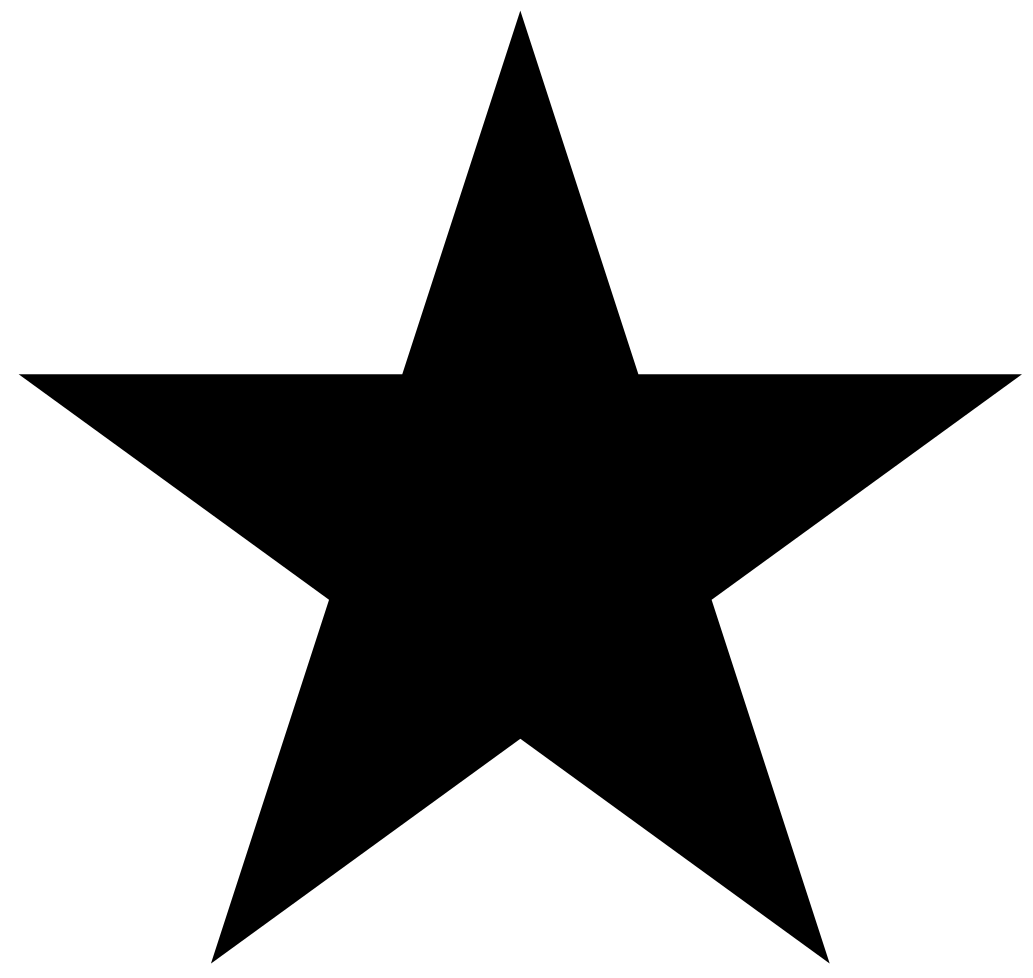 **5** |
| Yildirim et al. (1997) 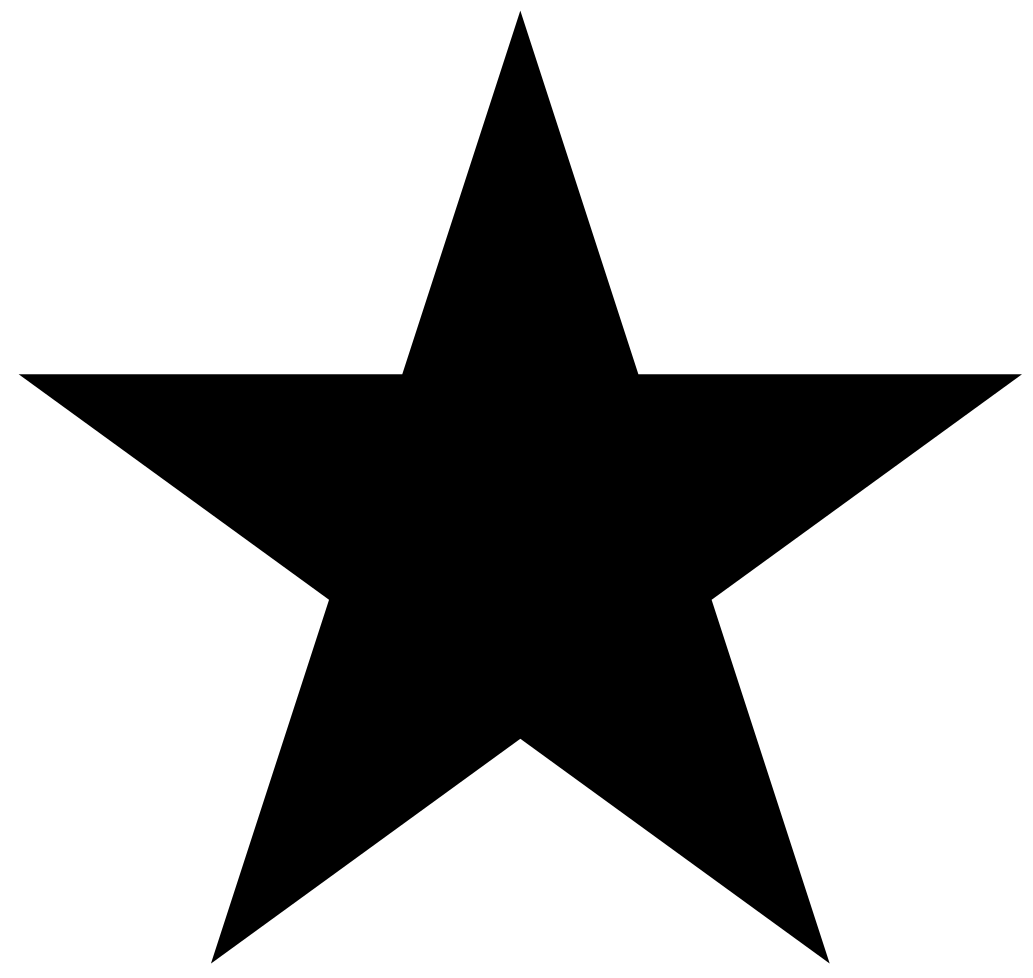 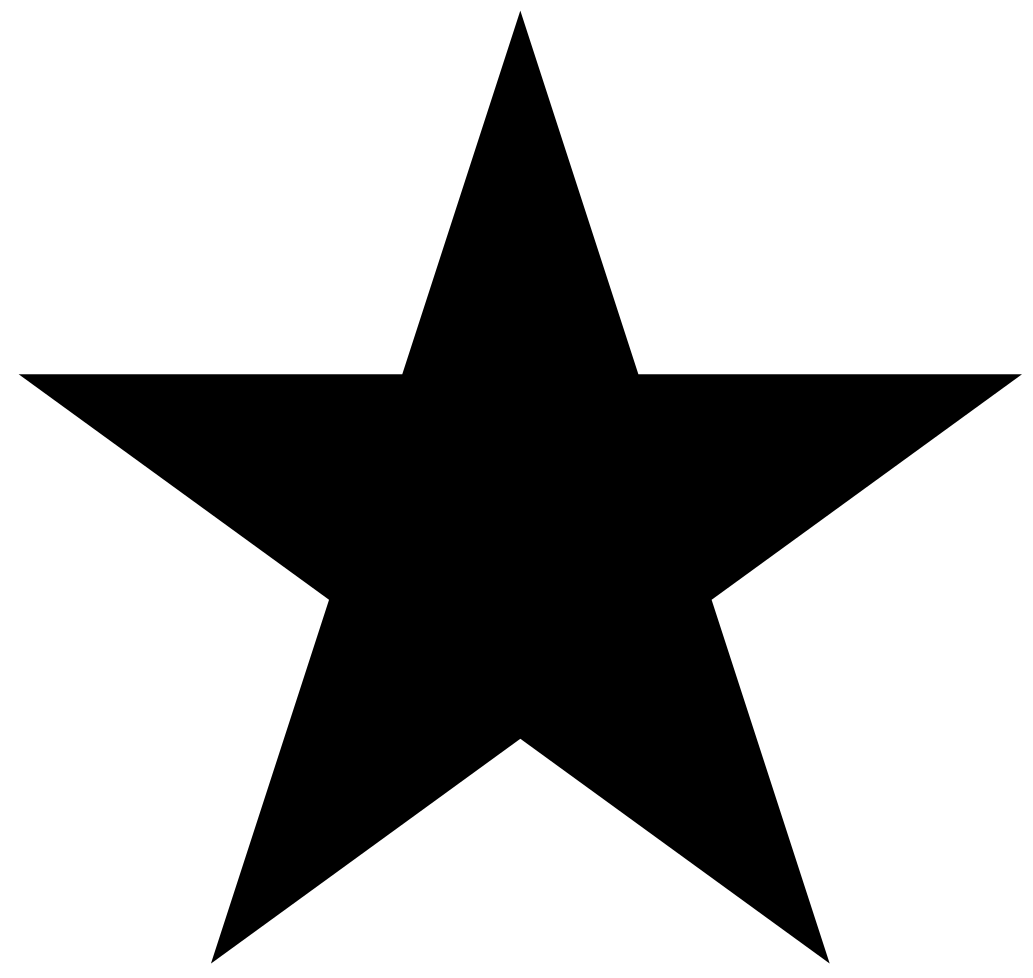 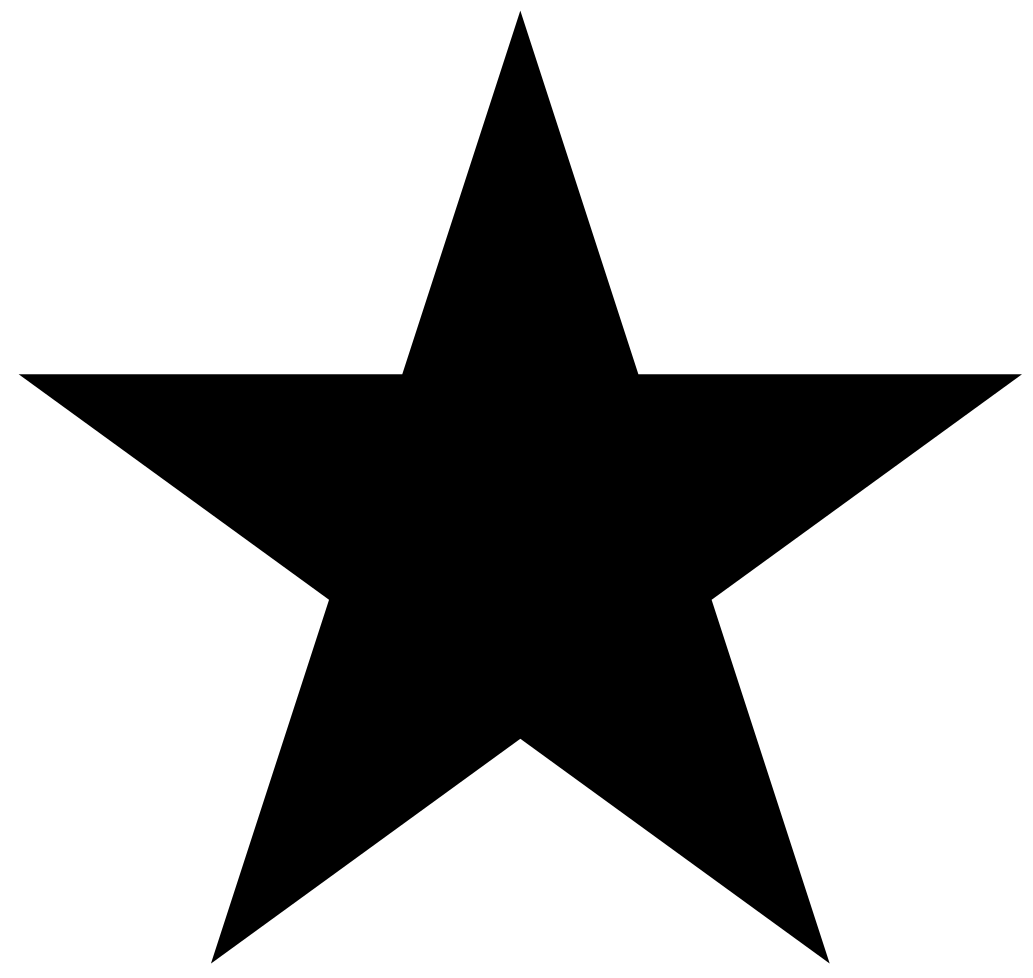 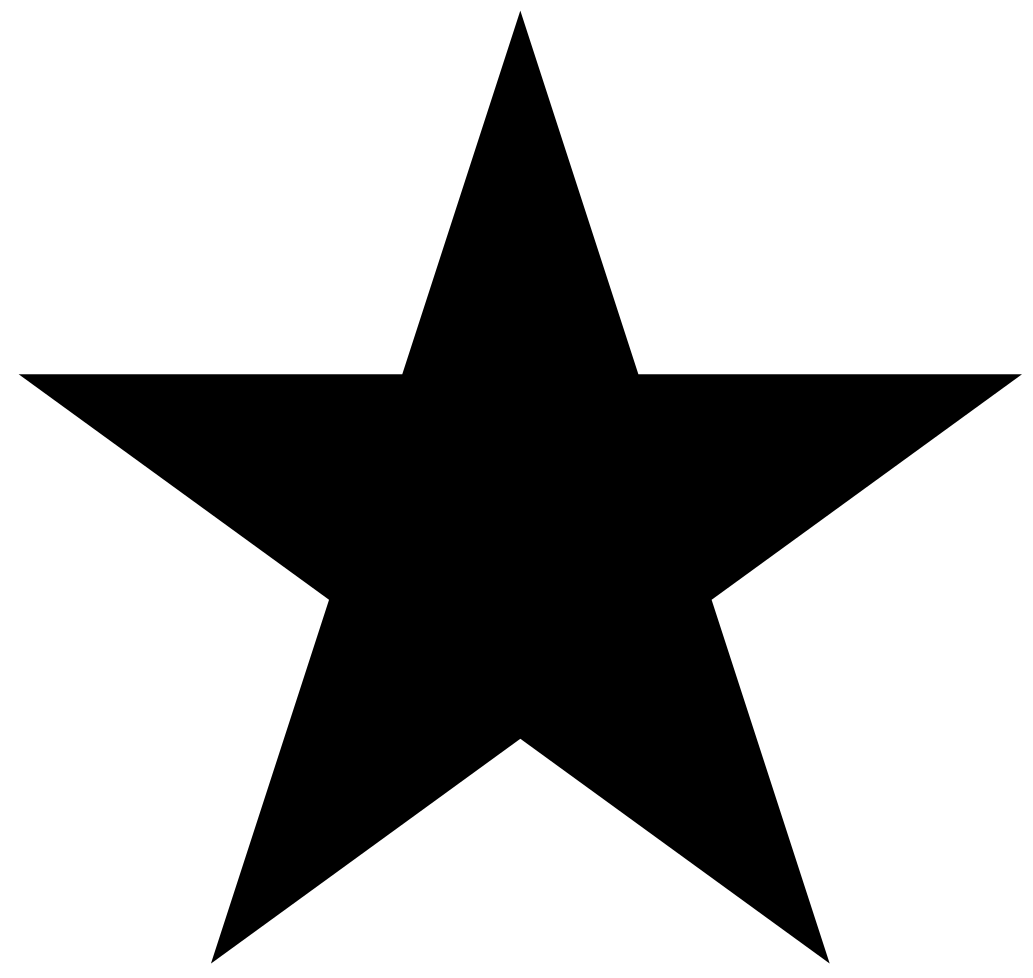 | 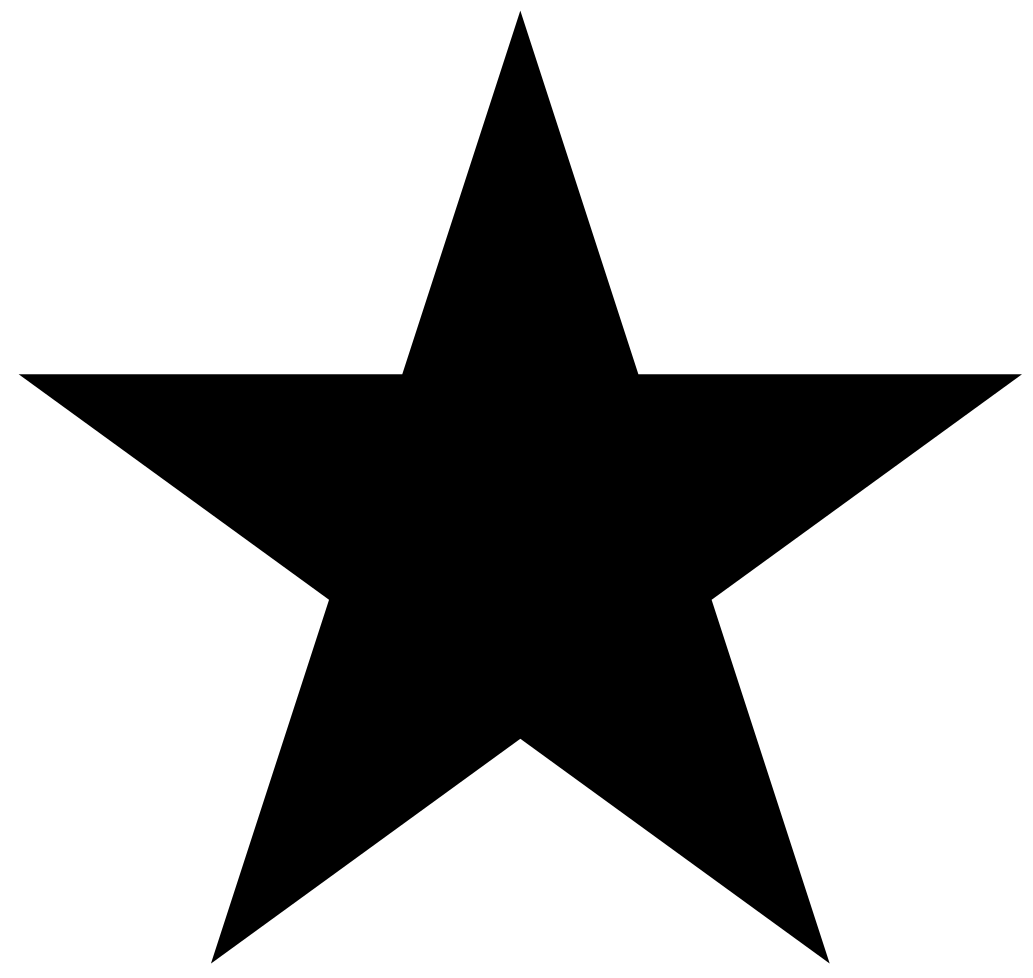 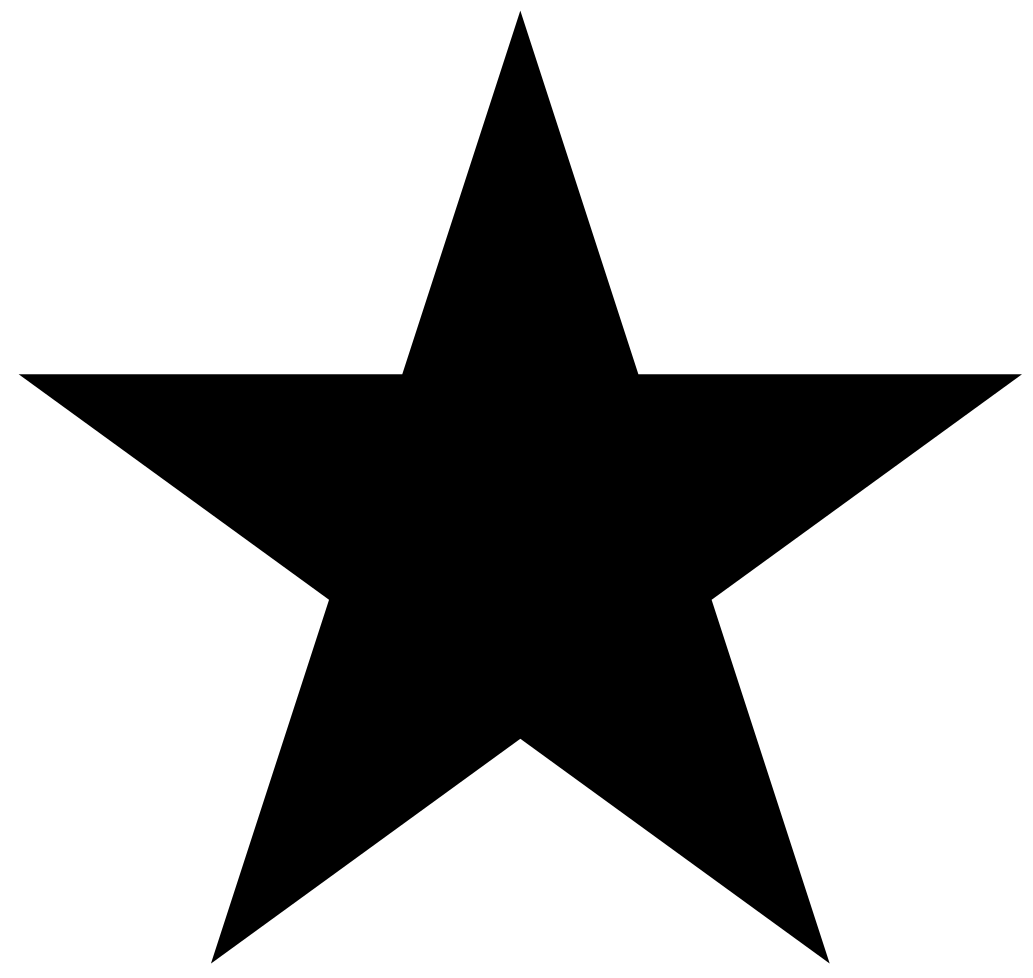 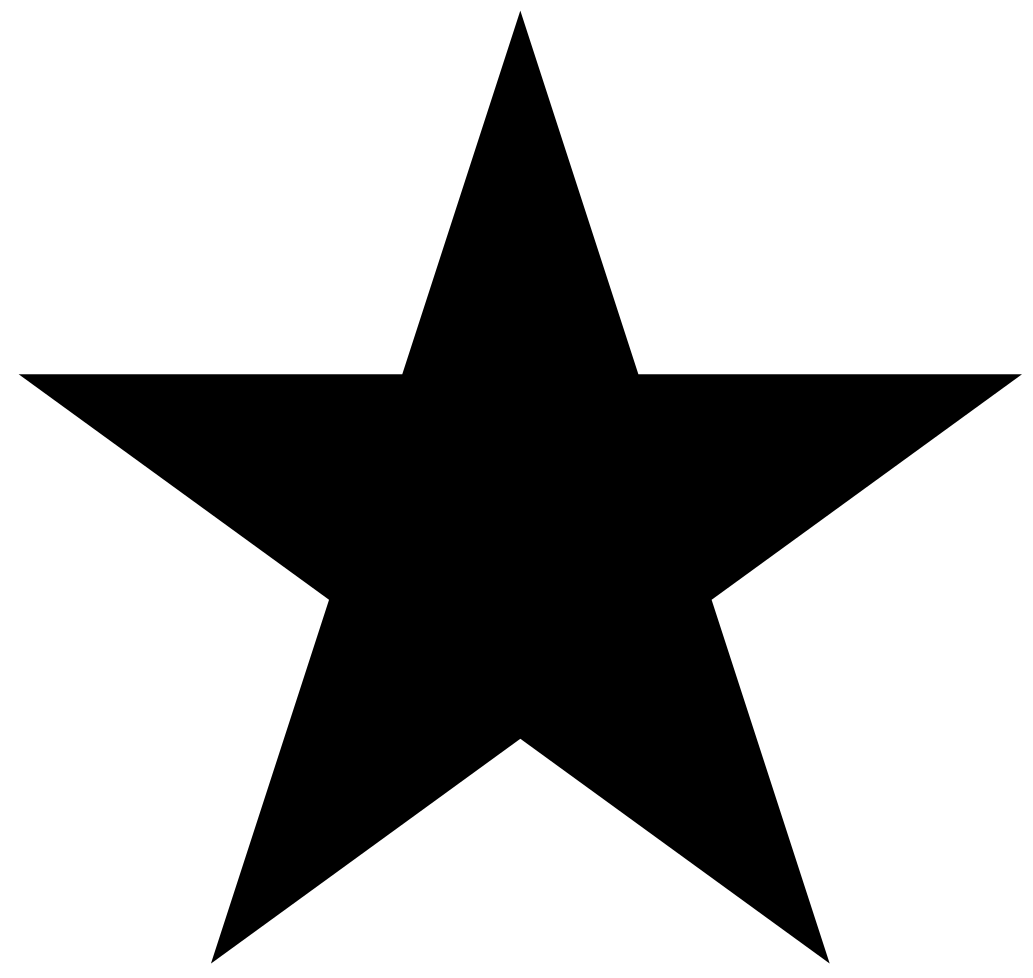 **8** |
| Hameed et al. (2001) 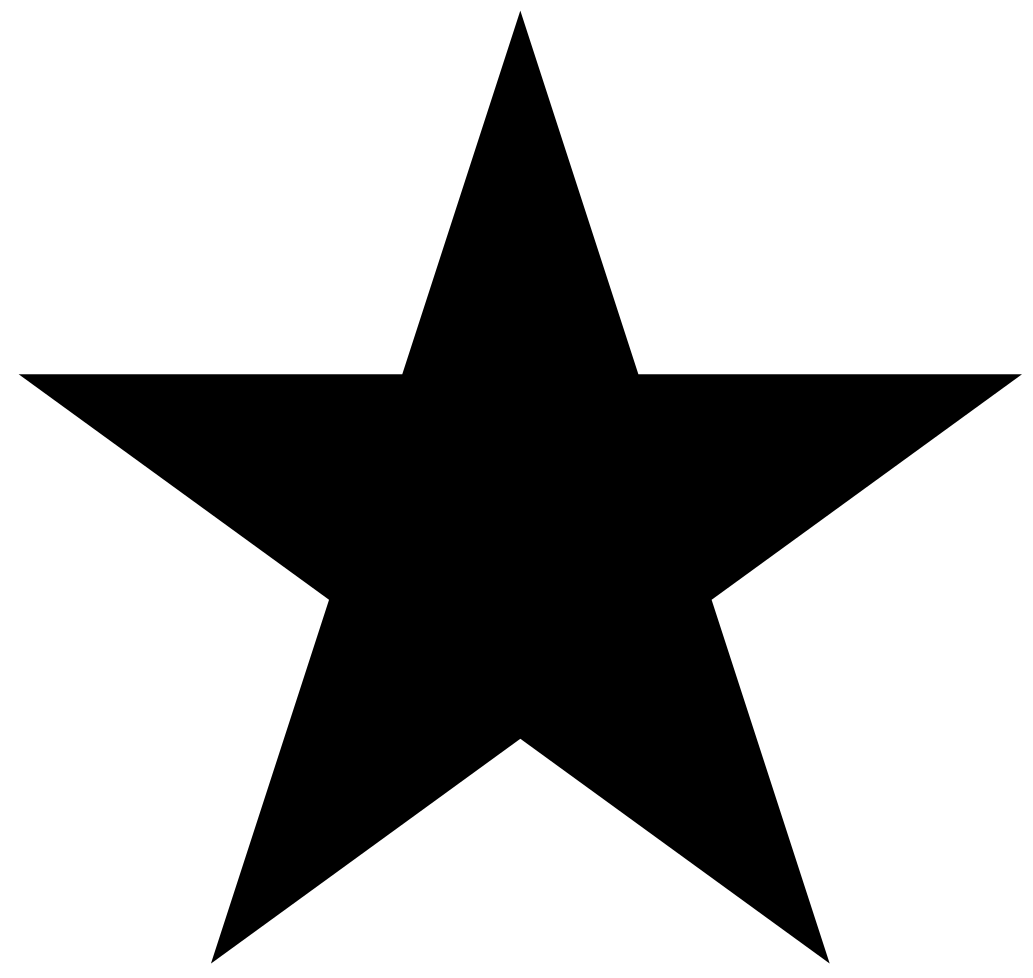 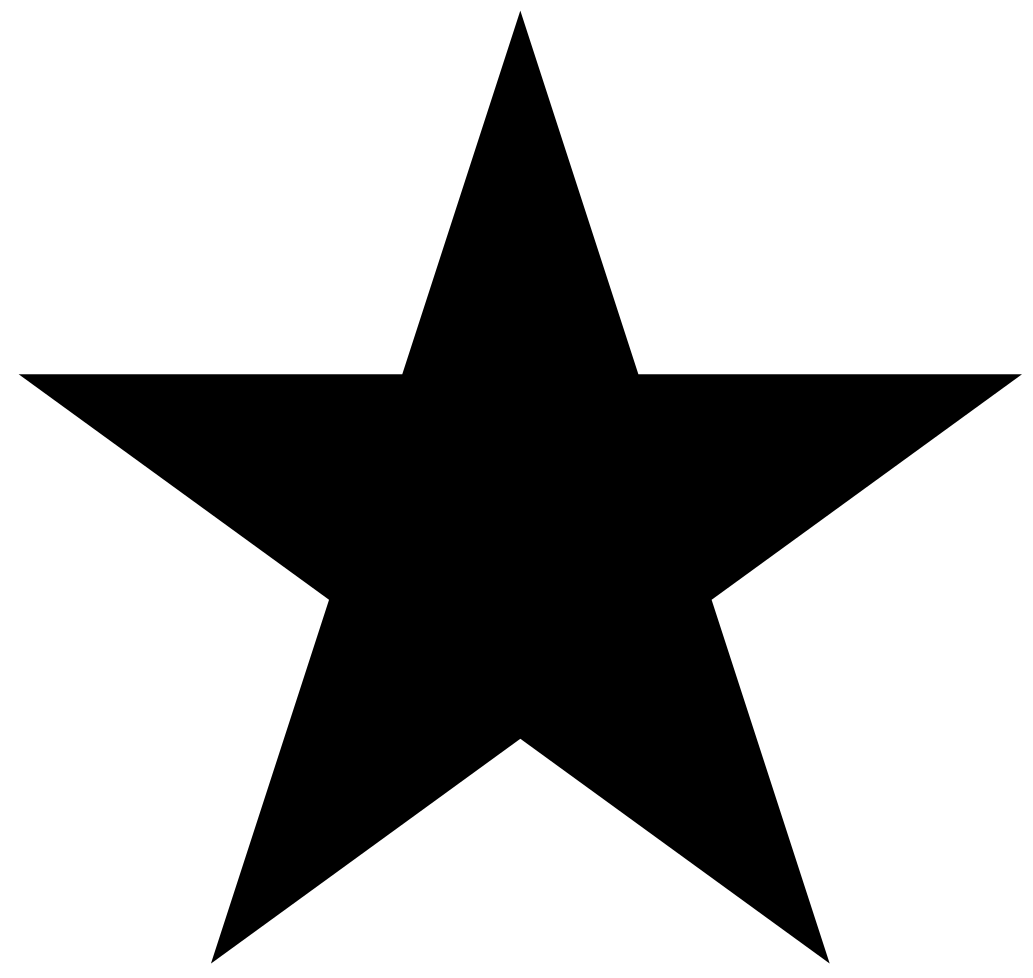 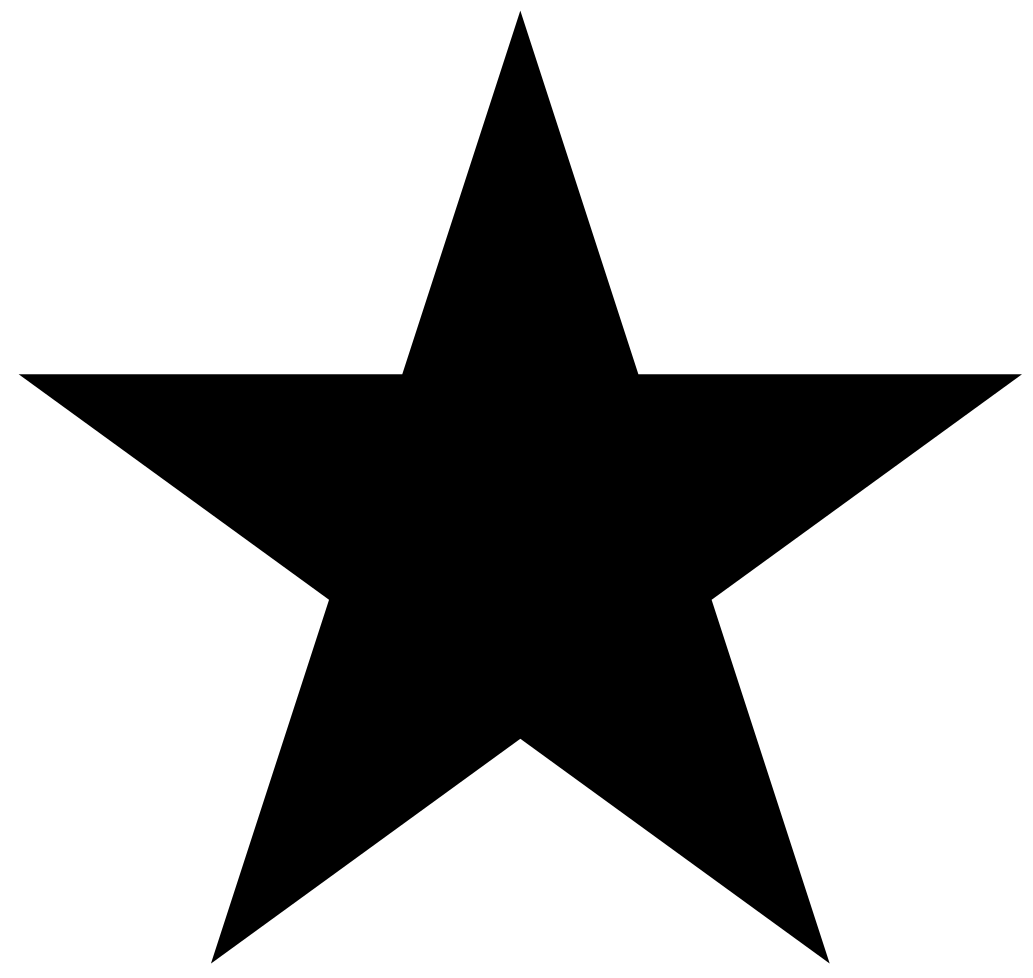 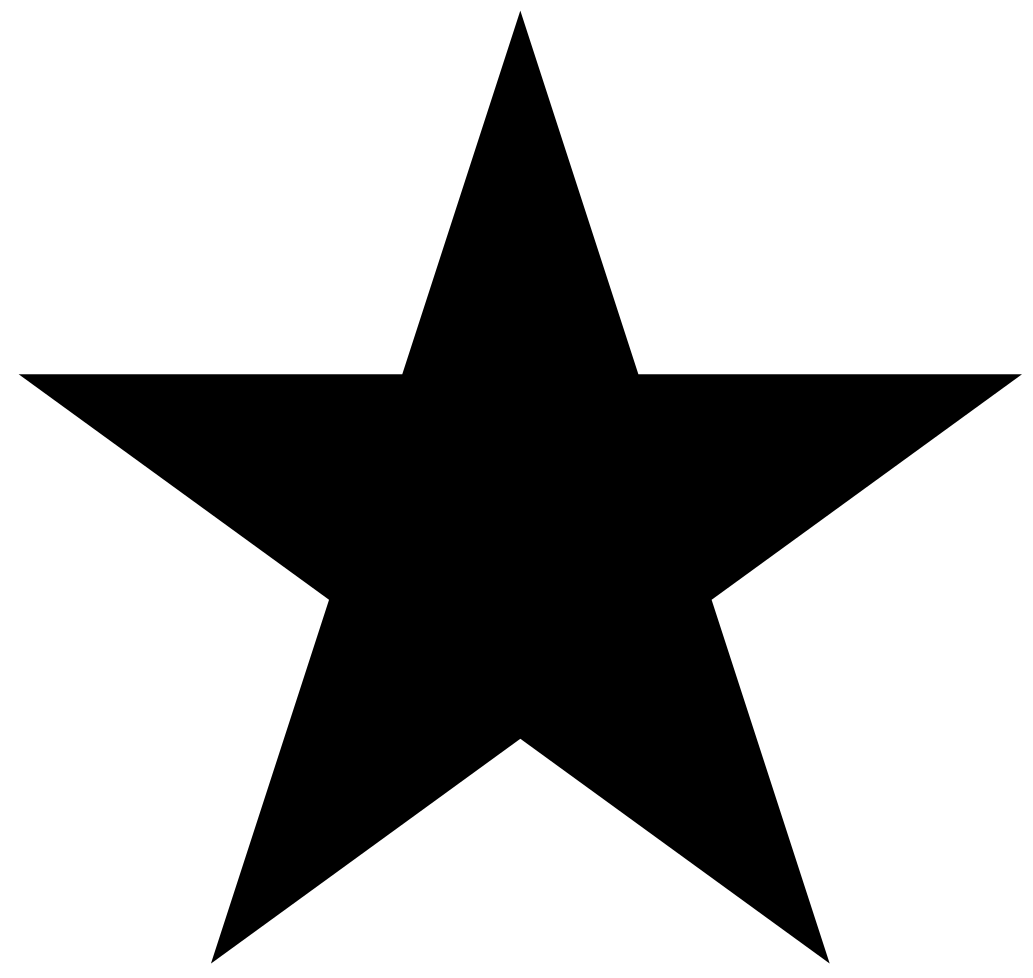 | 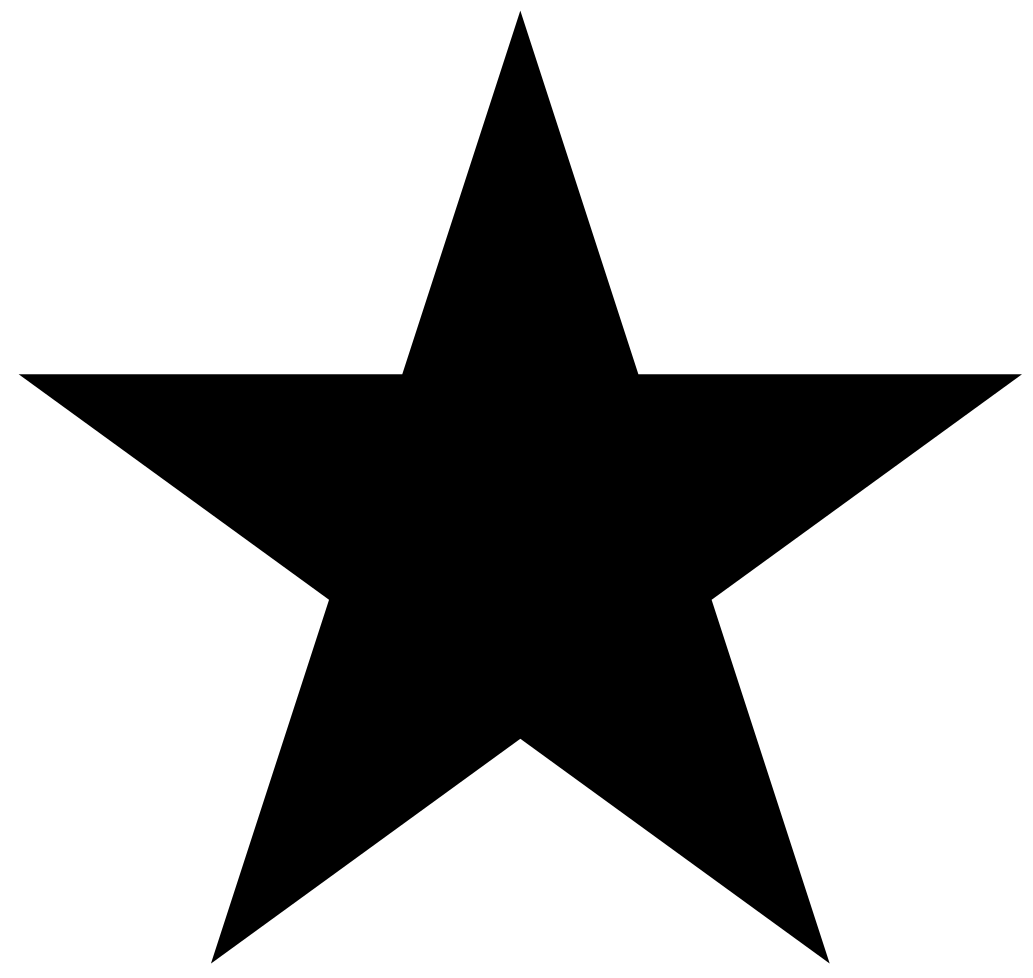 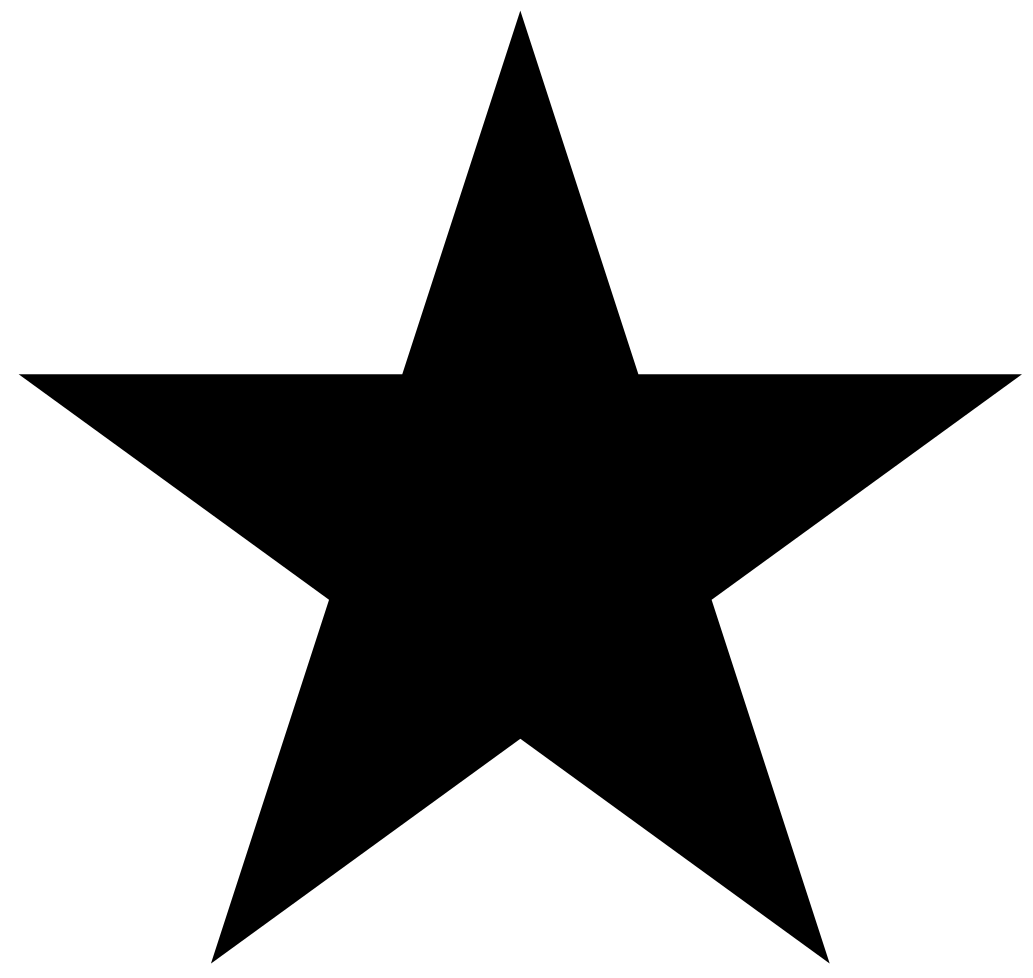 **6** |
| Ortayli et al. (2001) 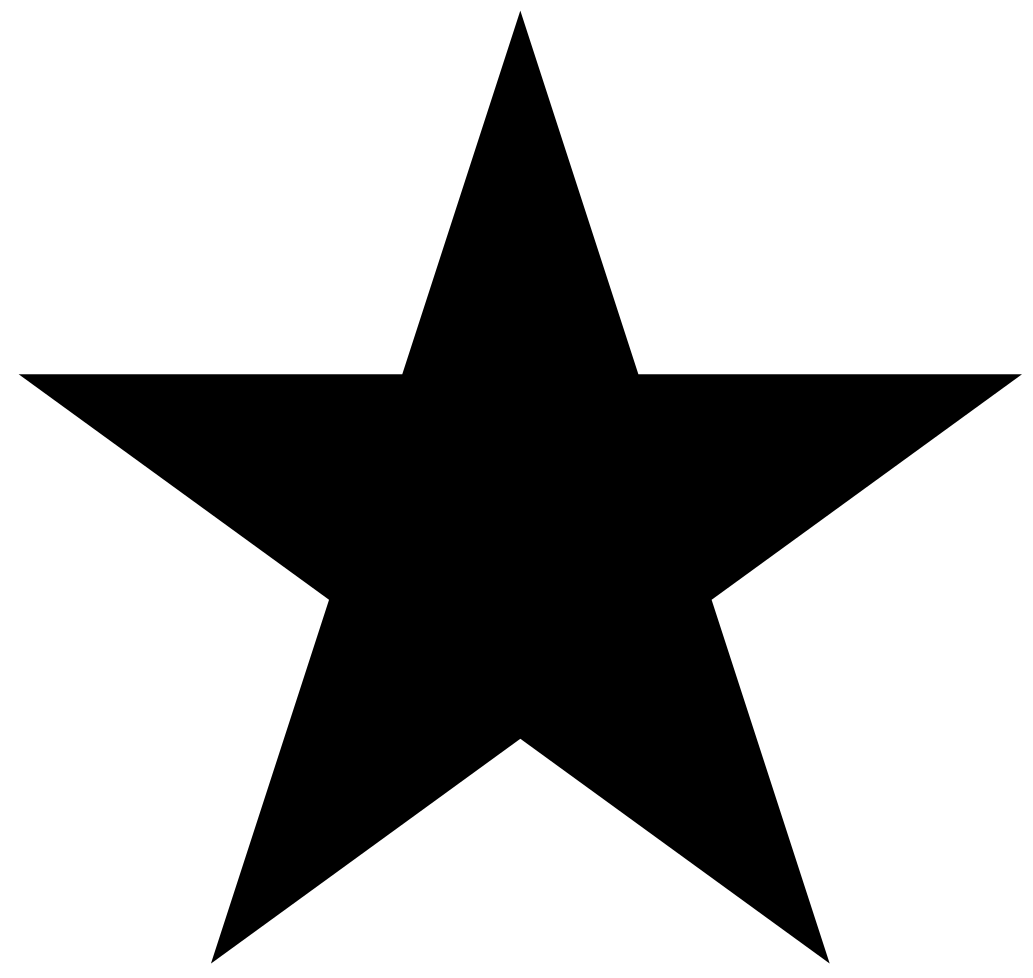 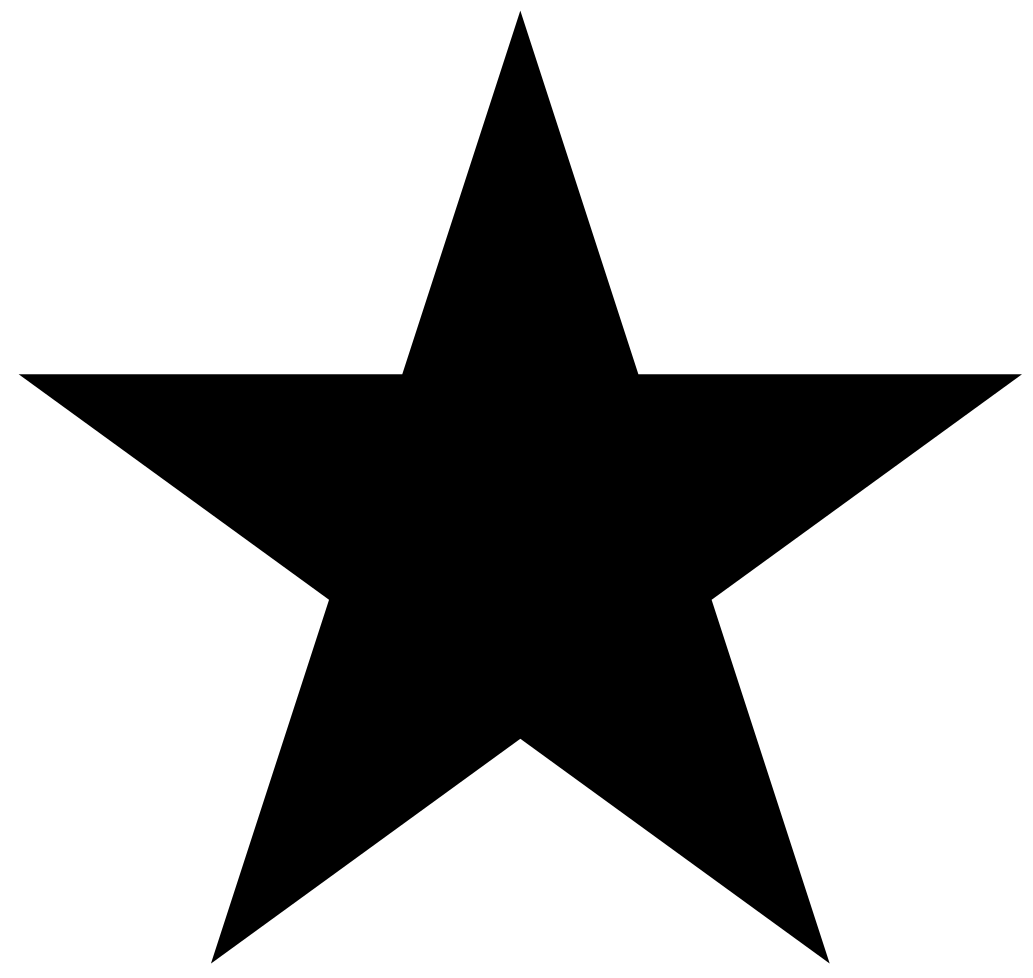 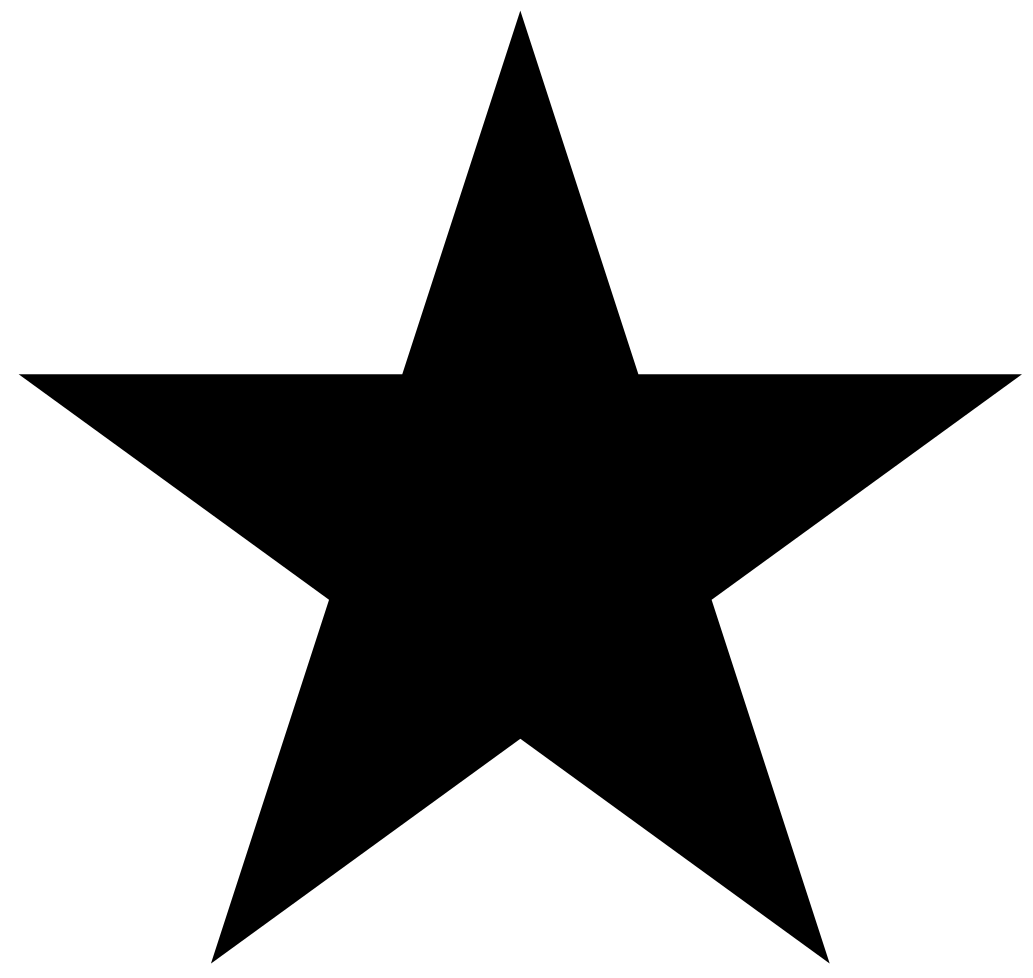 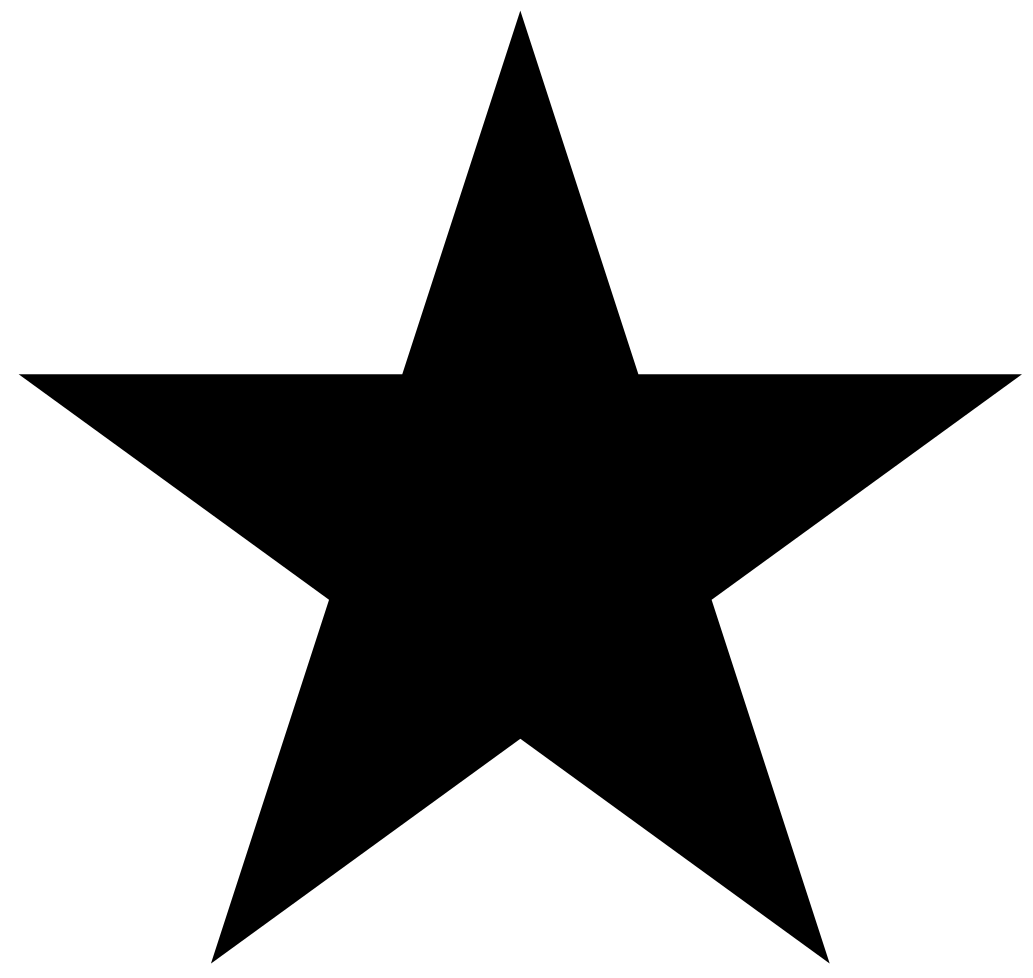 | 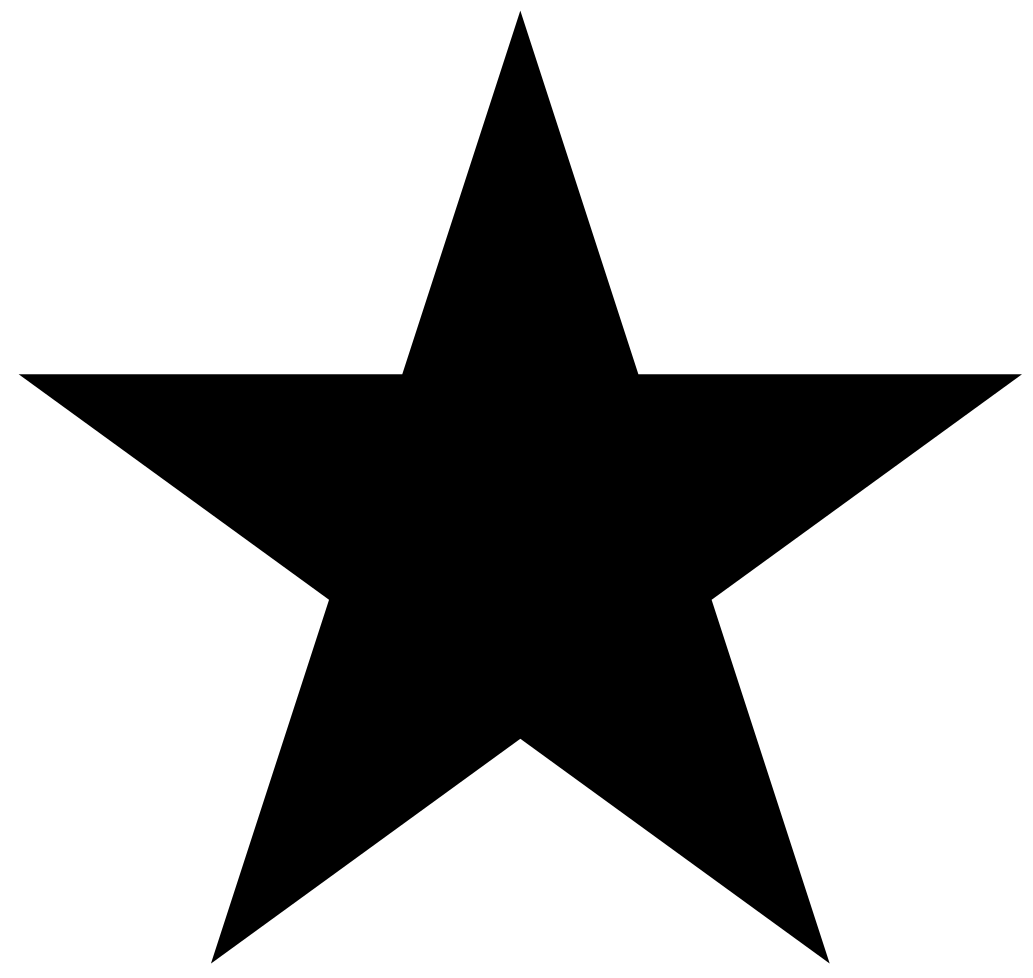 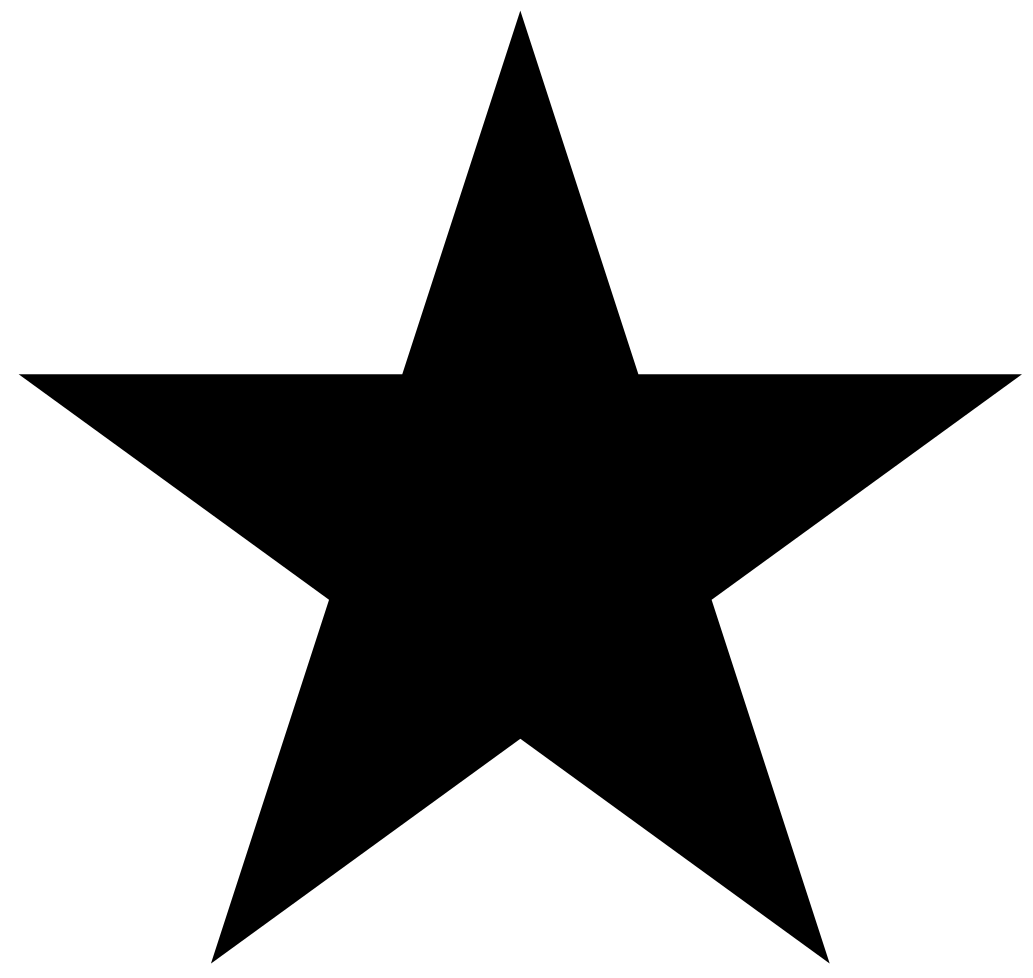 **6** |
| Xiang et al. (2007) 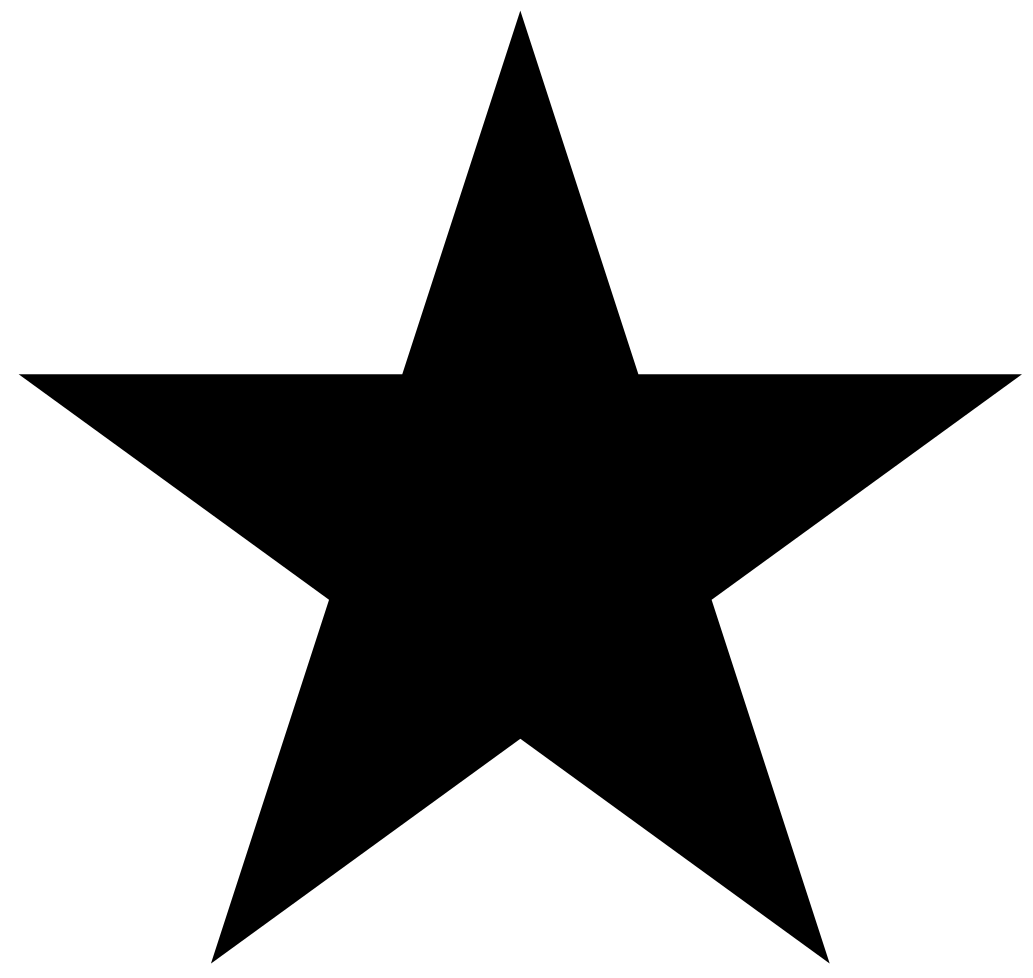 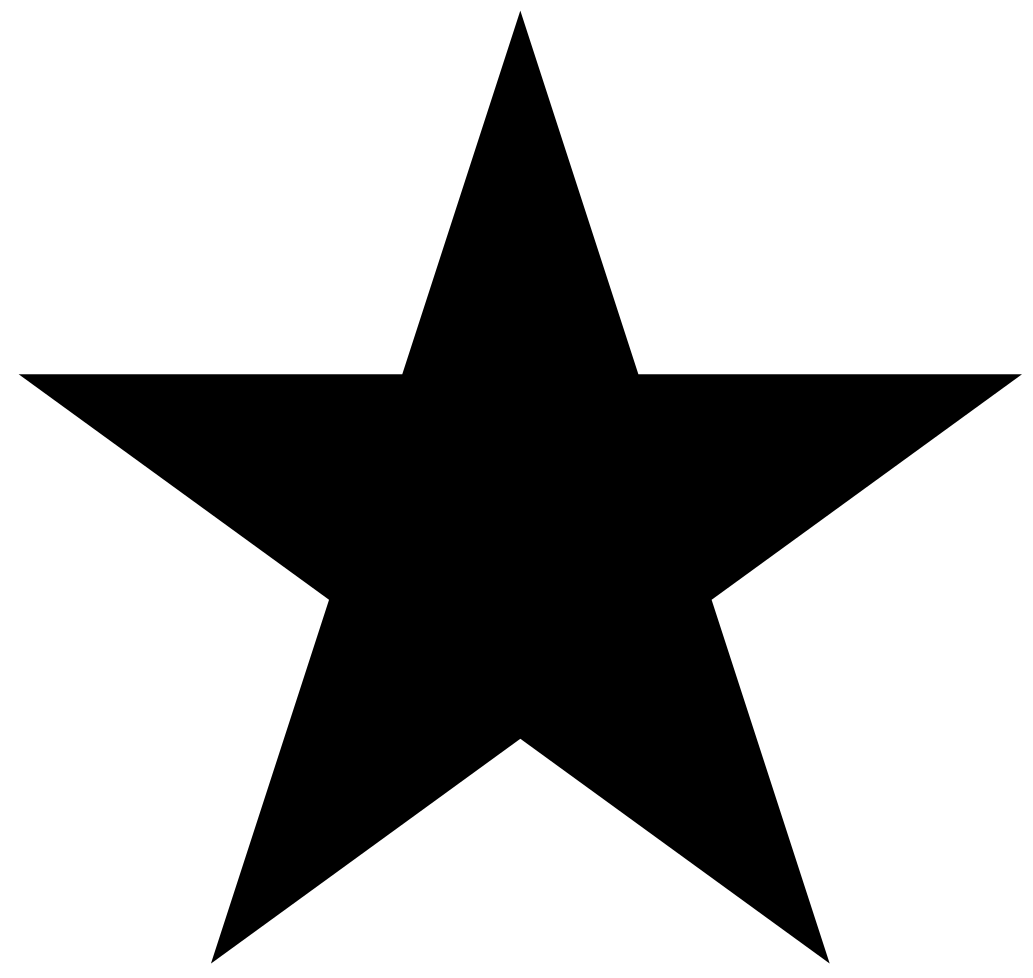 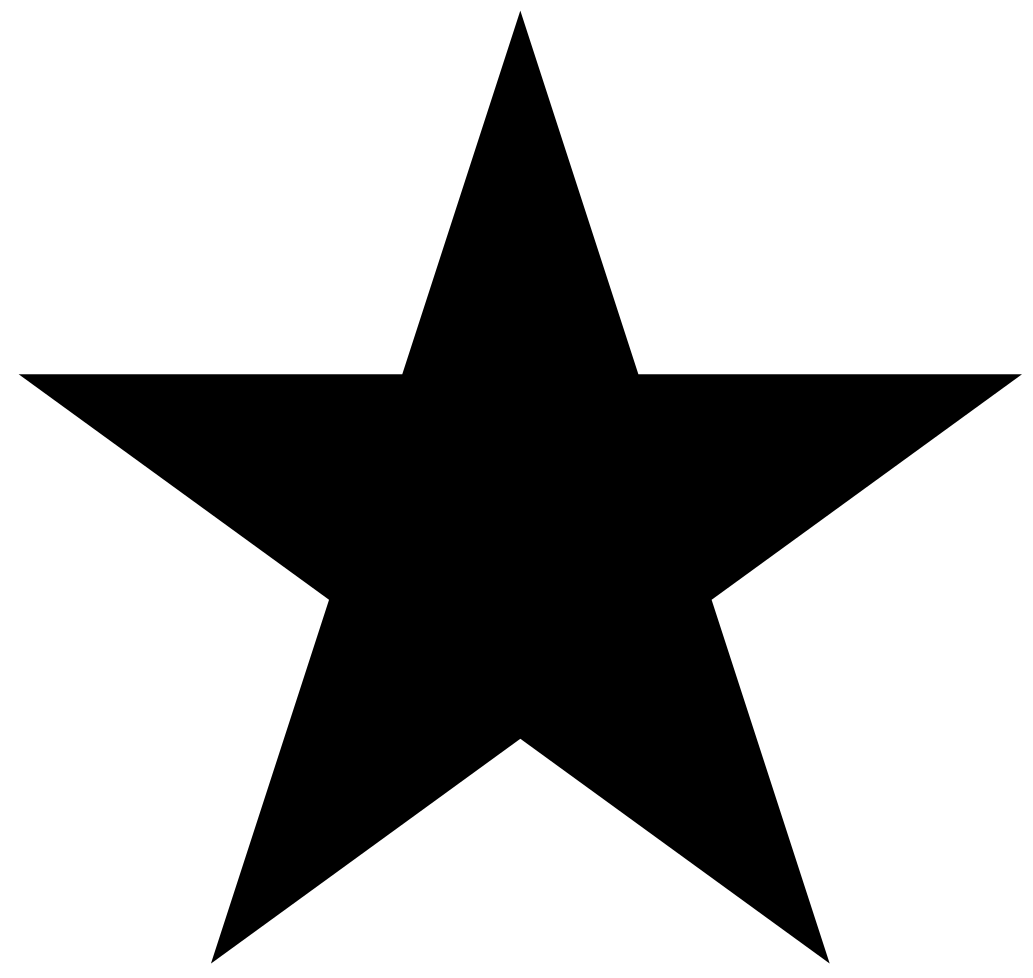 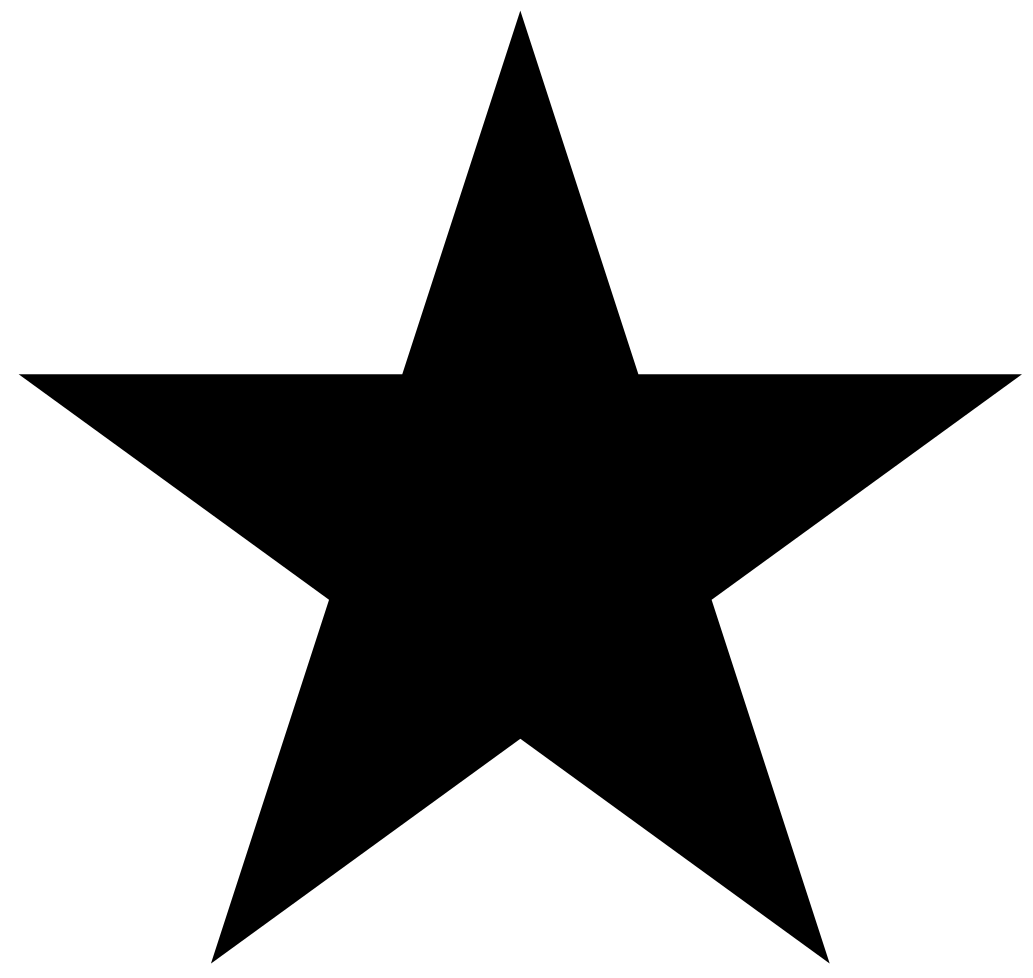 | 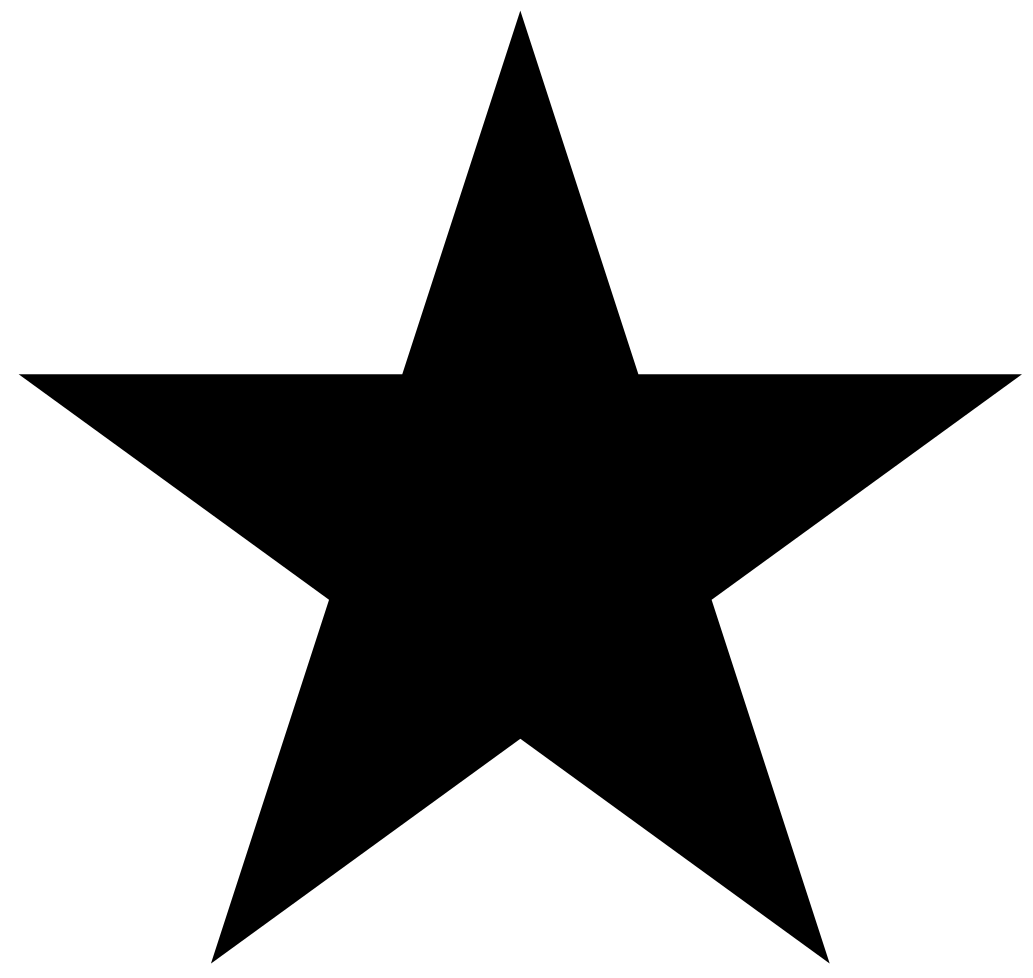 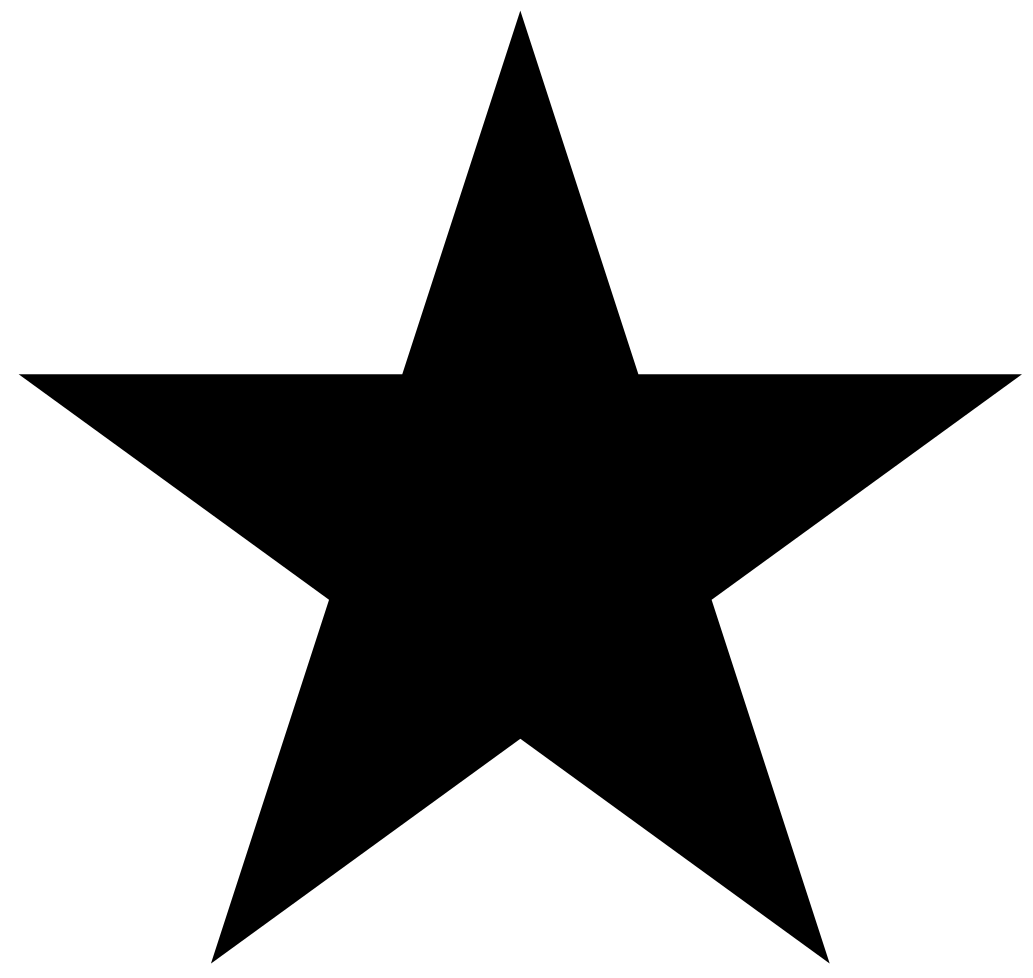 **7** |
| Barreiros et al. (2010) 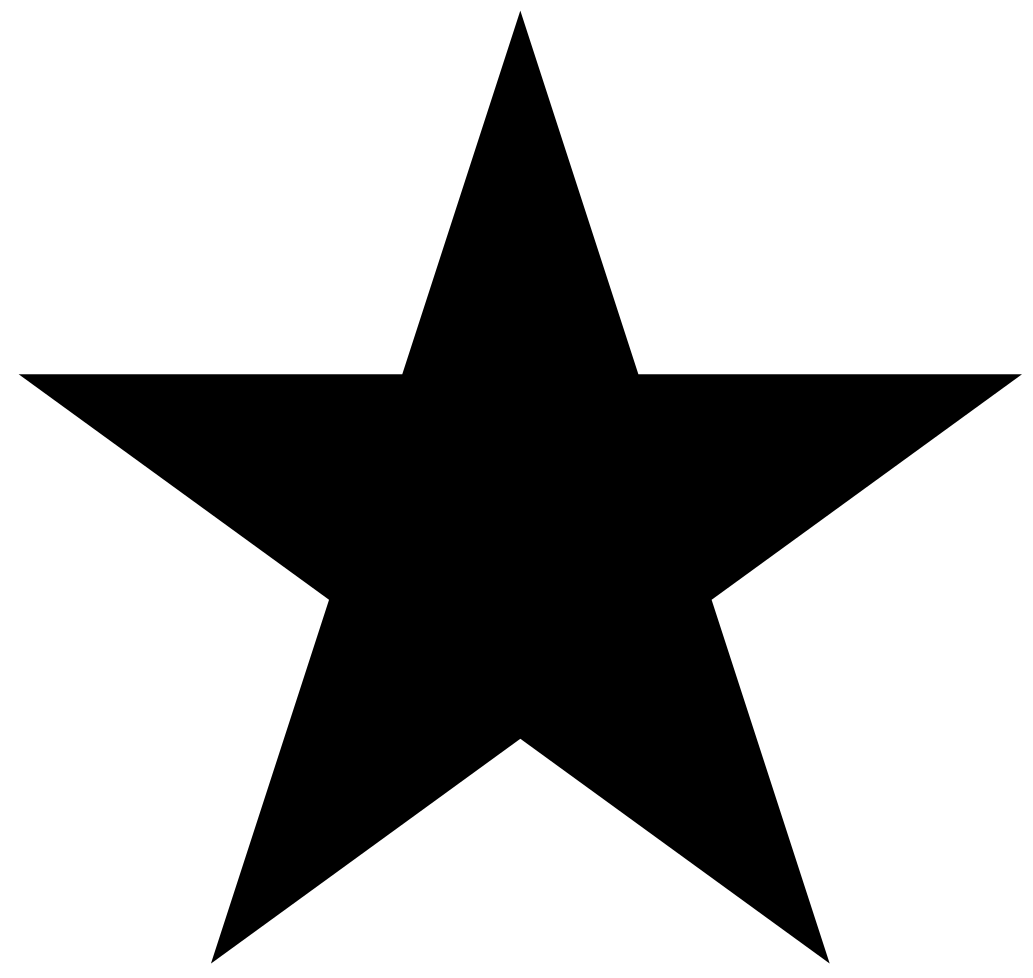 | 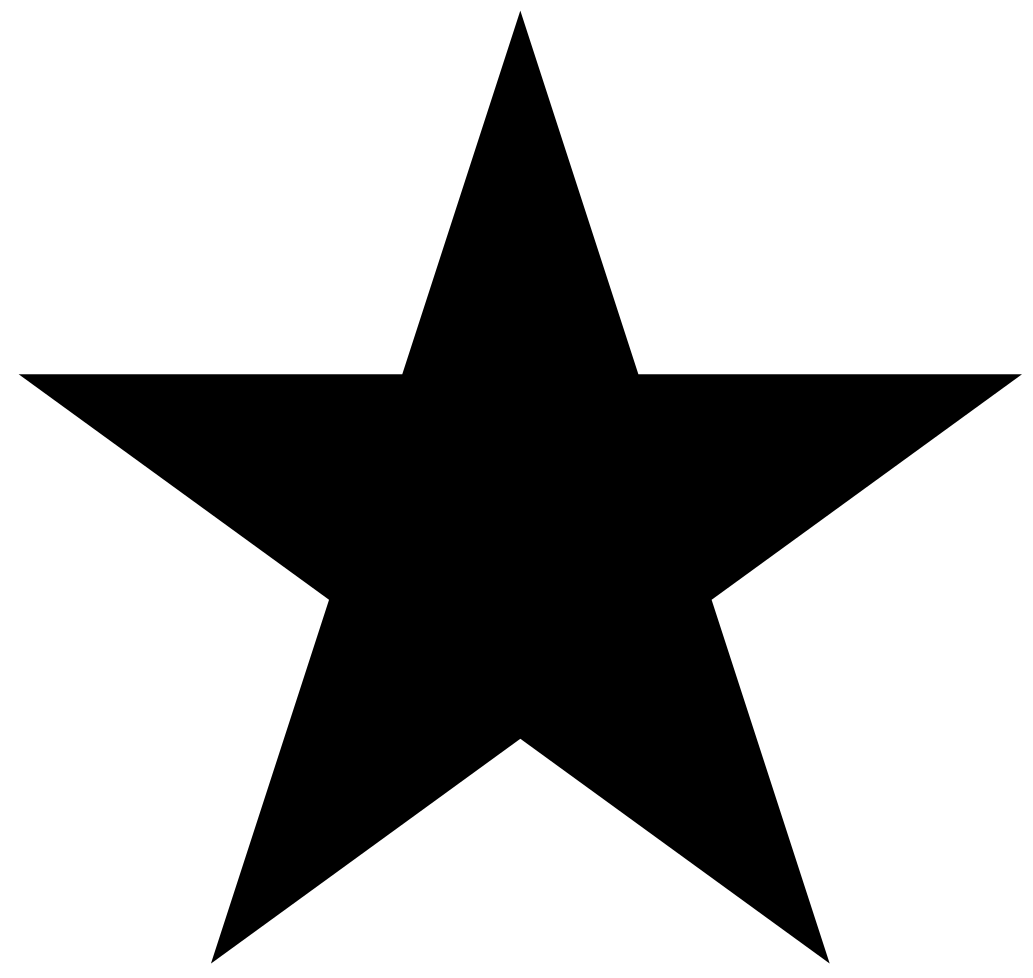 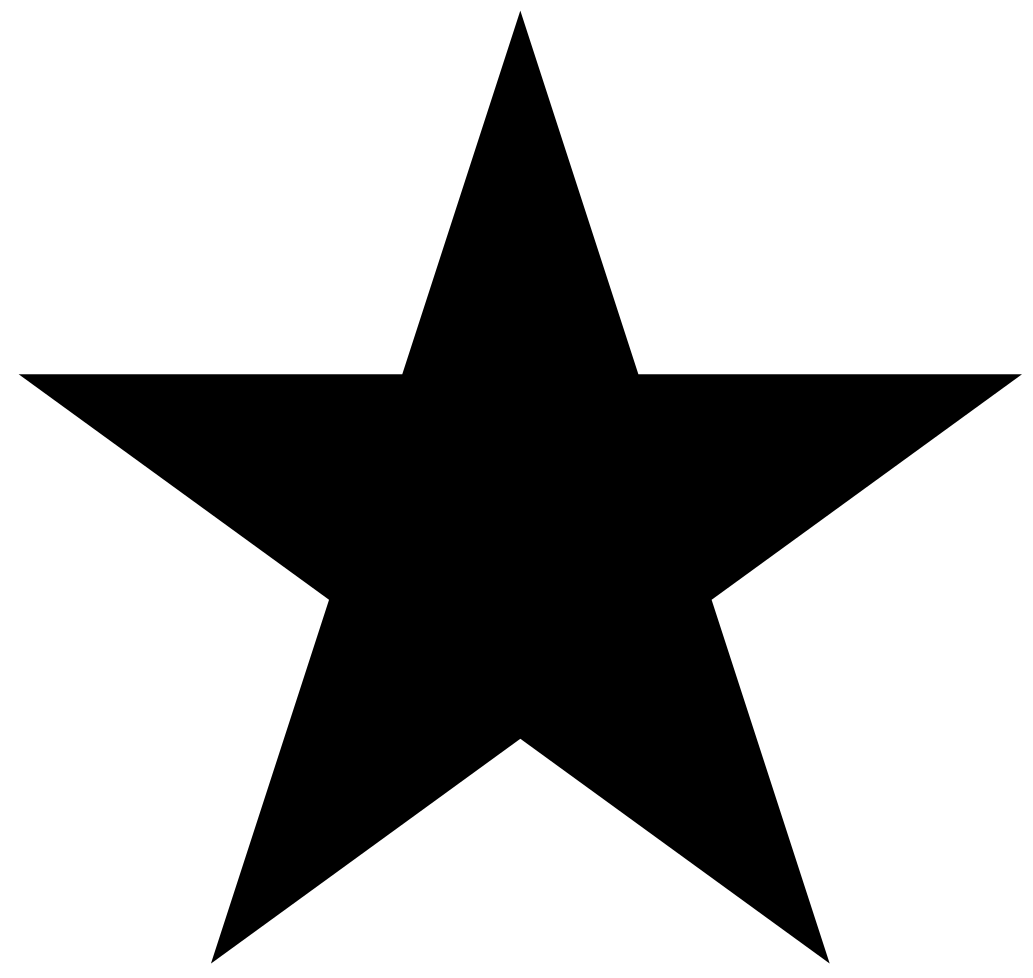 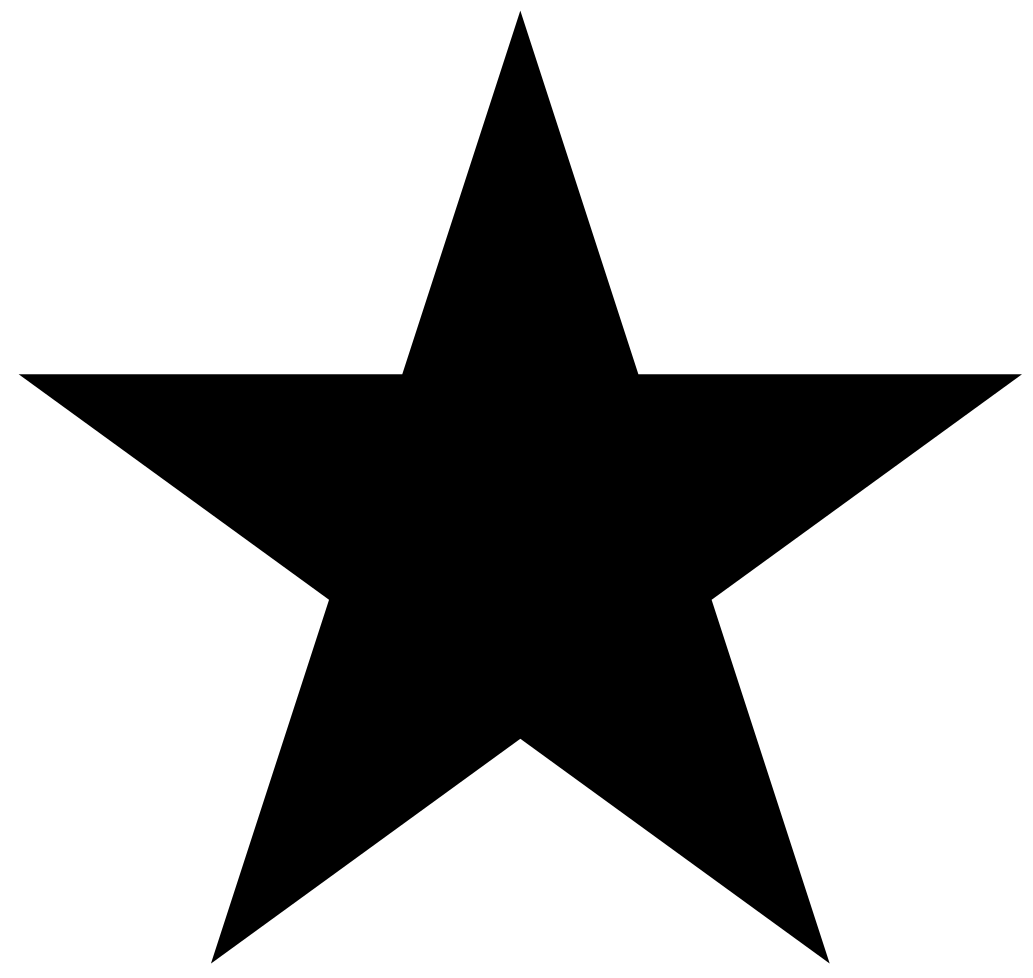 **4** |
| Bender et al. (2013) 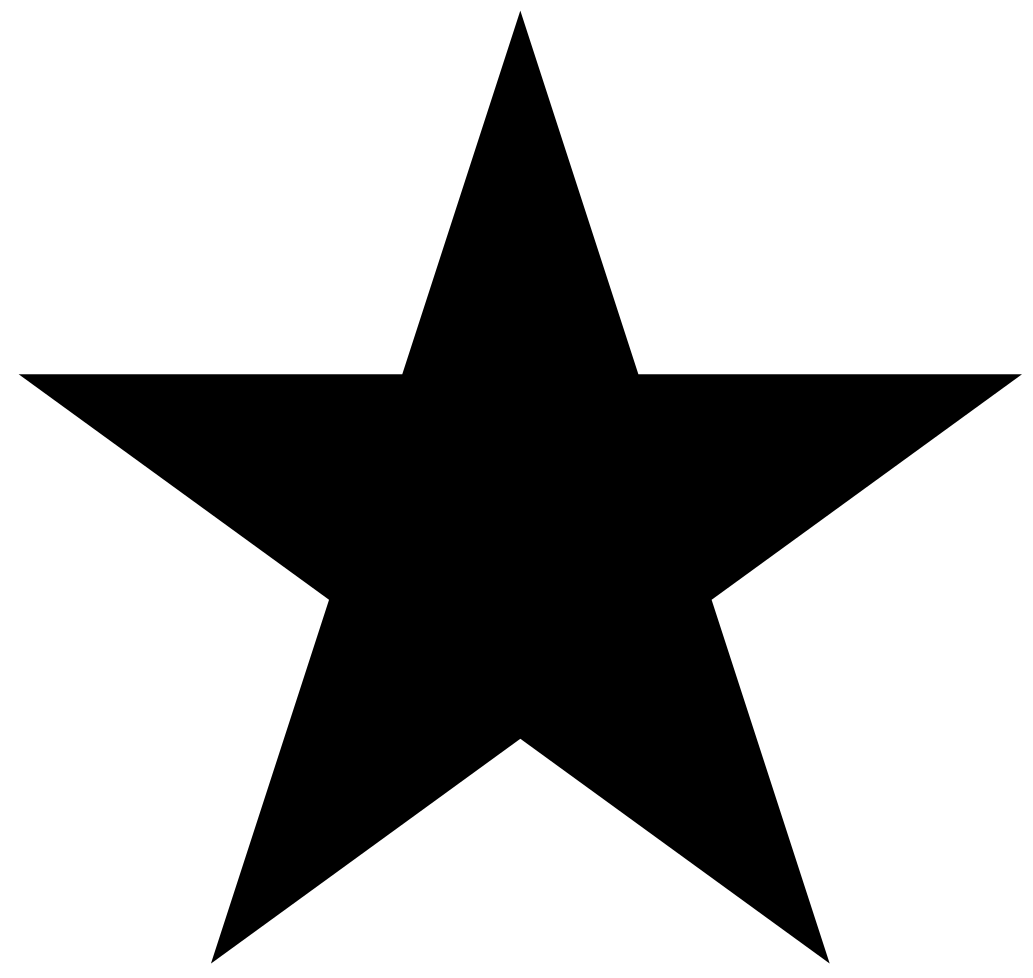 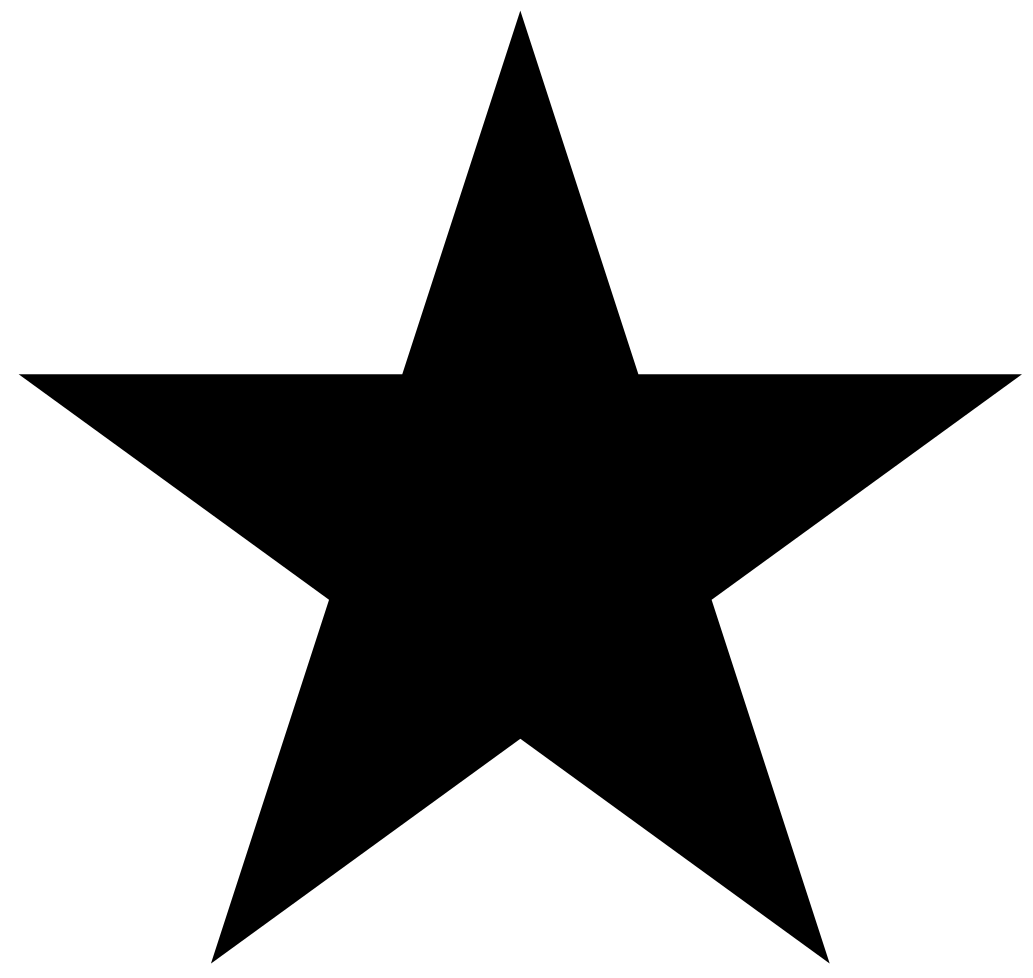 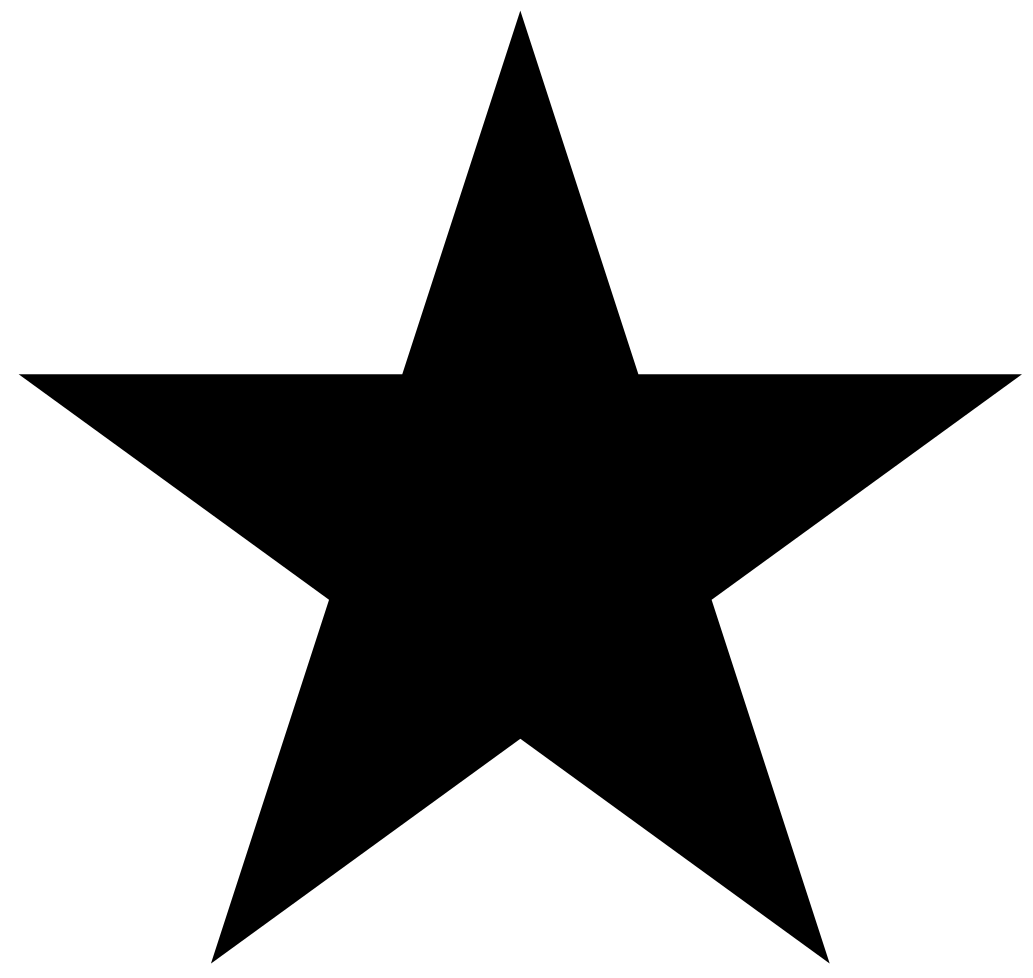 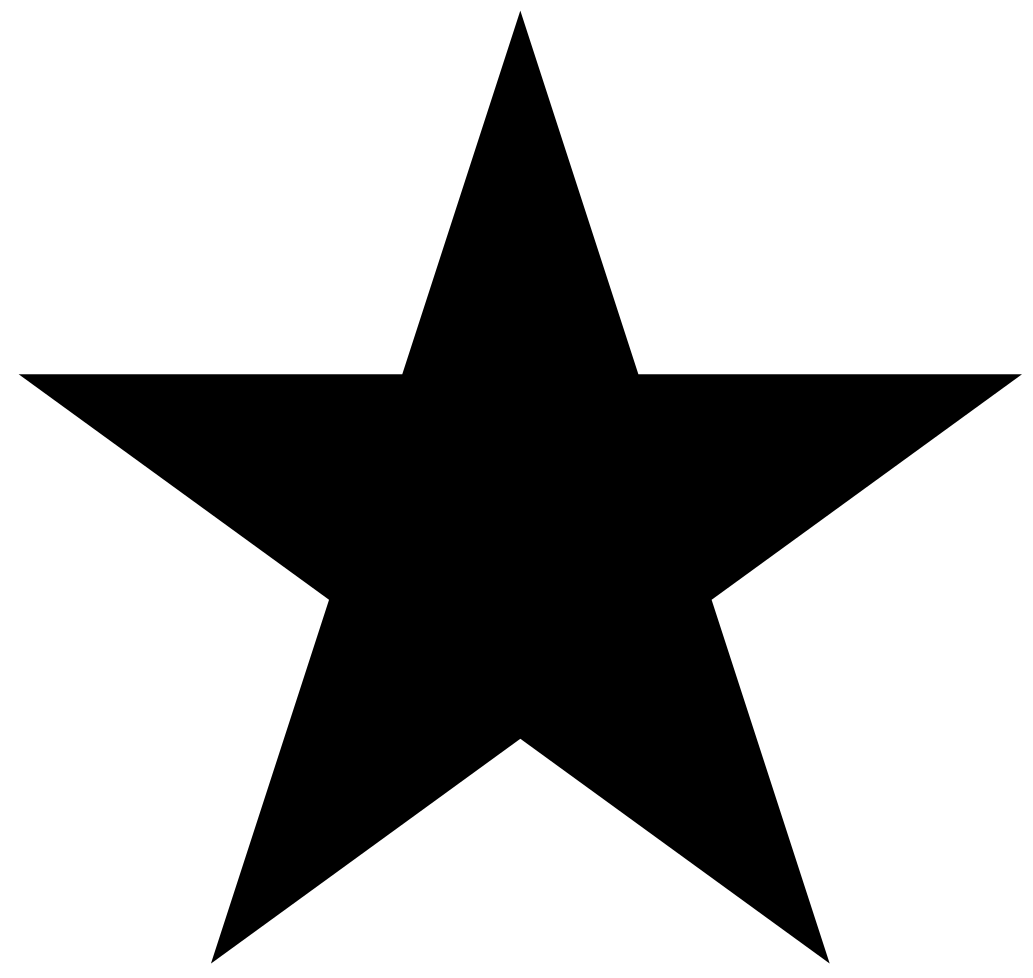 | 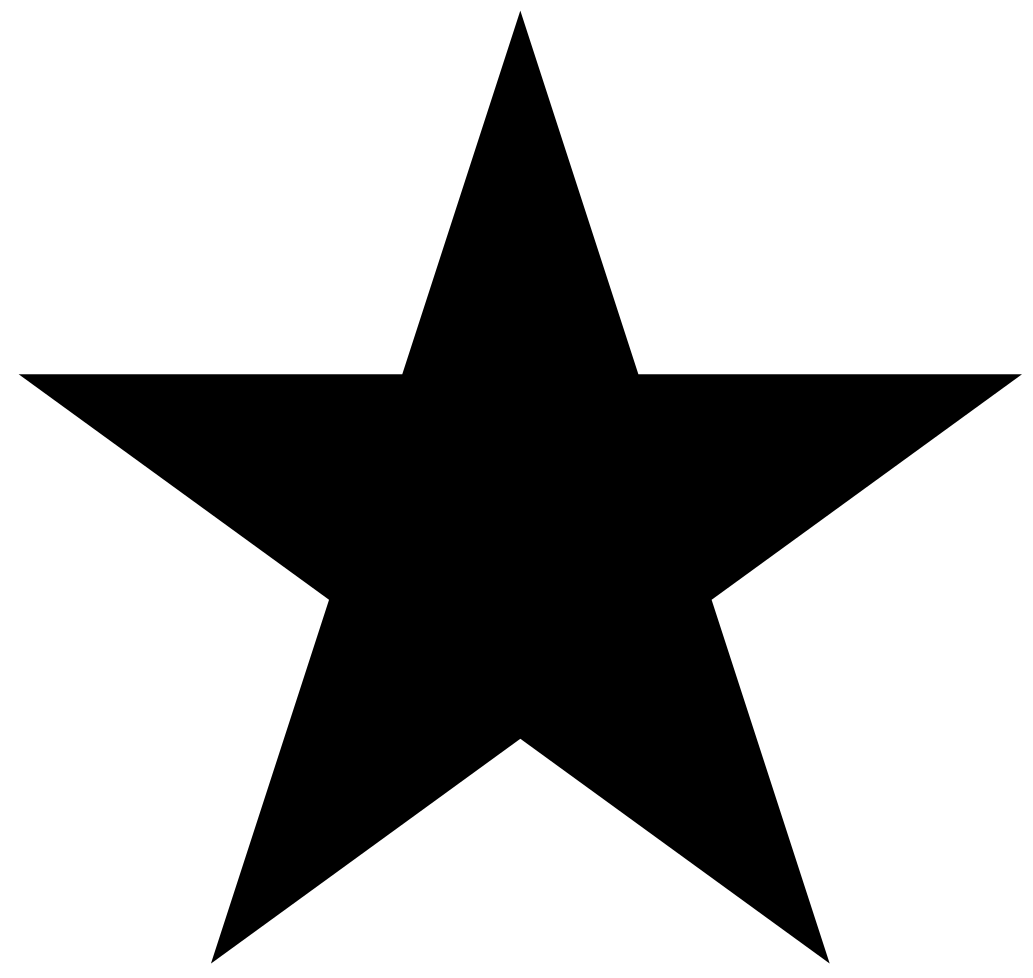 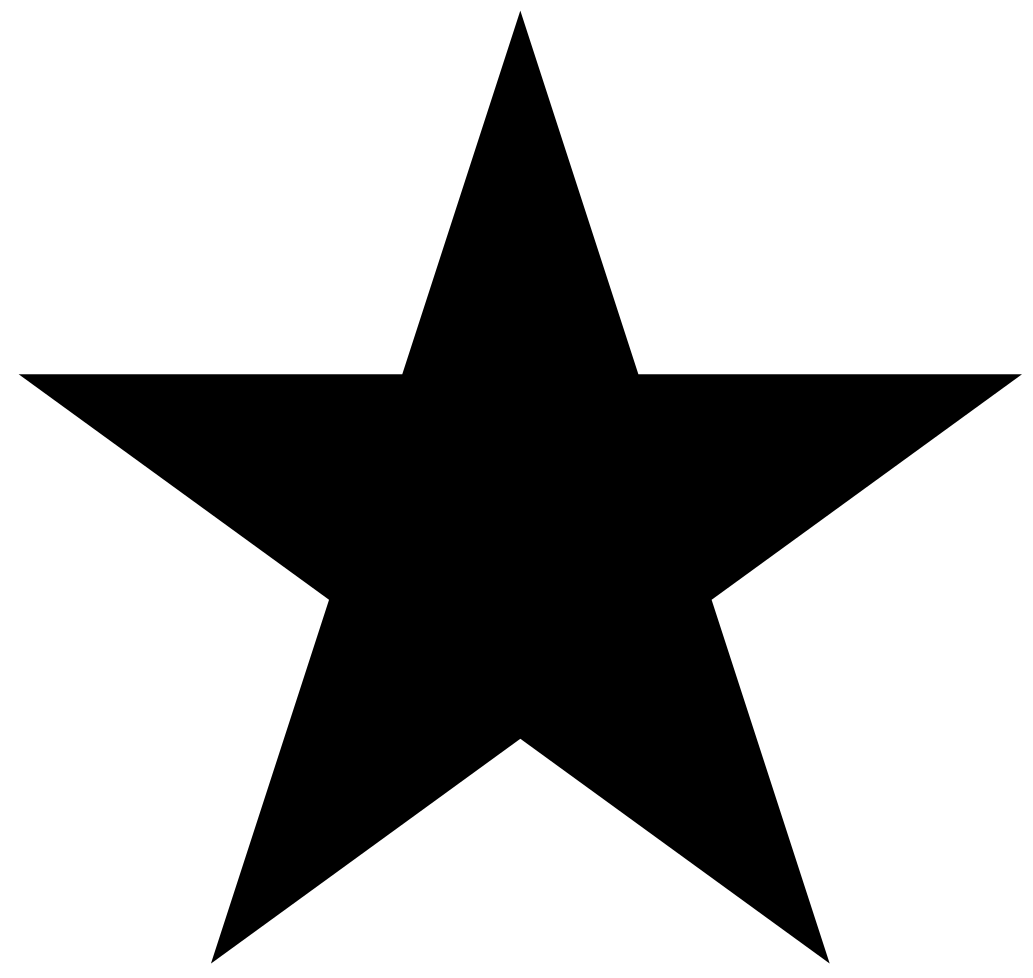 **6** |
| Cursino et al. (2018) 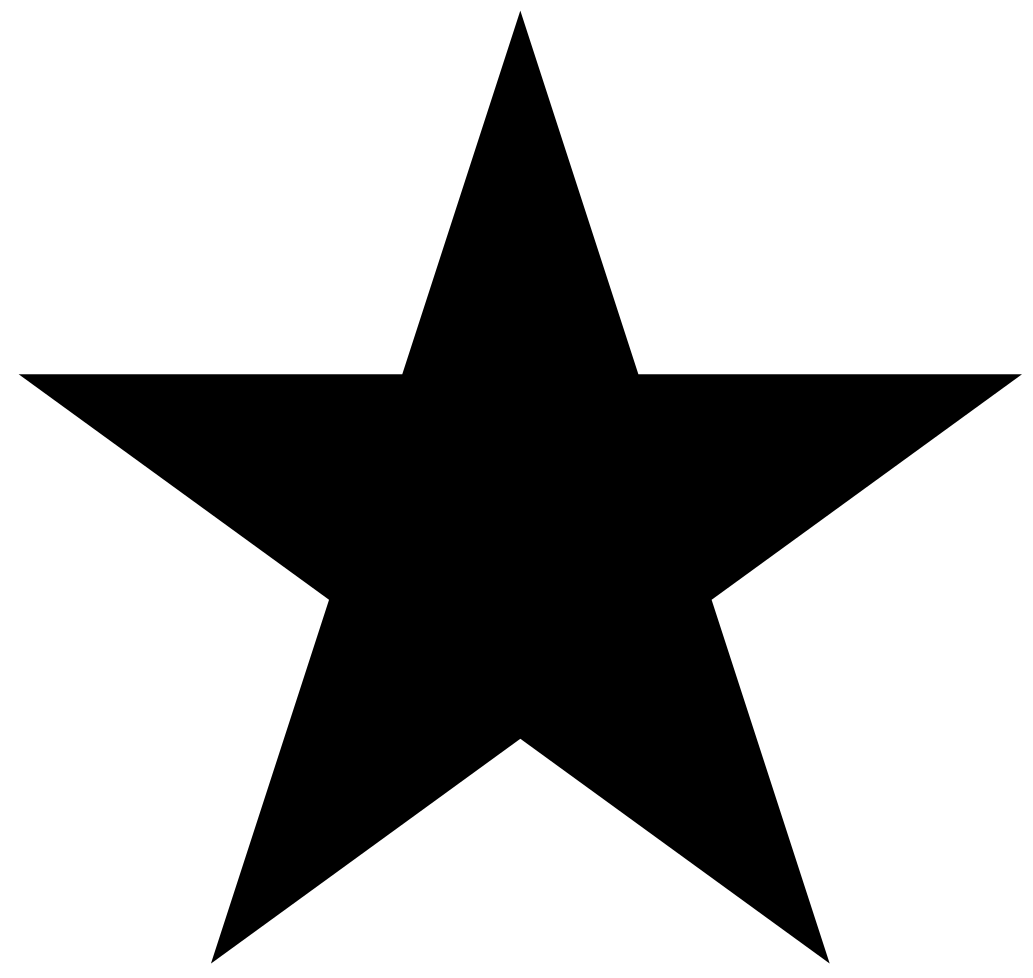 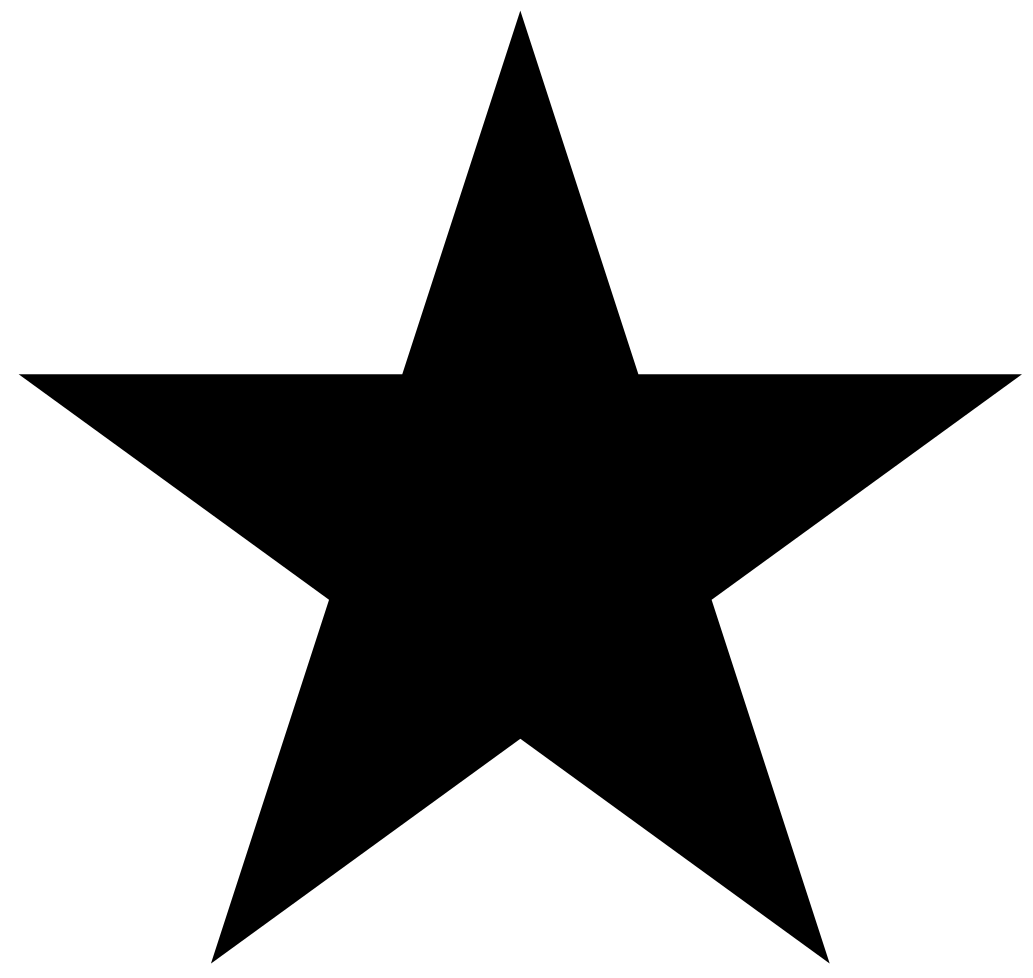 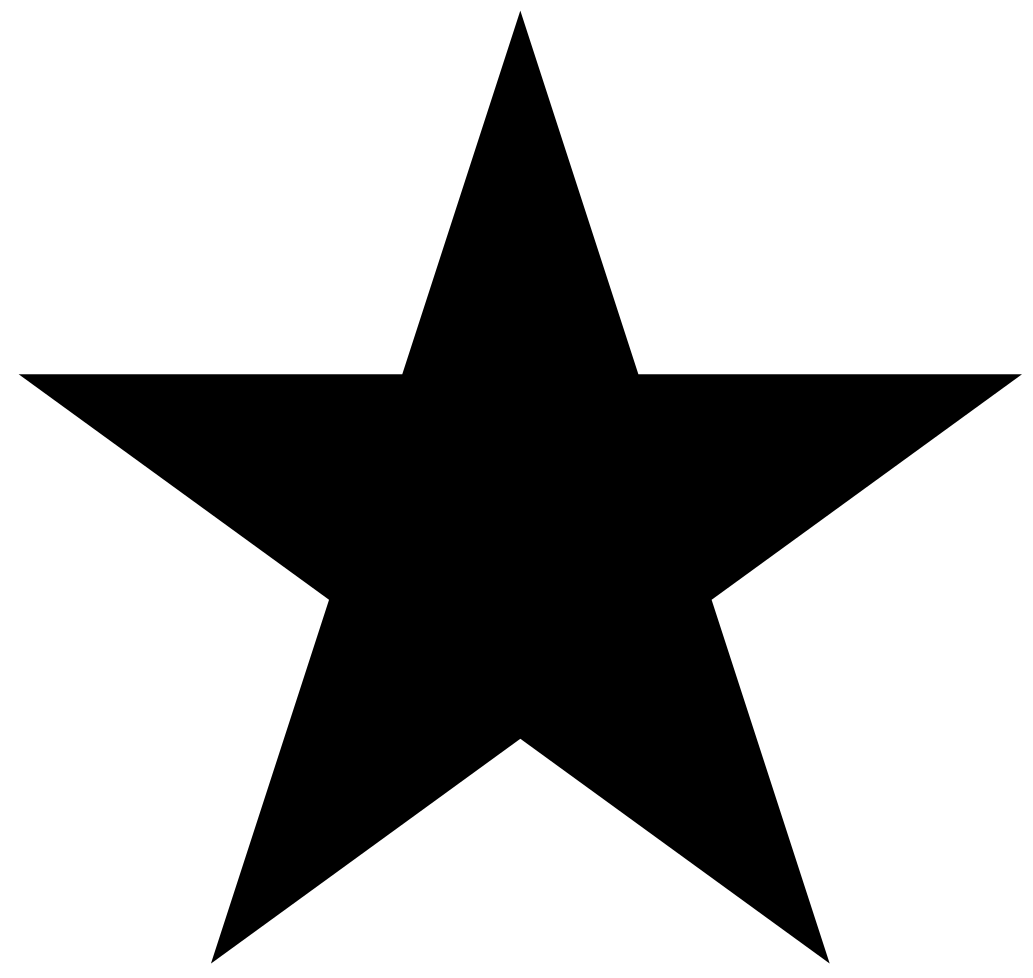 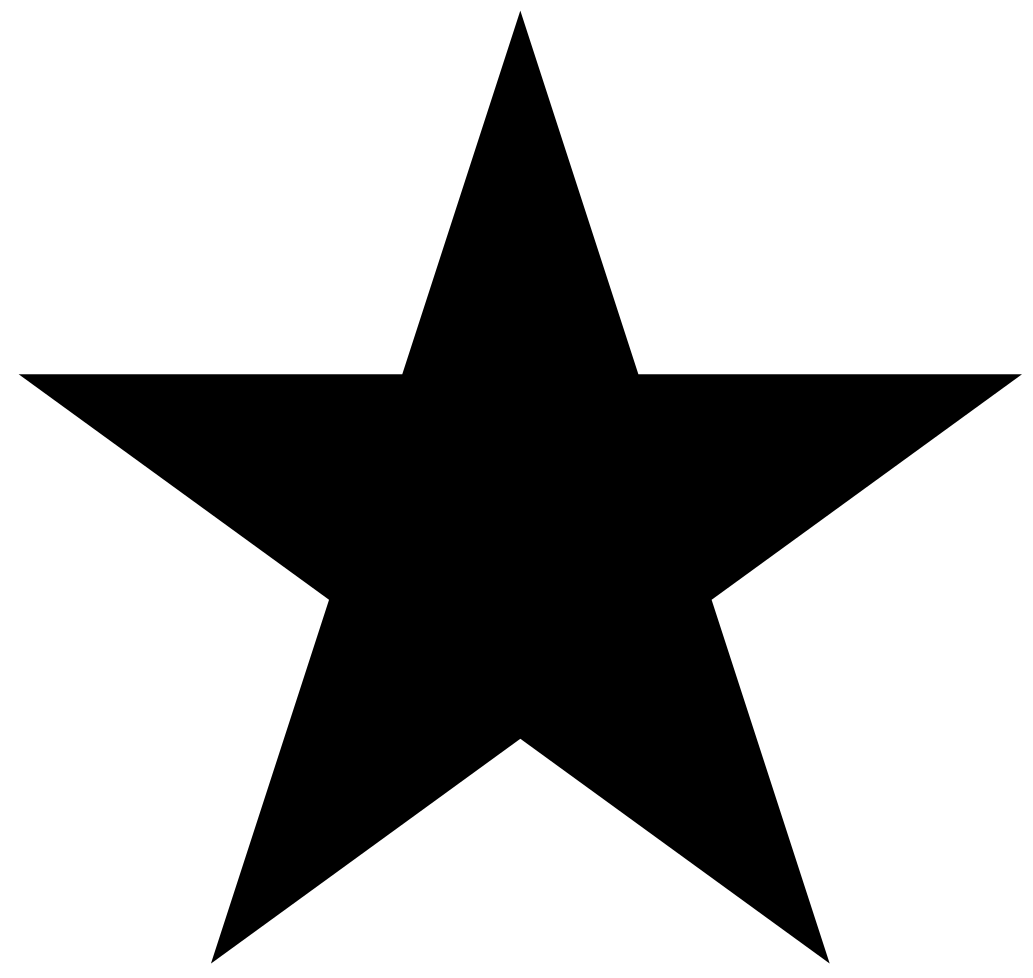 | 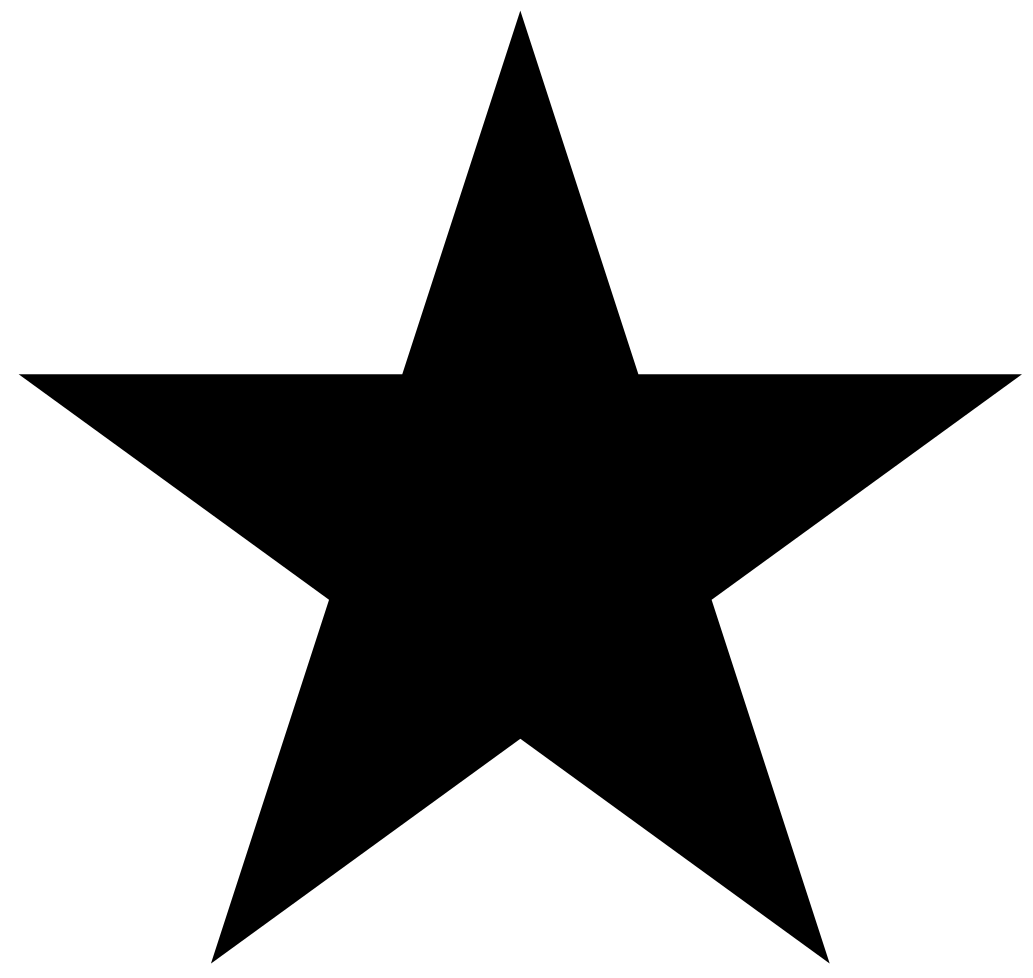 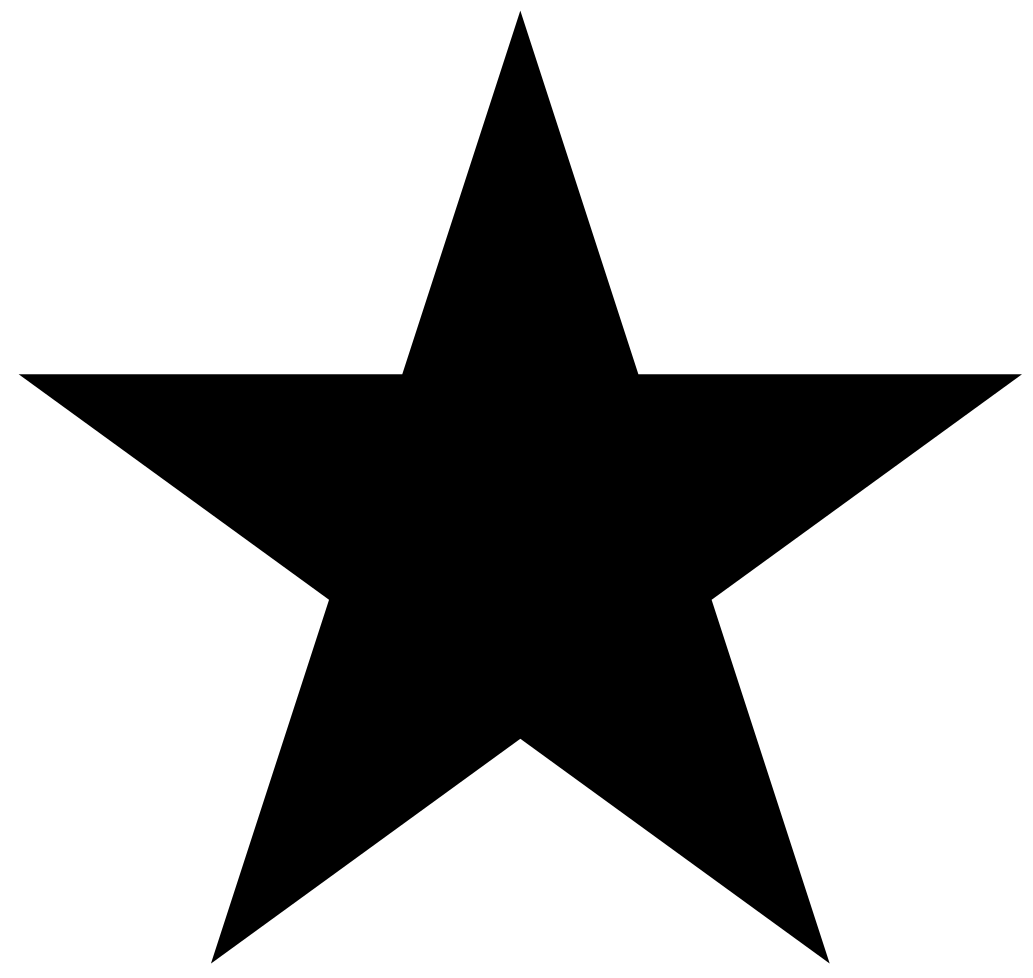 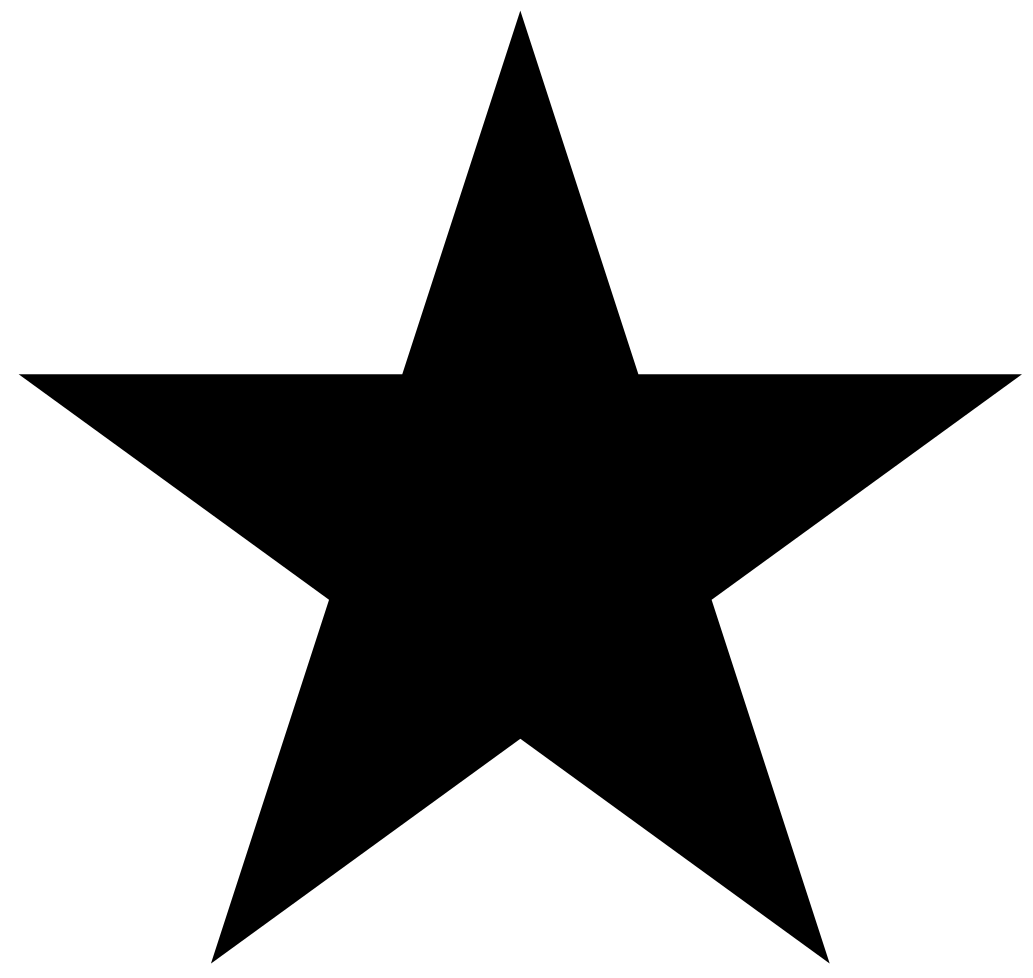 **7** |

Note: (1) Representativeness of the exposed cohort; (2) Selection of the non exposed cohort;

(3) ascertainment of exposure; (4) demonstration that outcome of interest was not present at the start of the study; (5) comparability of cohorts on the basis of the design or analysis; (6) assessment of outcome; (7) was follow-up long enough for outcome to occur?; (8) adequacy of follow up of cohorts.
