# Supplementary material for: The effect of non‐oral hormonal contraceptives on hypertension and blood pressure: A systematic review and meta‐analysis
Source: Physiol Rep. 2022 May 4;10(9):e15267. doi: 10.14814/phy2.15267 (PMC9069167; doi:10.14814/phy2.15267)
Supplement: Supplementary file 5 — Table S3 [file PHY2-10-e15267-s004.docx]

Table 3: Quality assessment of the cross sectional studies using New Castle Ottawa Scale

**Selection**

**Comparability**

**Outcome**

**First Author (Year)**

**1**

**2 3 4**

**5**

**6**

**7**

**8**

**Total**

|  |  |
| --- | --- |
| Oyelola et al. (1993) 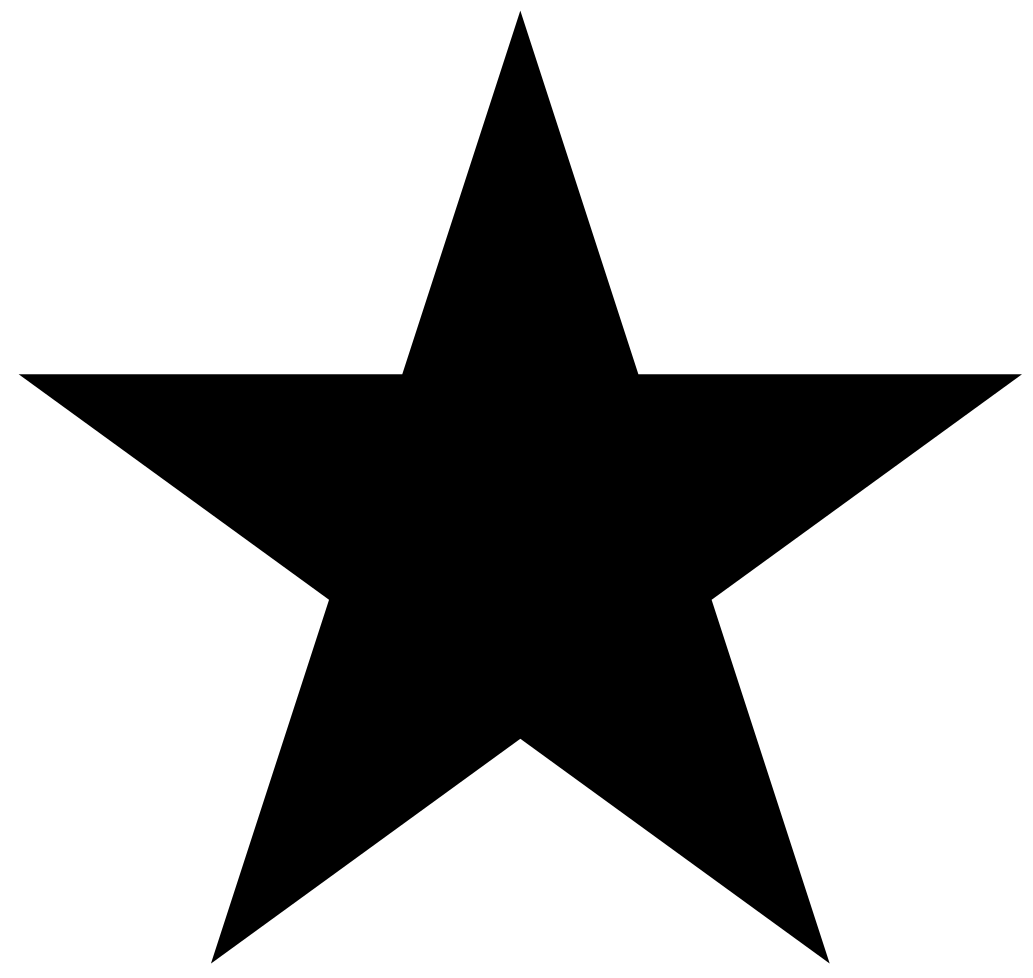 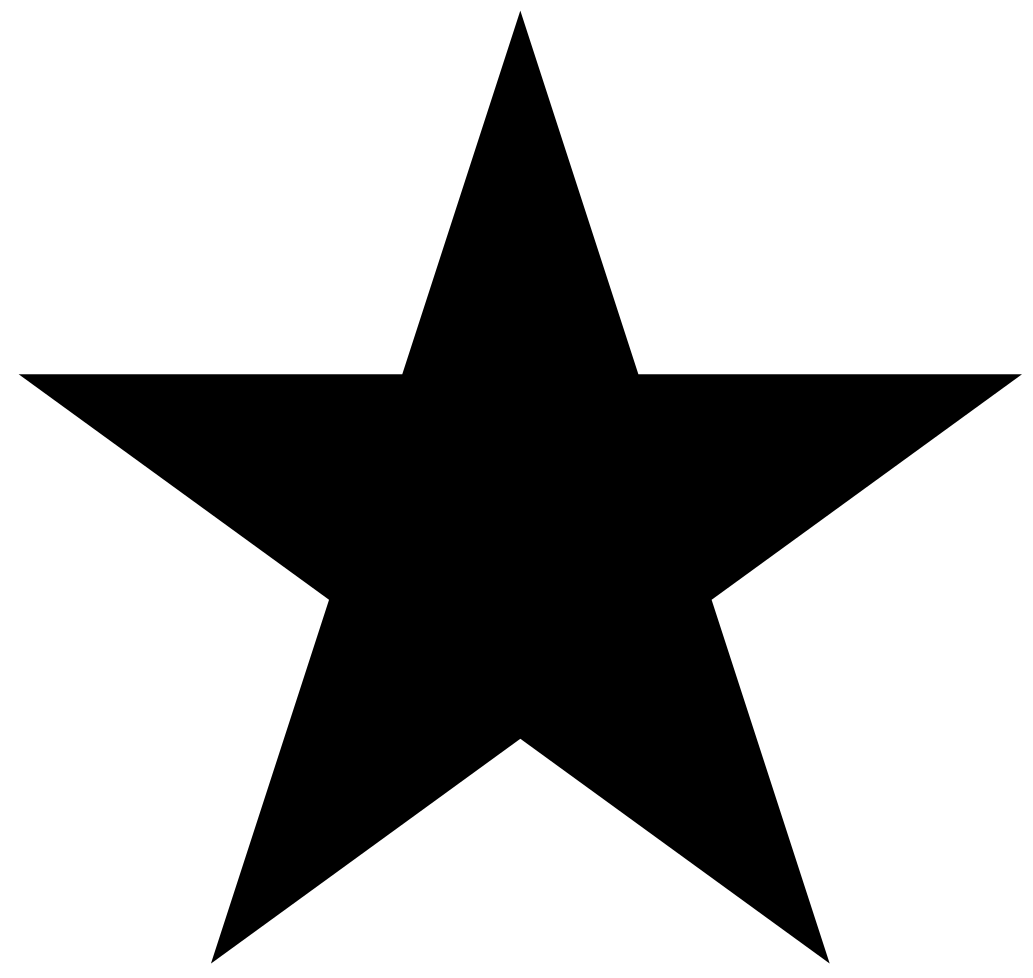 | 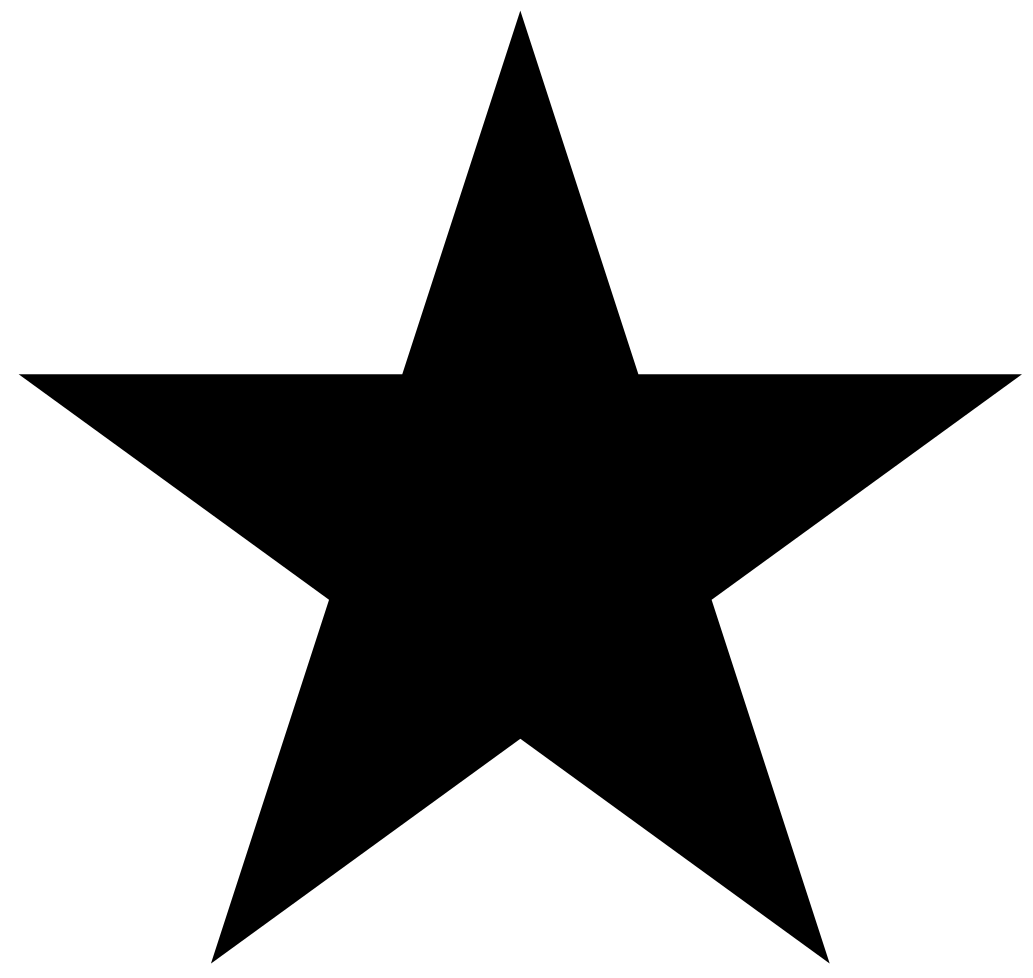 **4** |
| Yasmin et al. (1993) 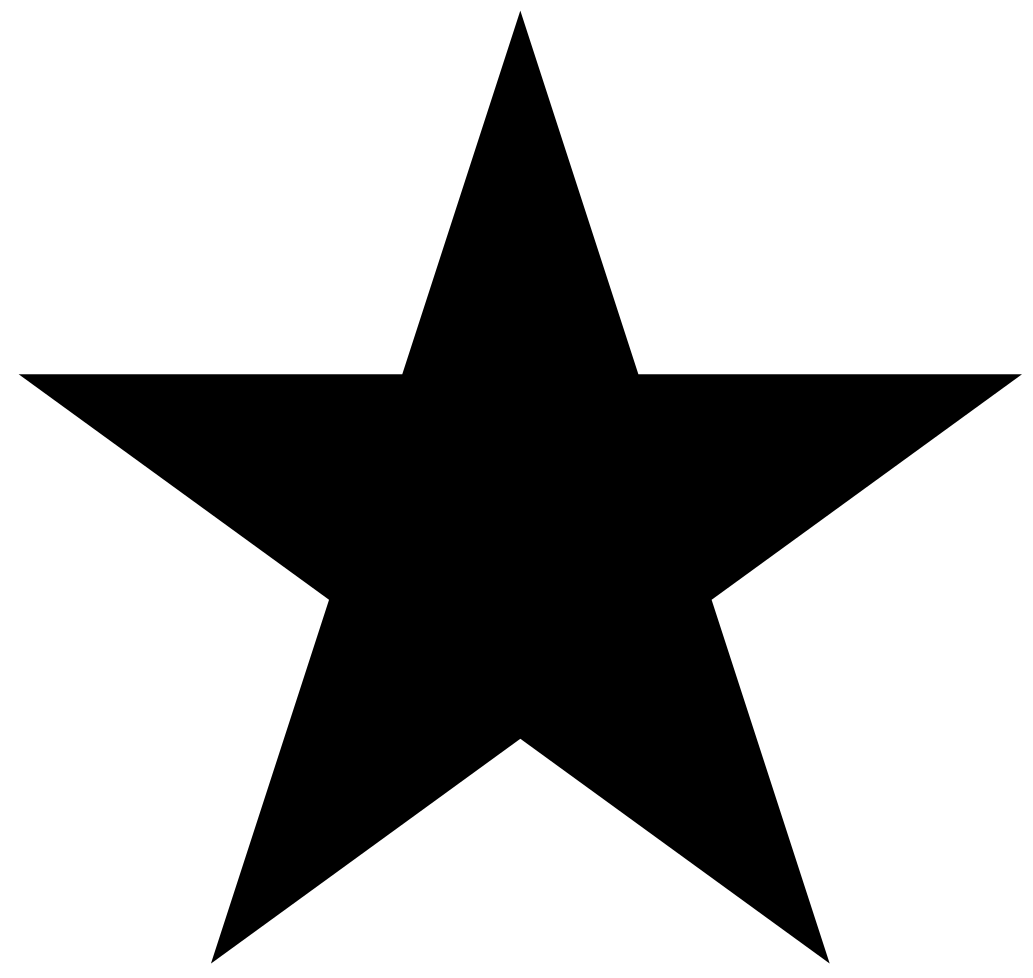 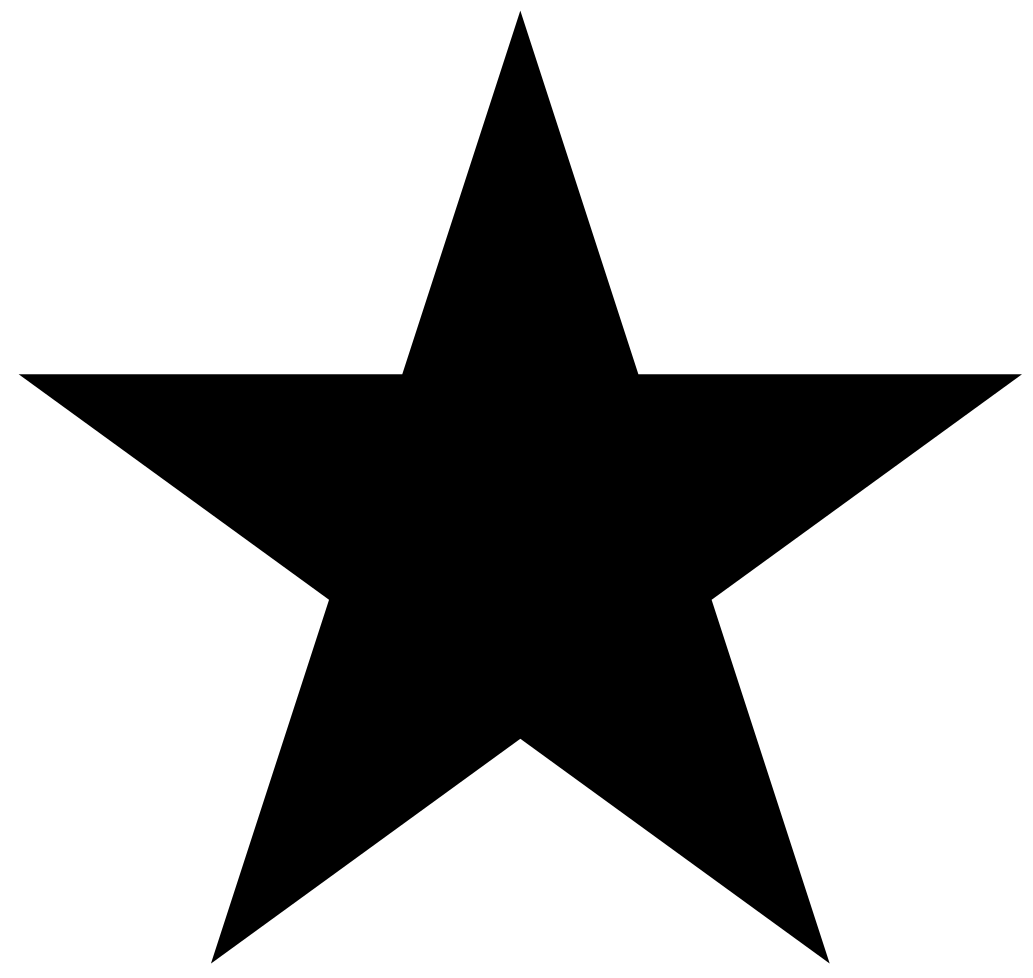 | 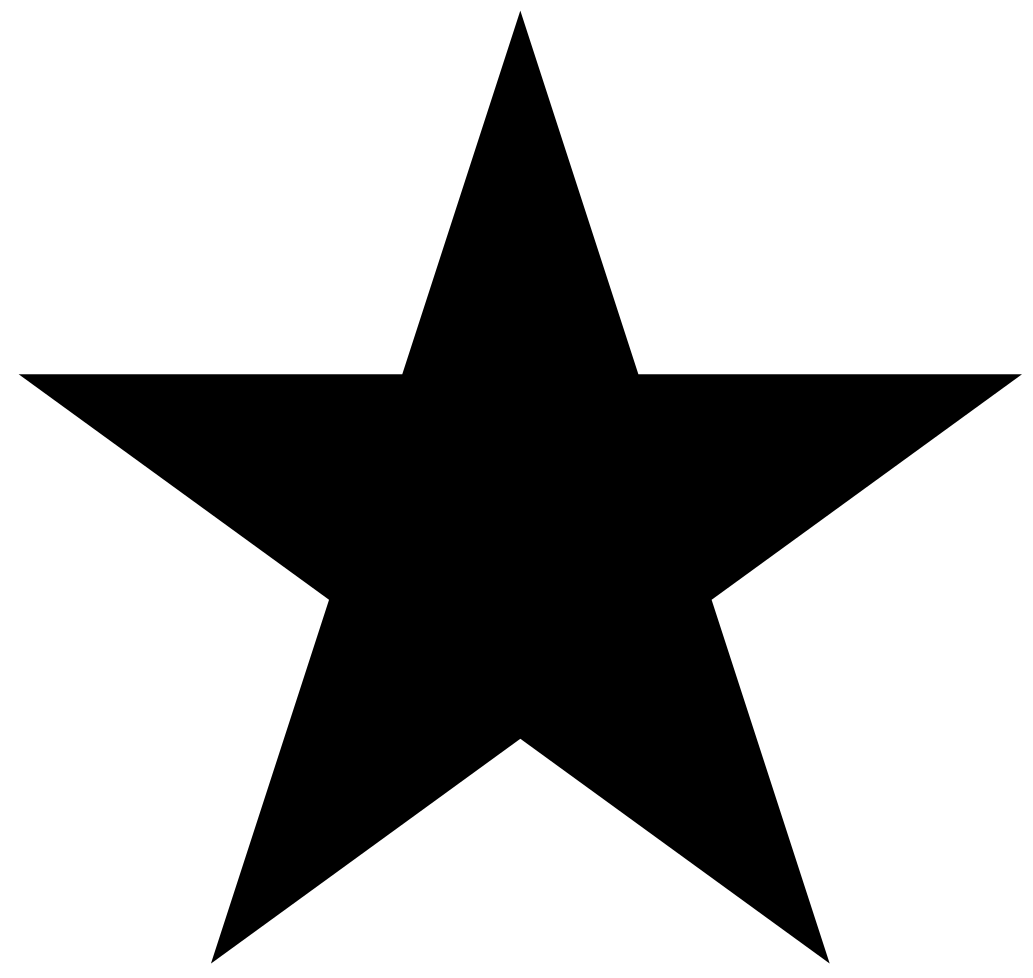 **3** |
| Taneepanichuskul et al. 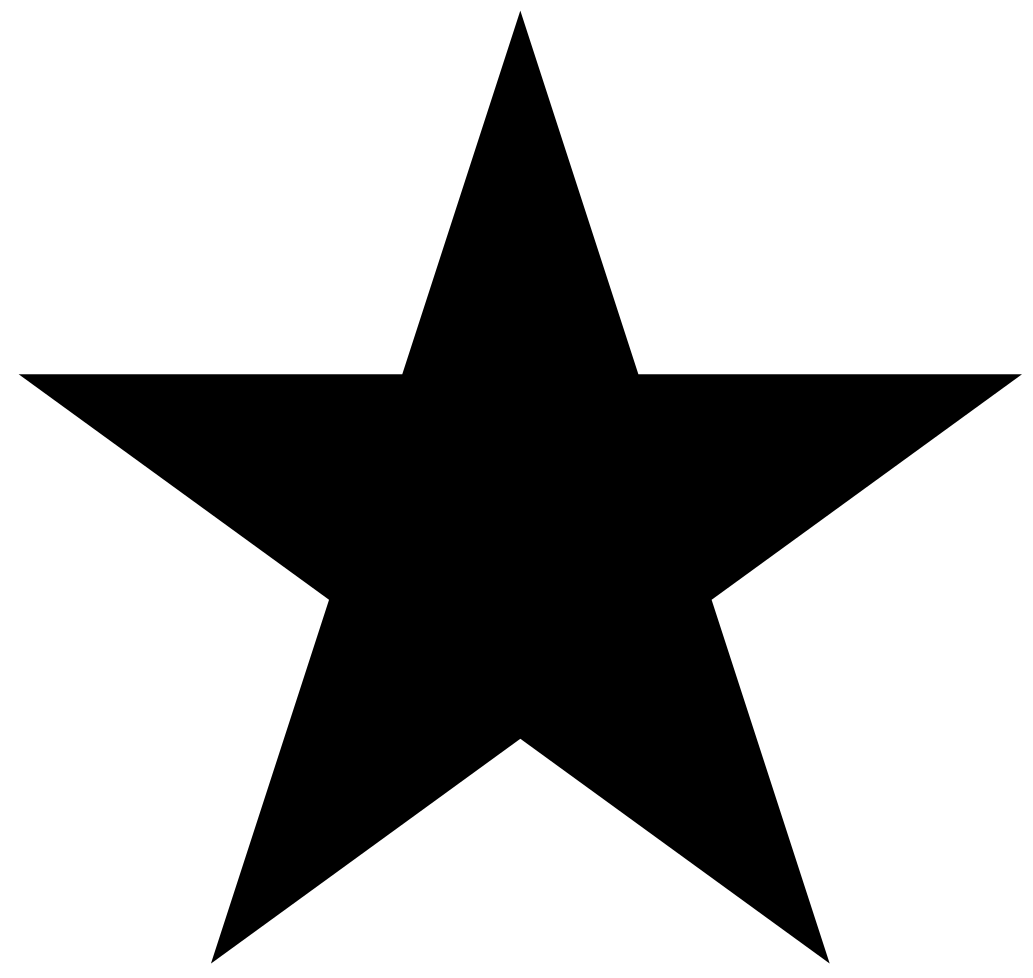 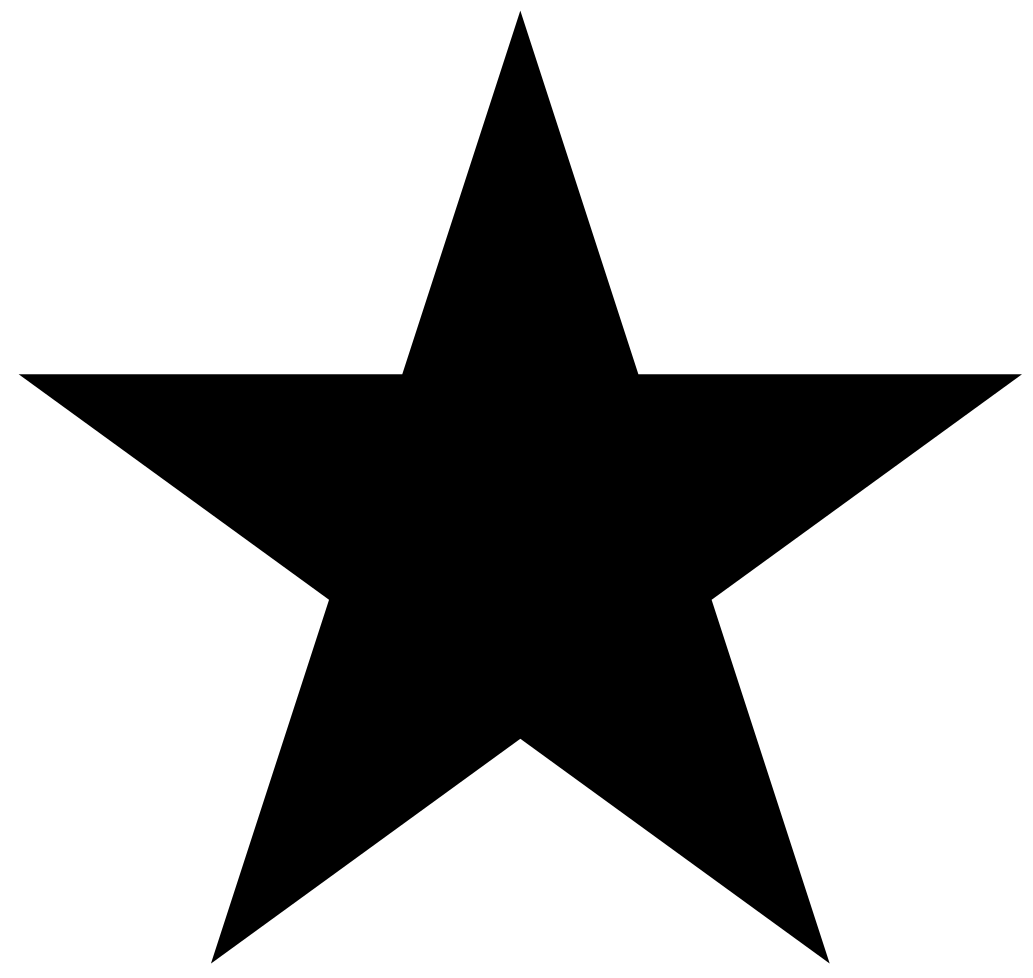 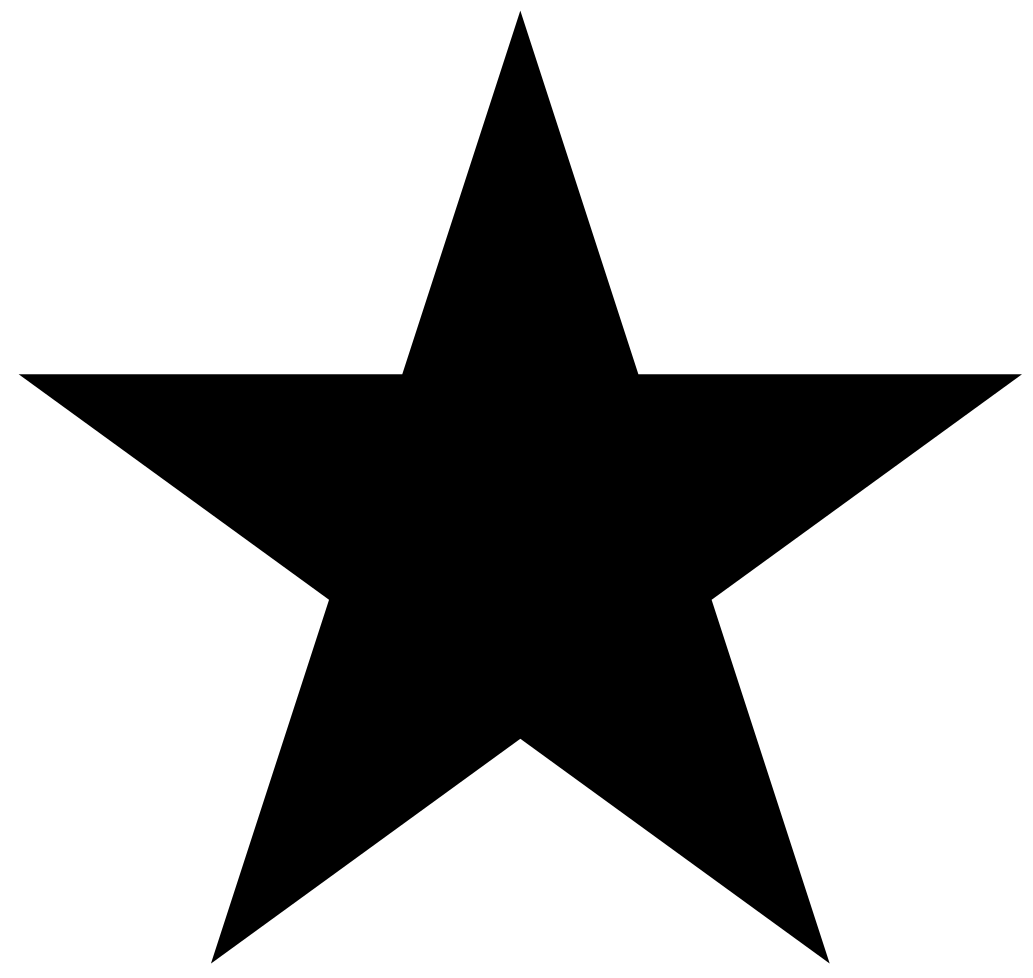 (1999) | 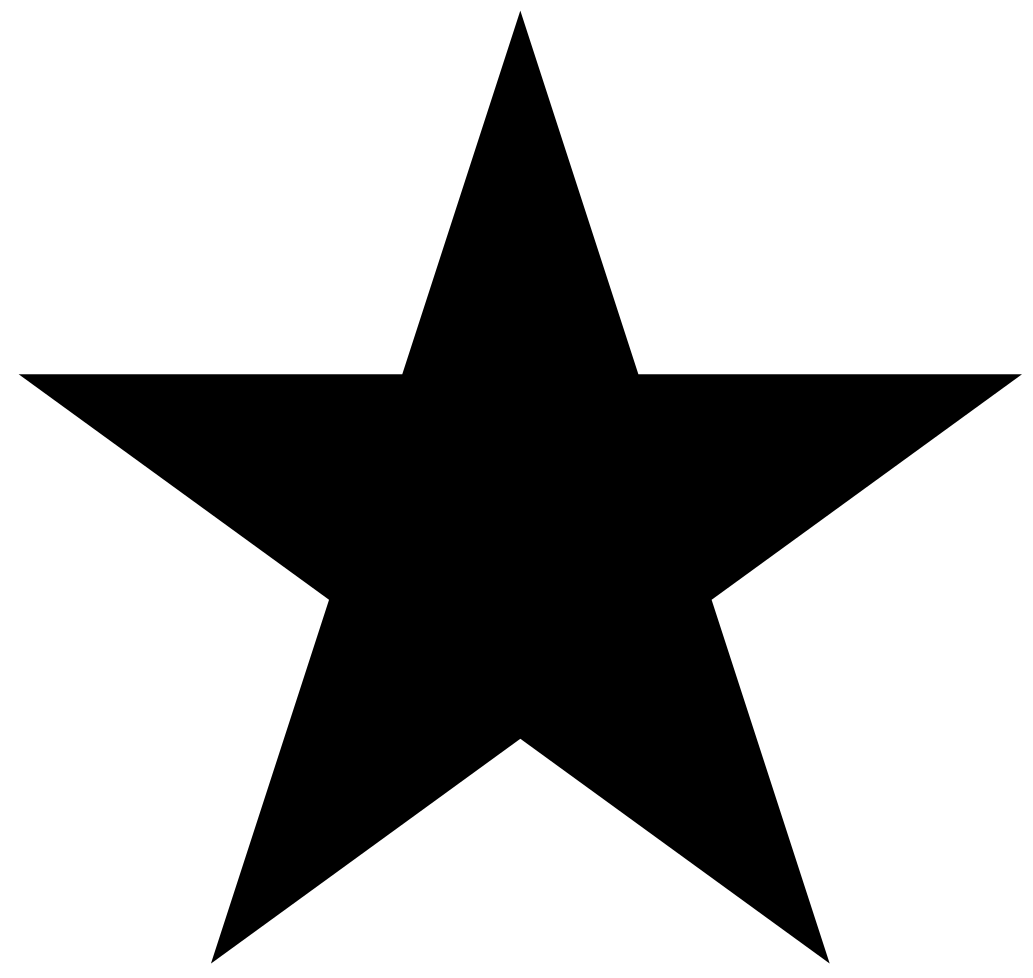 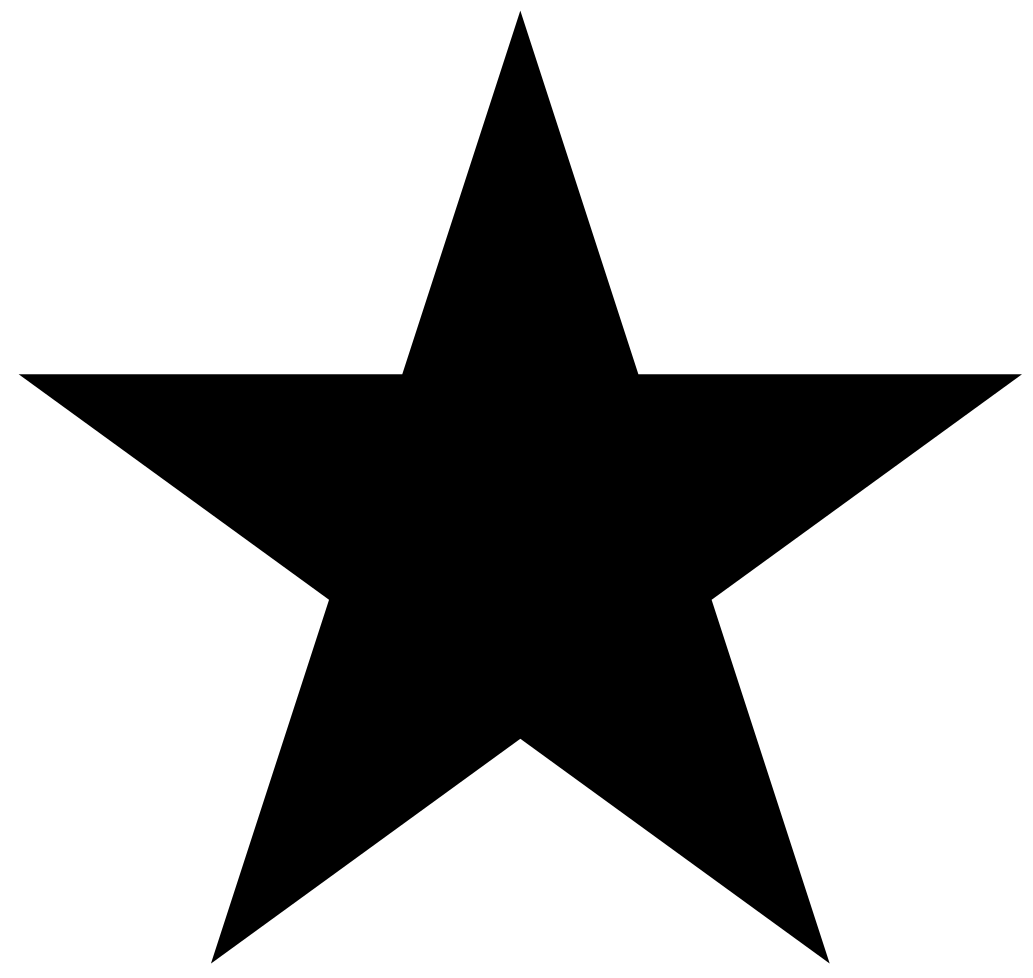 **5** |
| Mia et al. (2004) 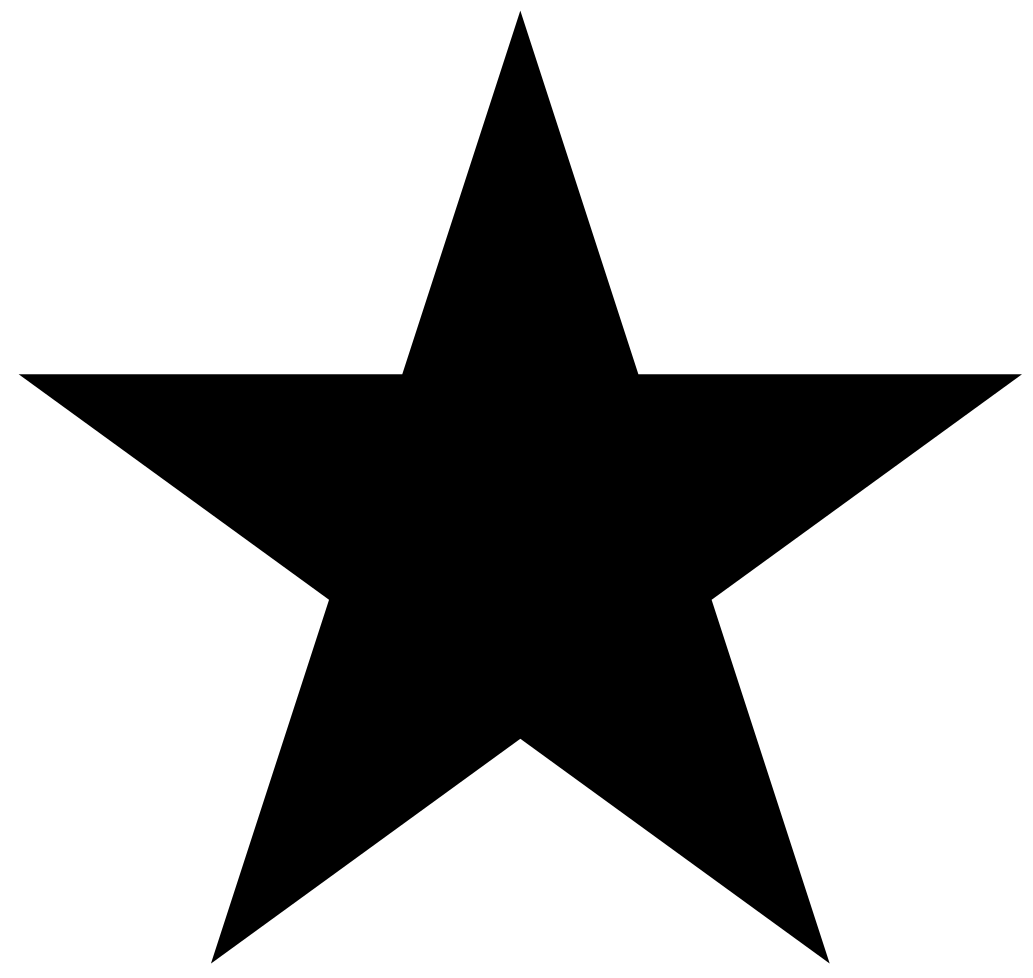 | 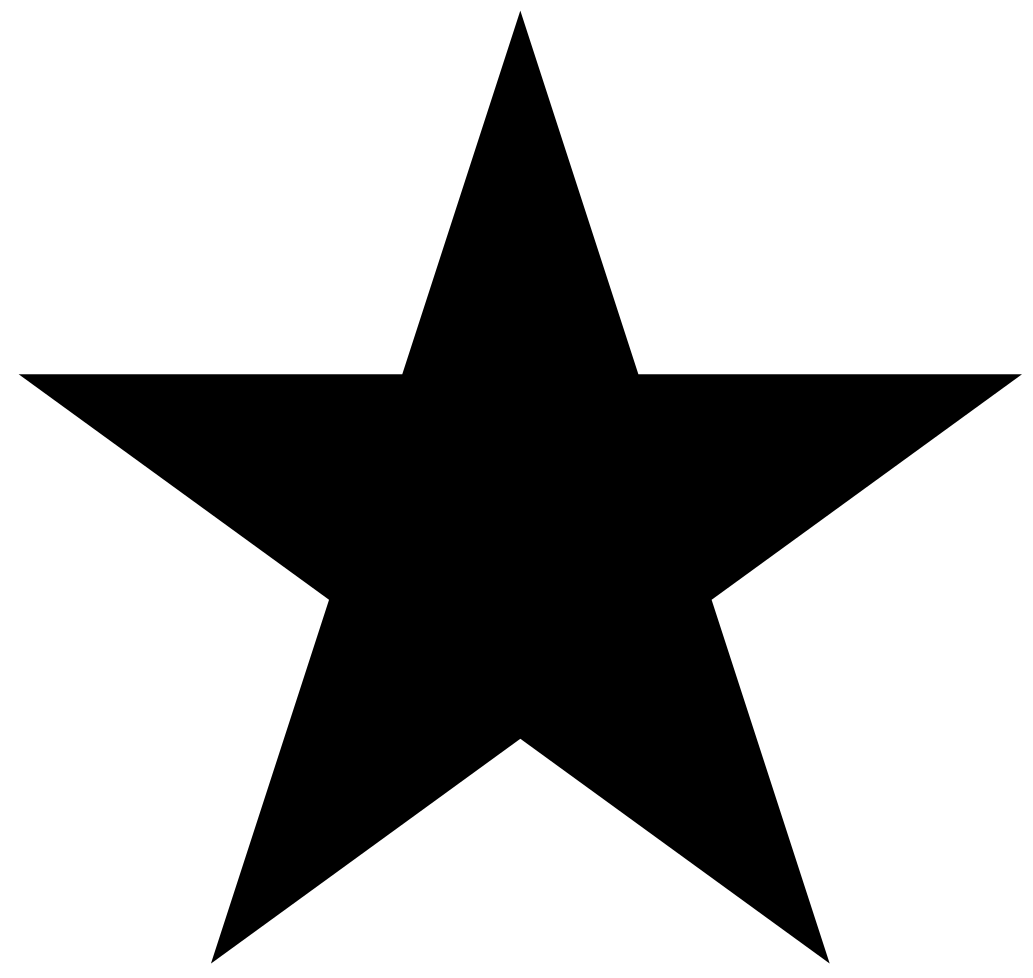 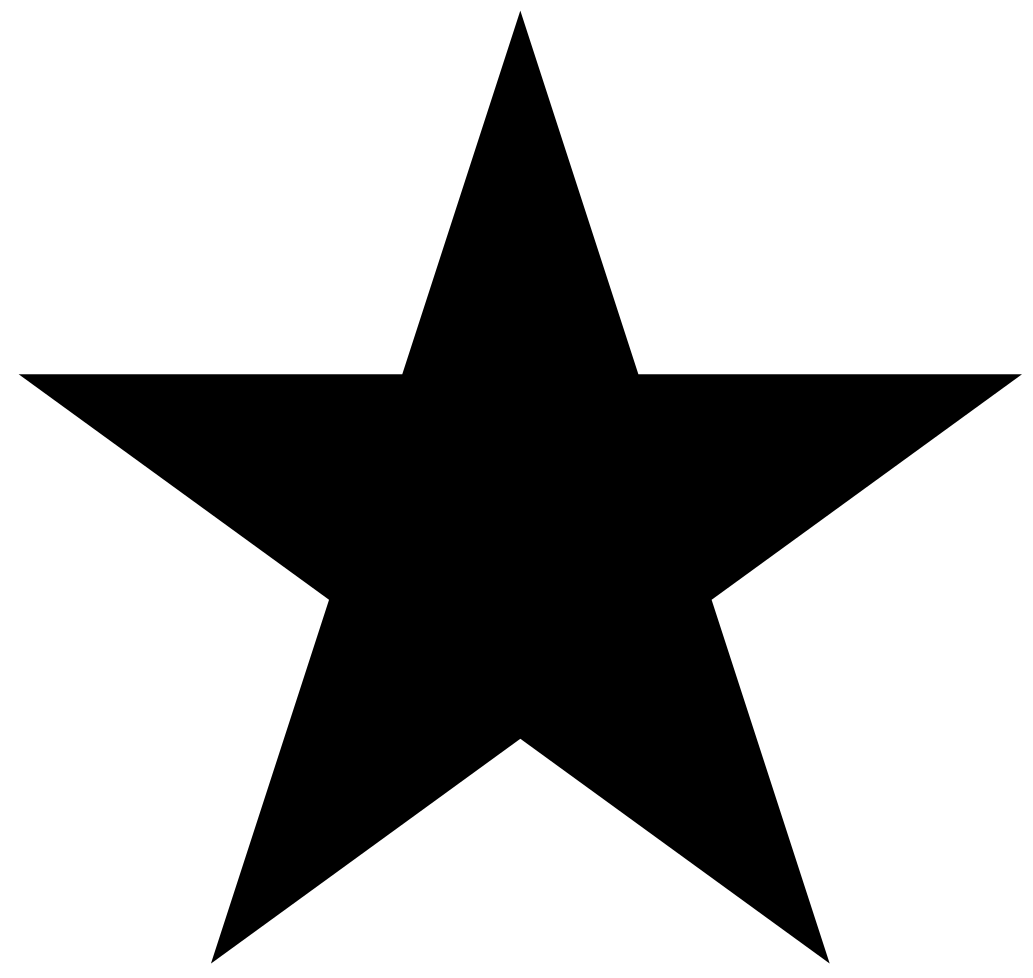 **3** |
| Morin-Papunen et al. 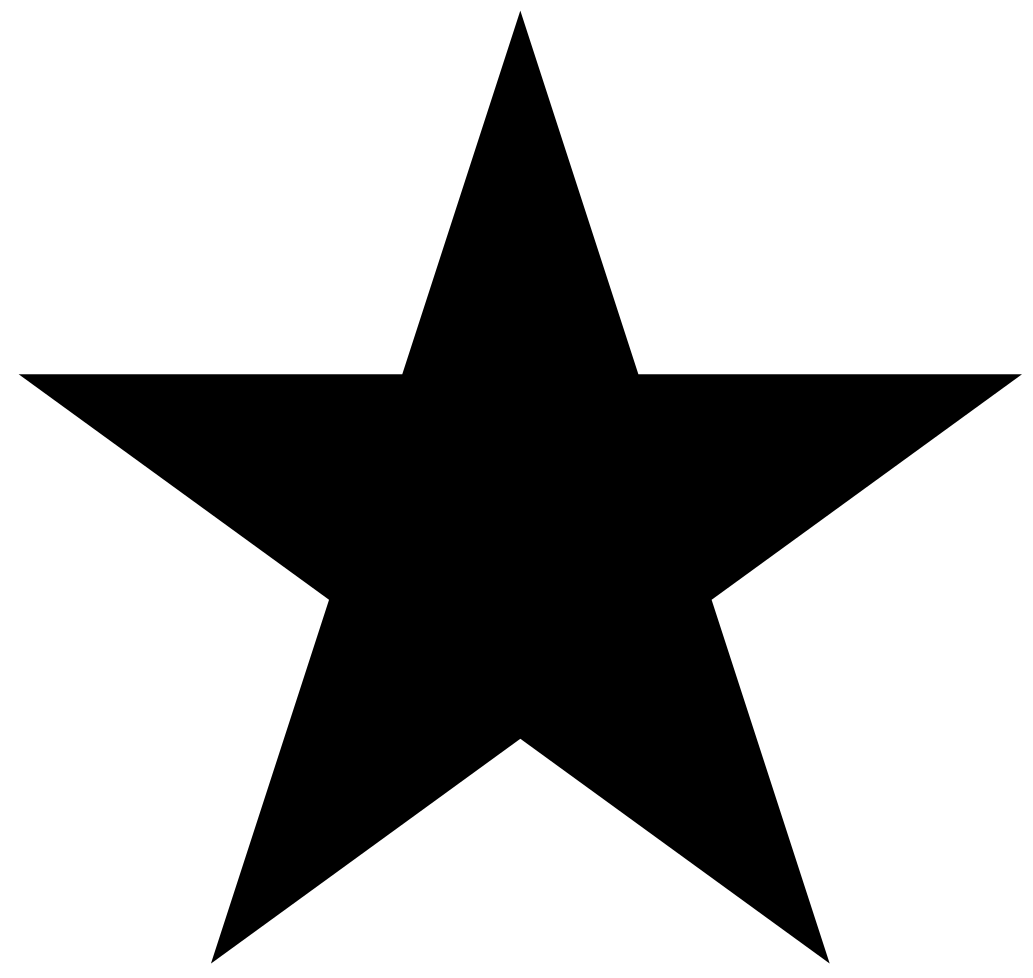 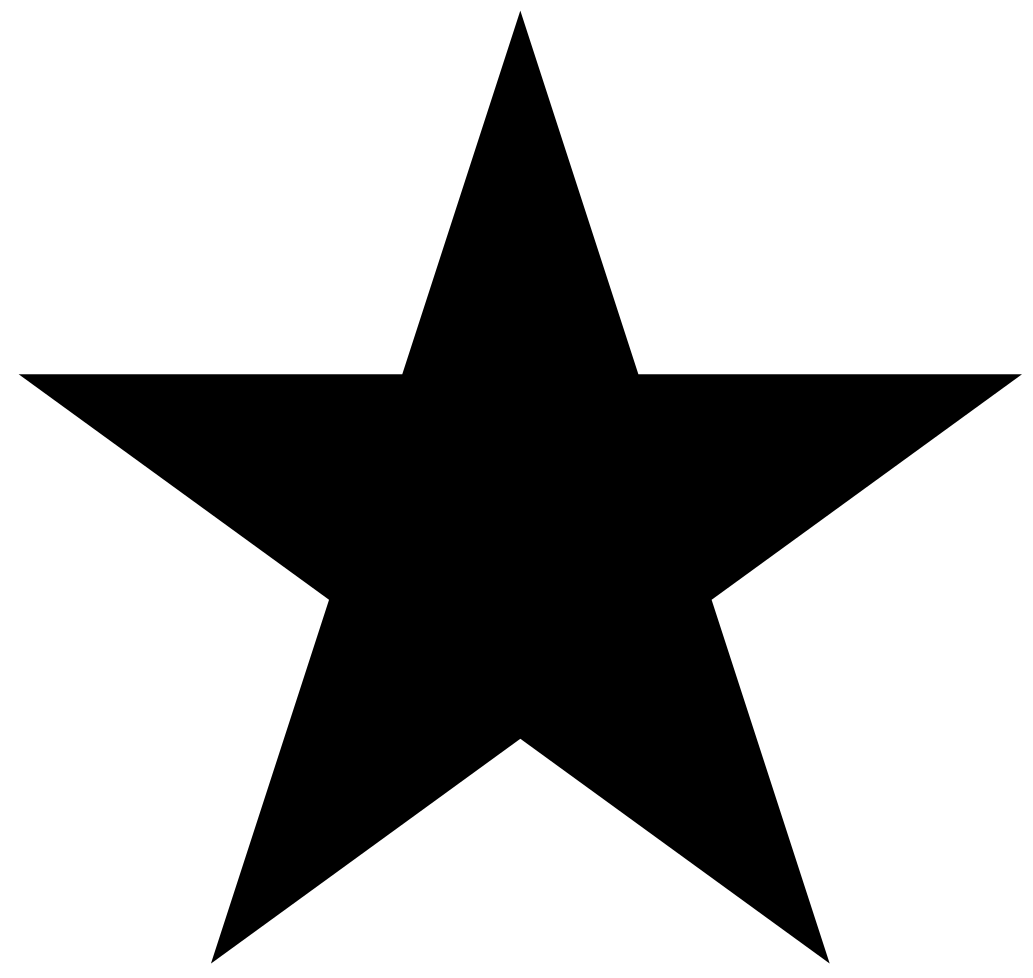 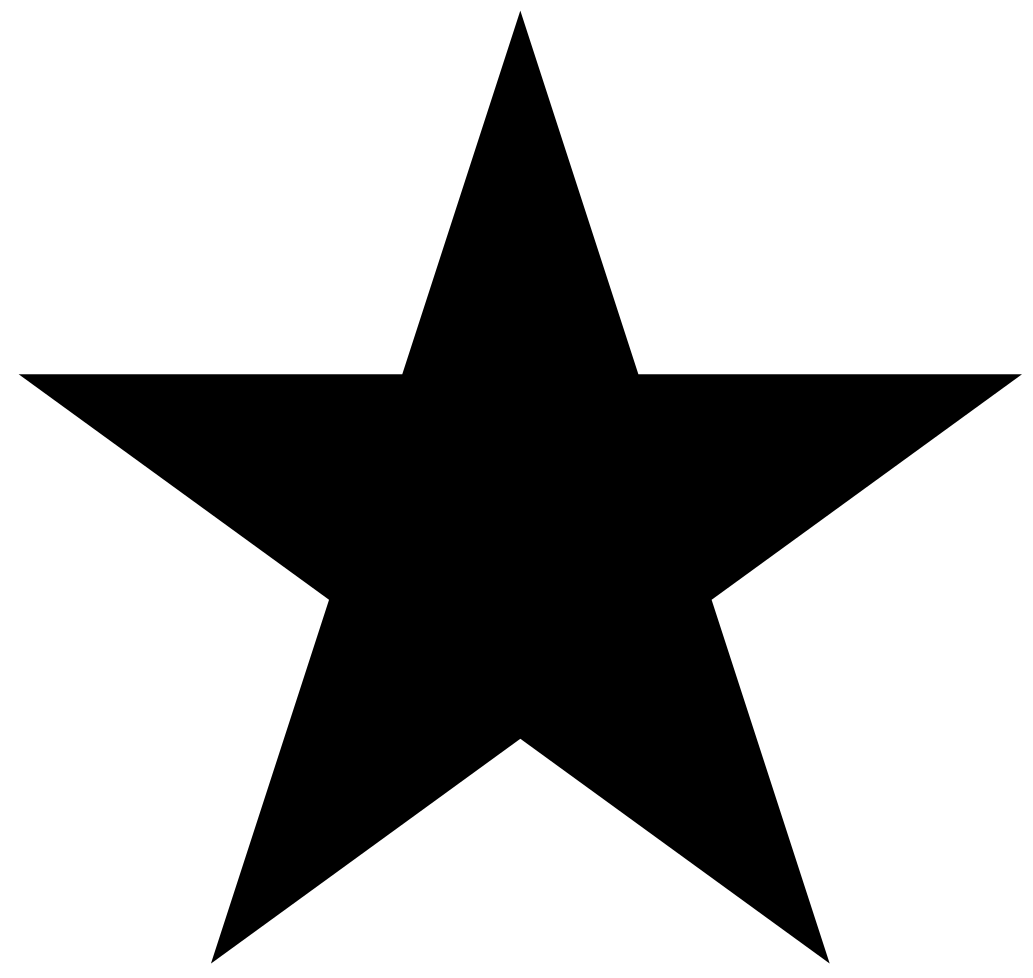 (2008) | 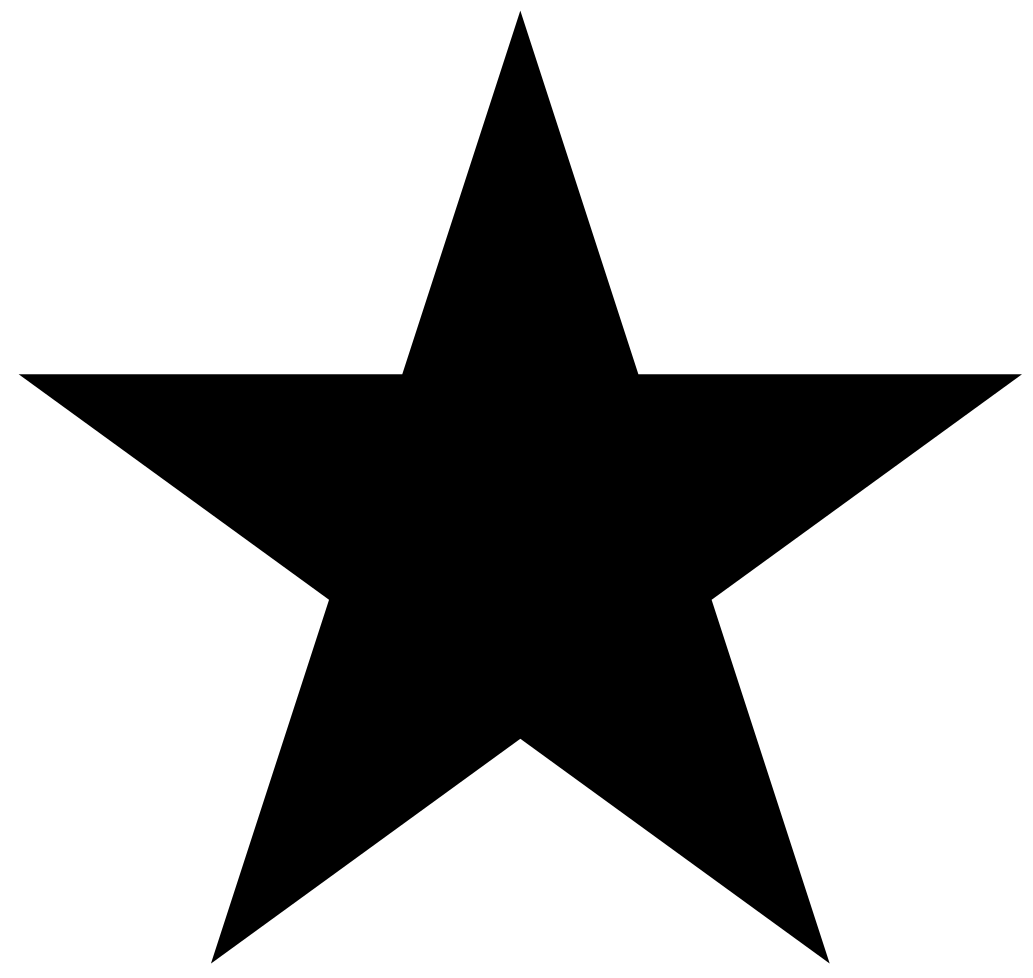 **4** |
| Lizarelli et al. (2009) 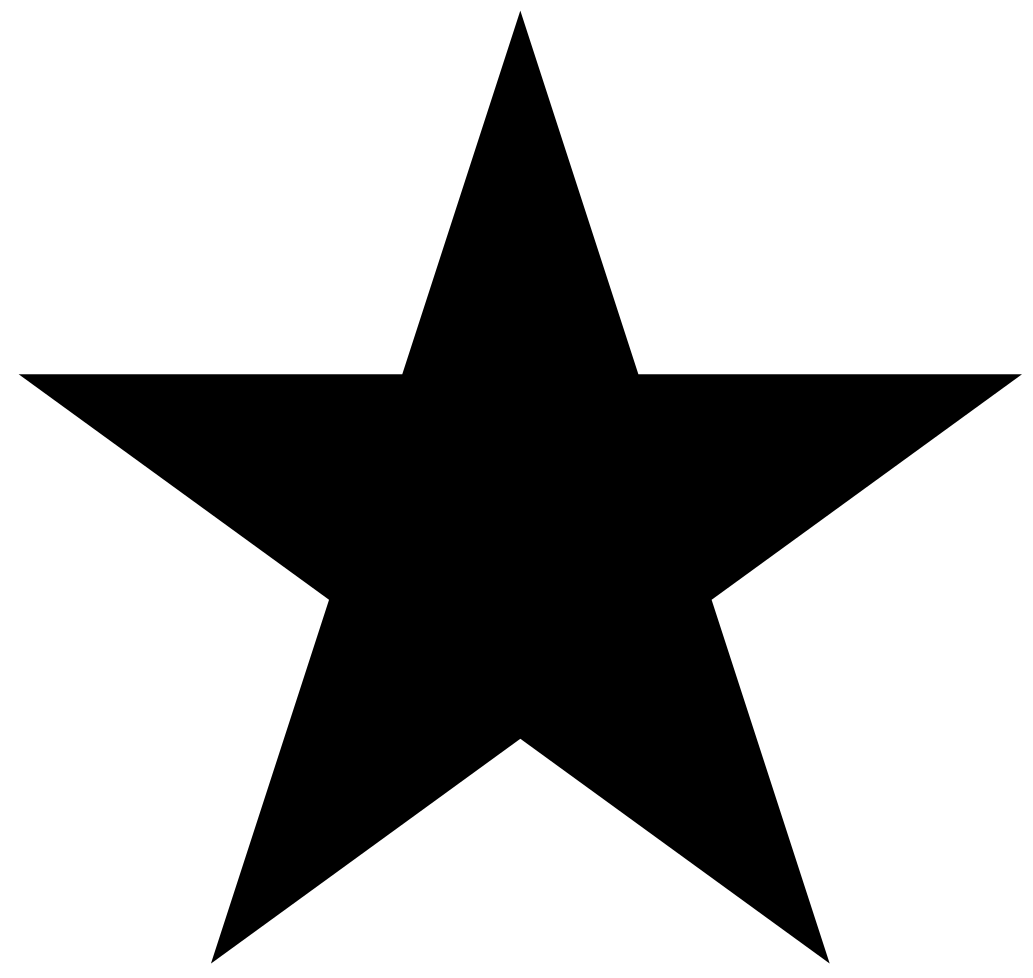 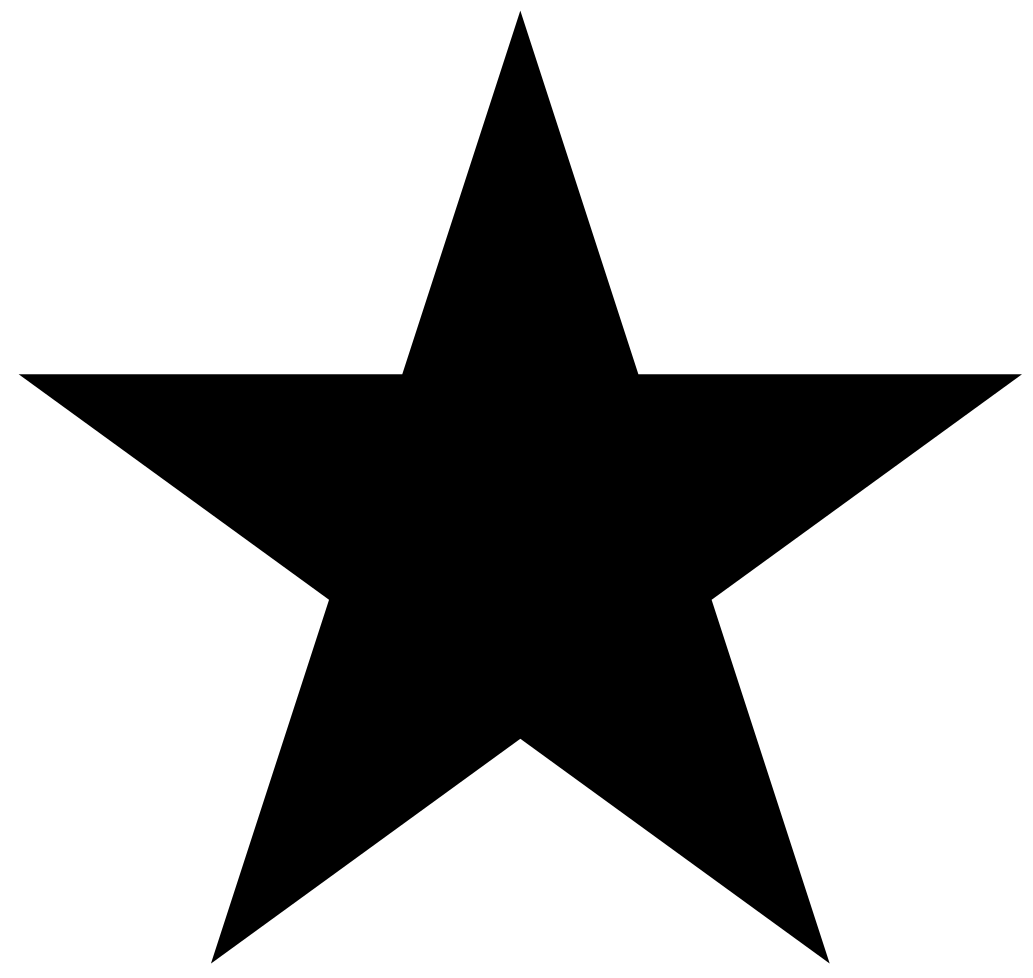 | 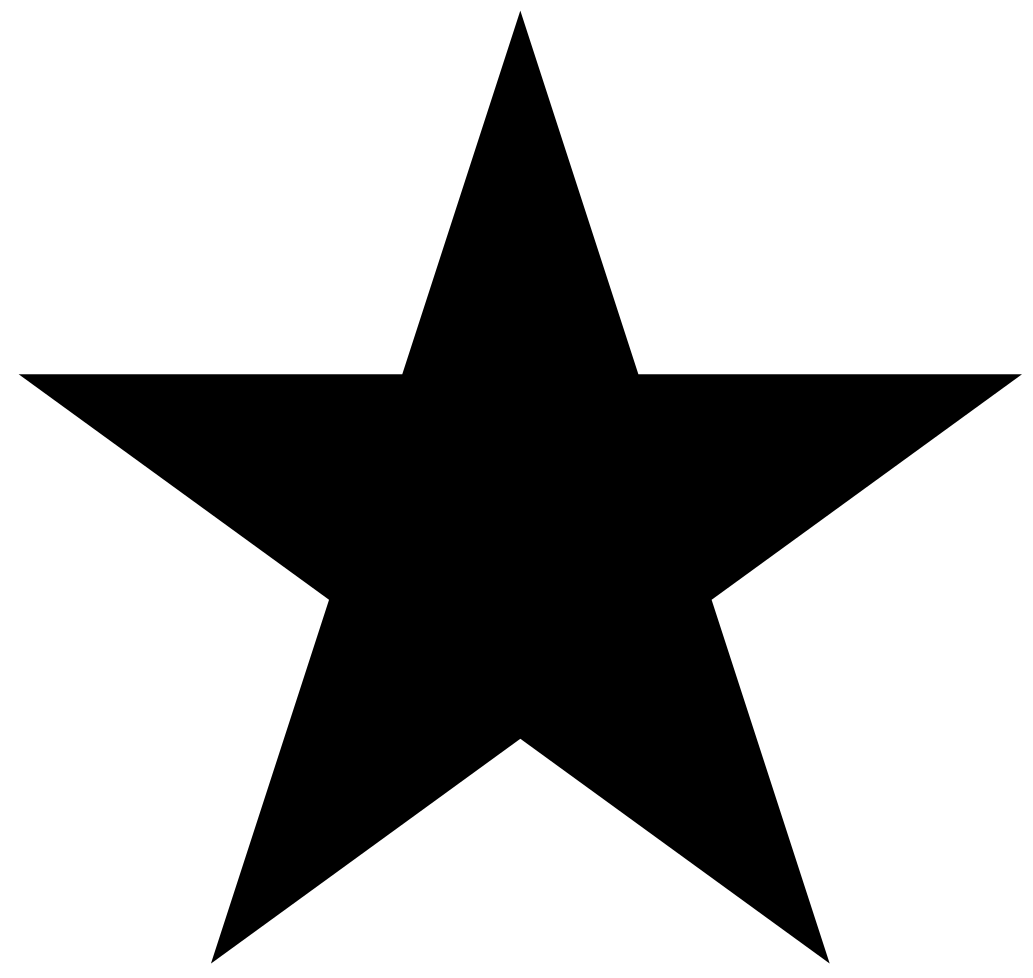 **3** |
| Al-Obaidy et al. (2014) 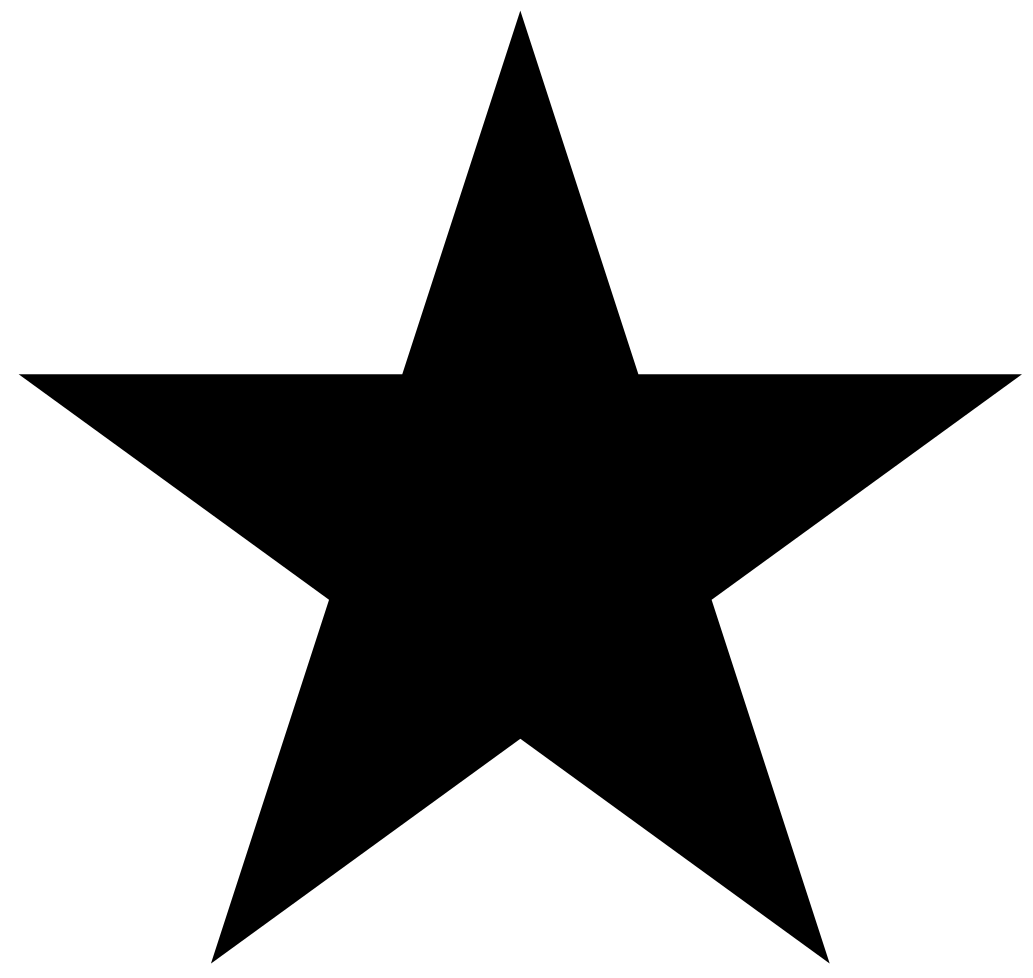 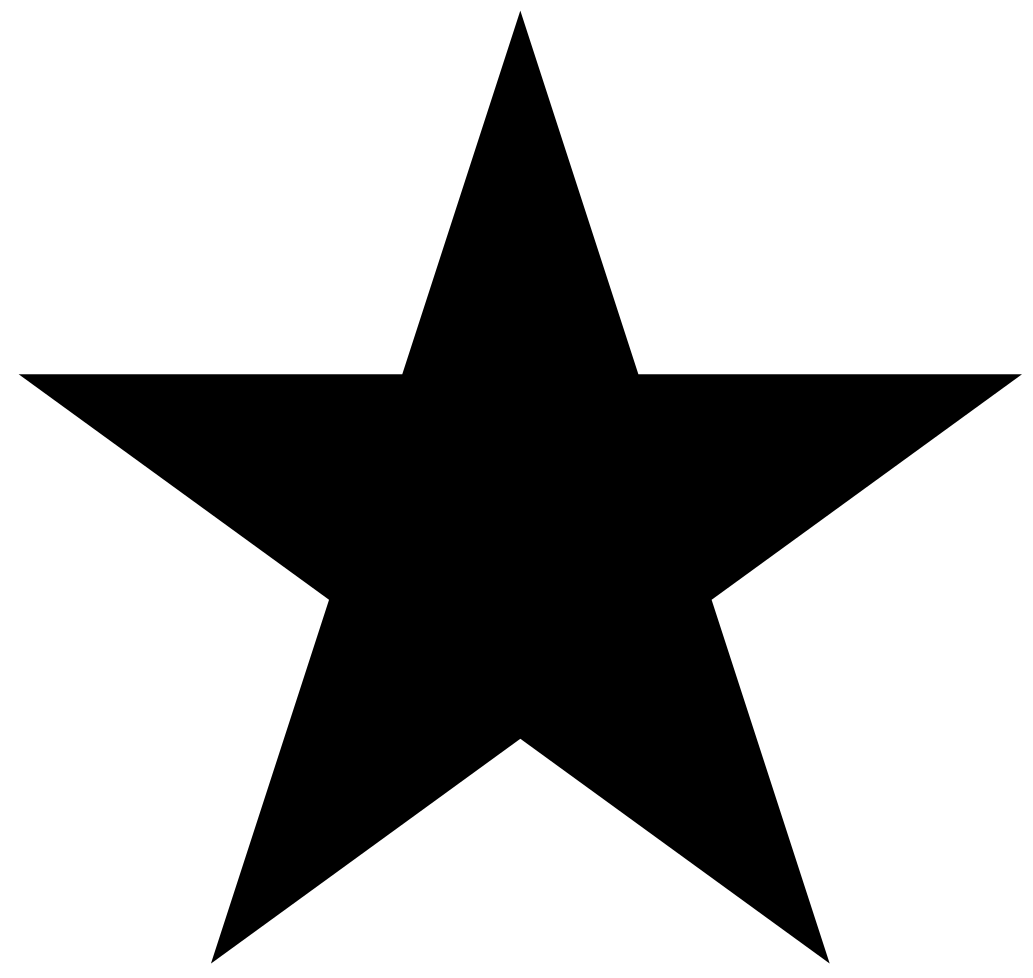 | 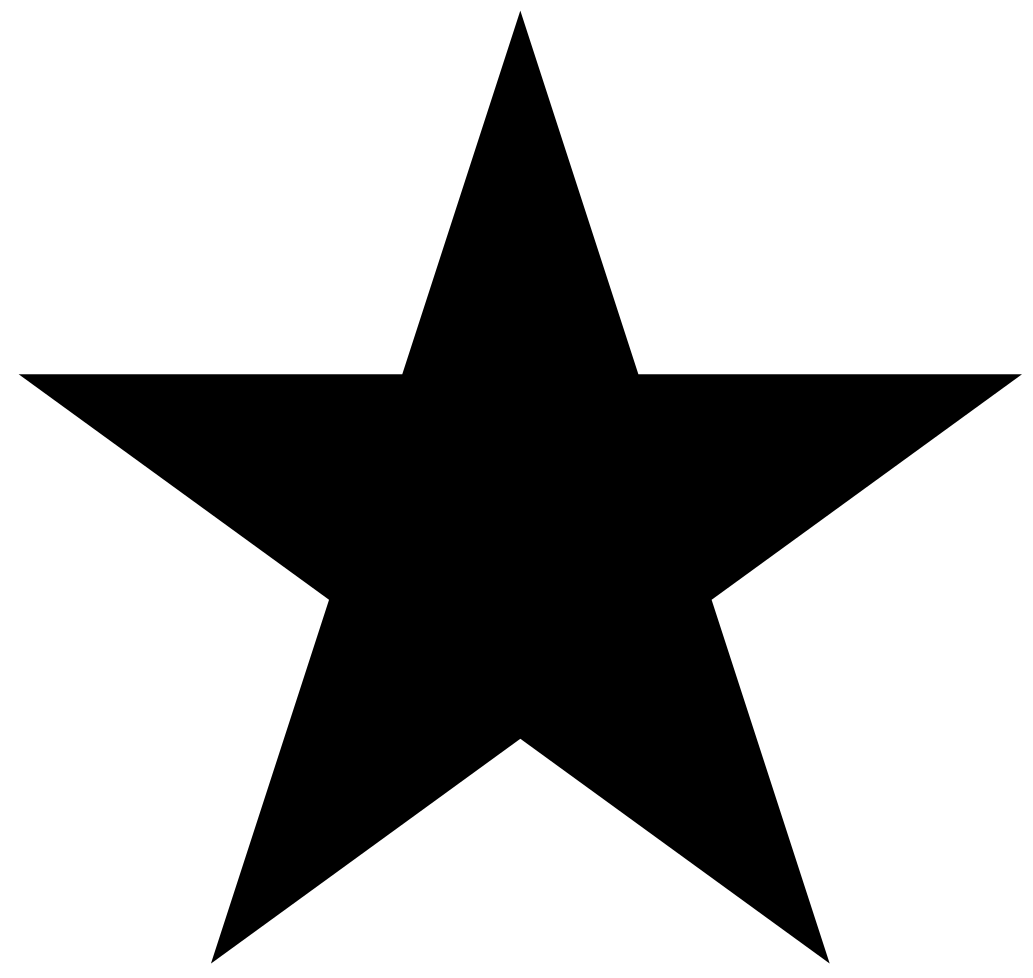 **4** |
| Asare (2014) 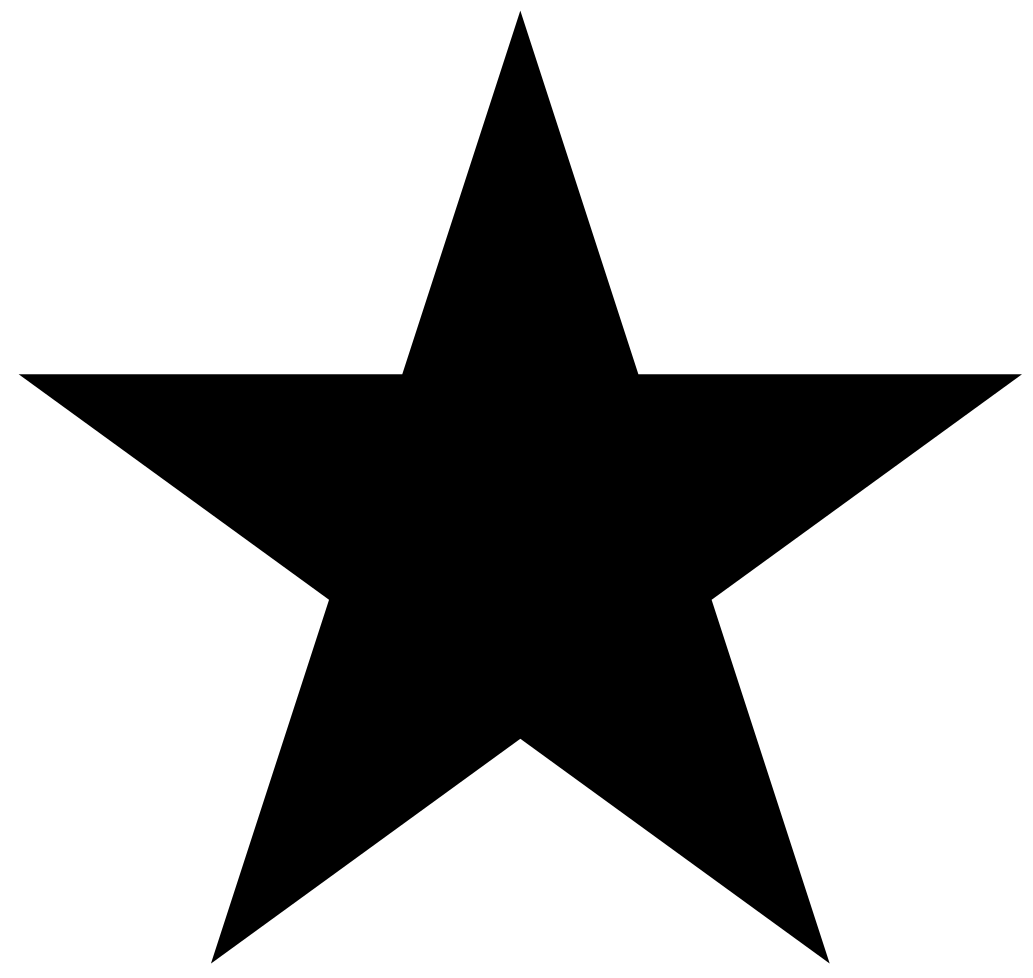 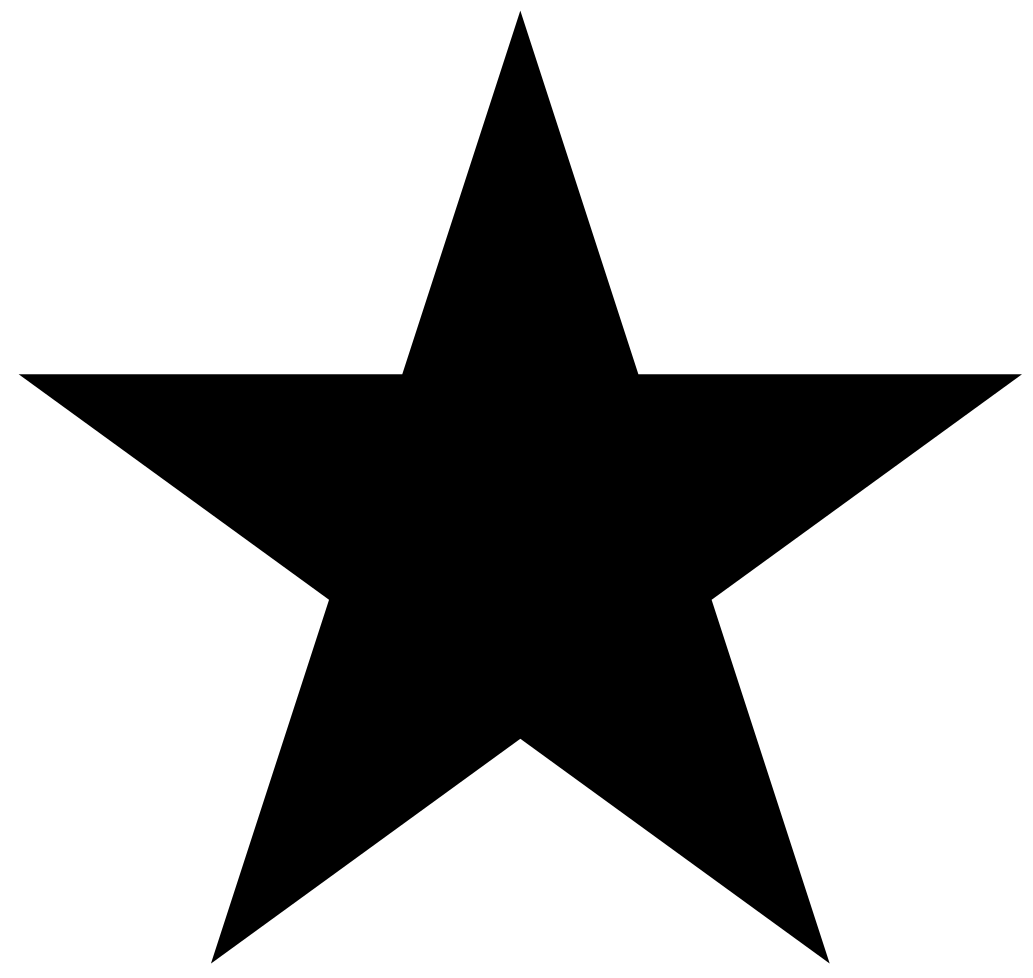 | 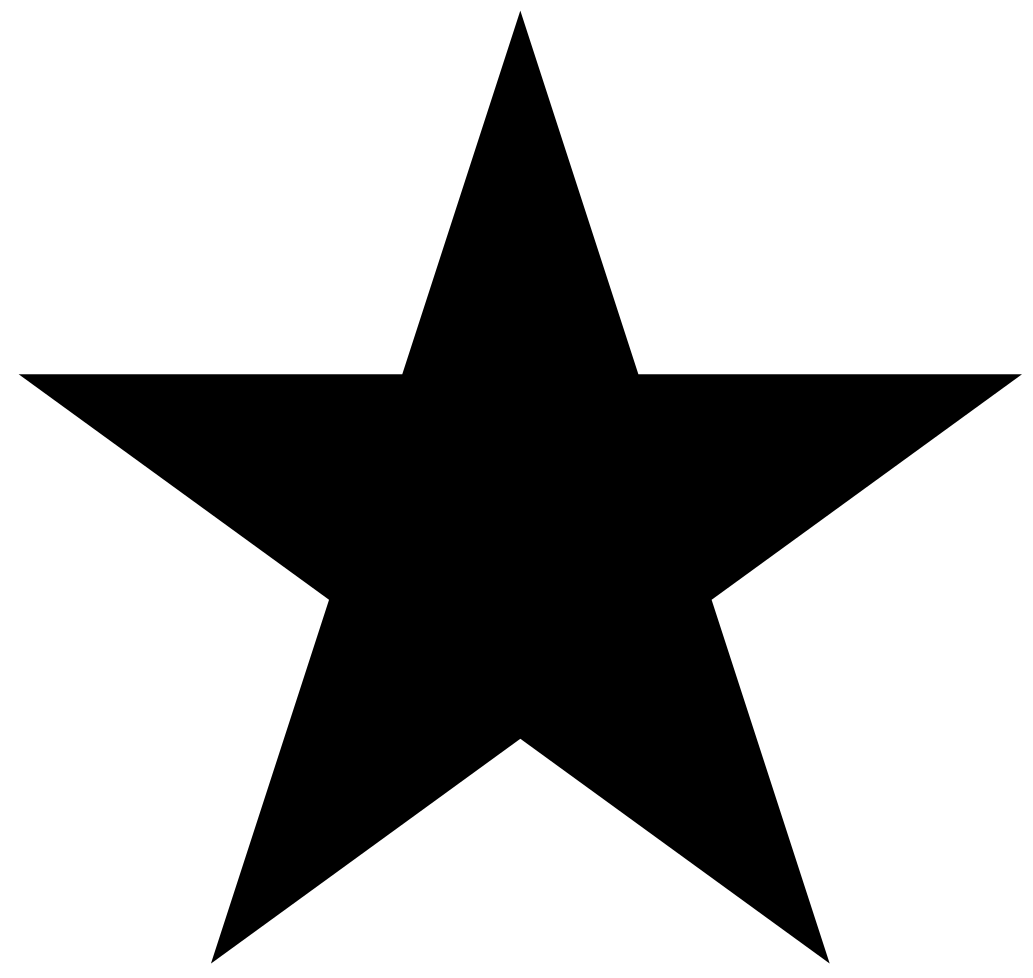 **3** |
| Haroon et al. (2014) 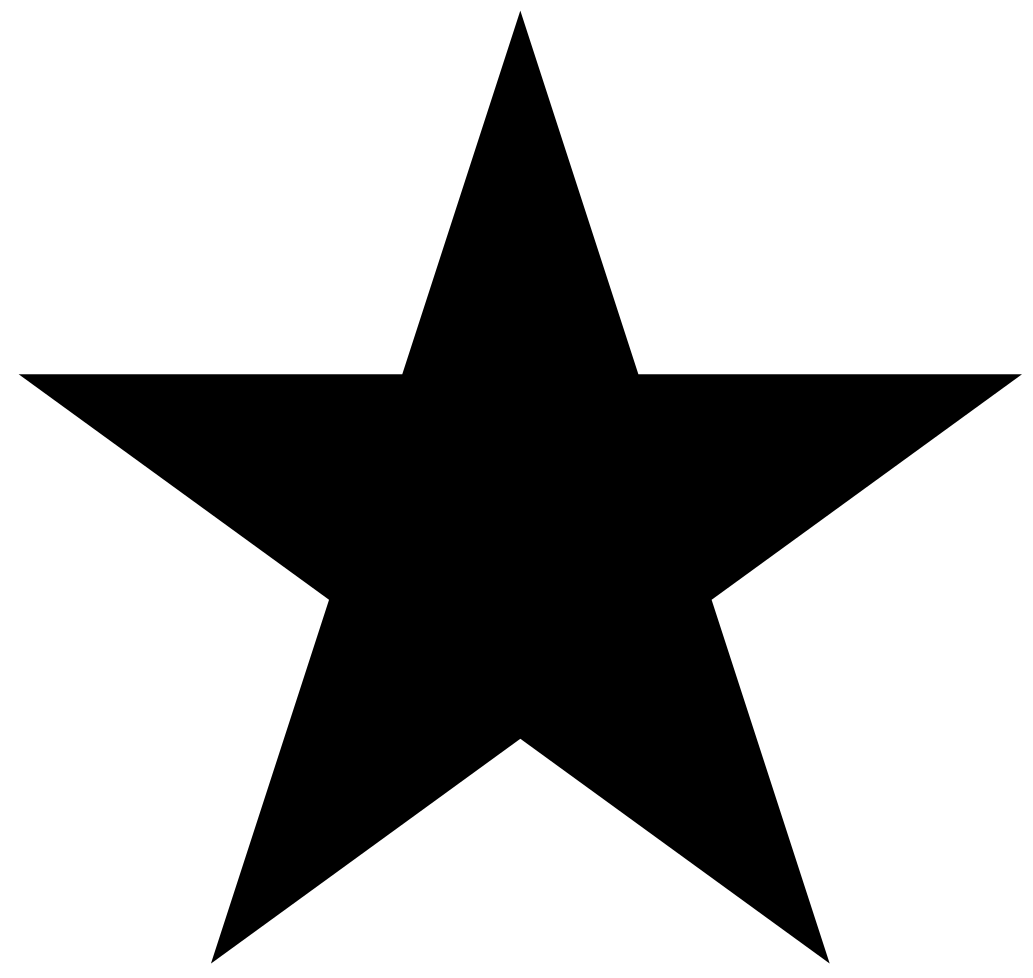 | 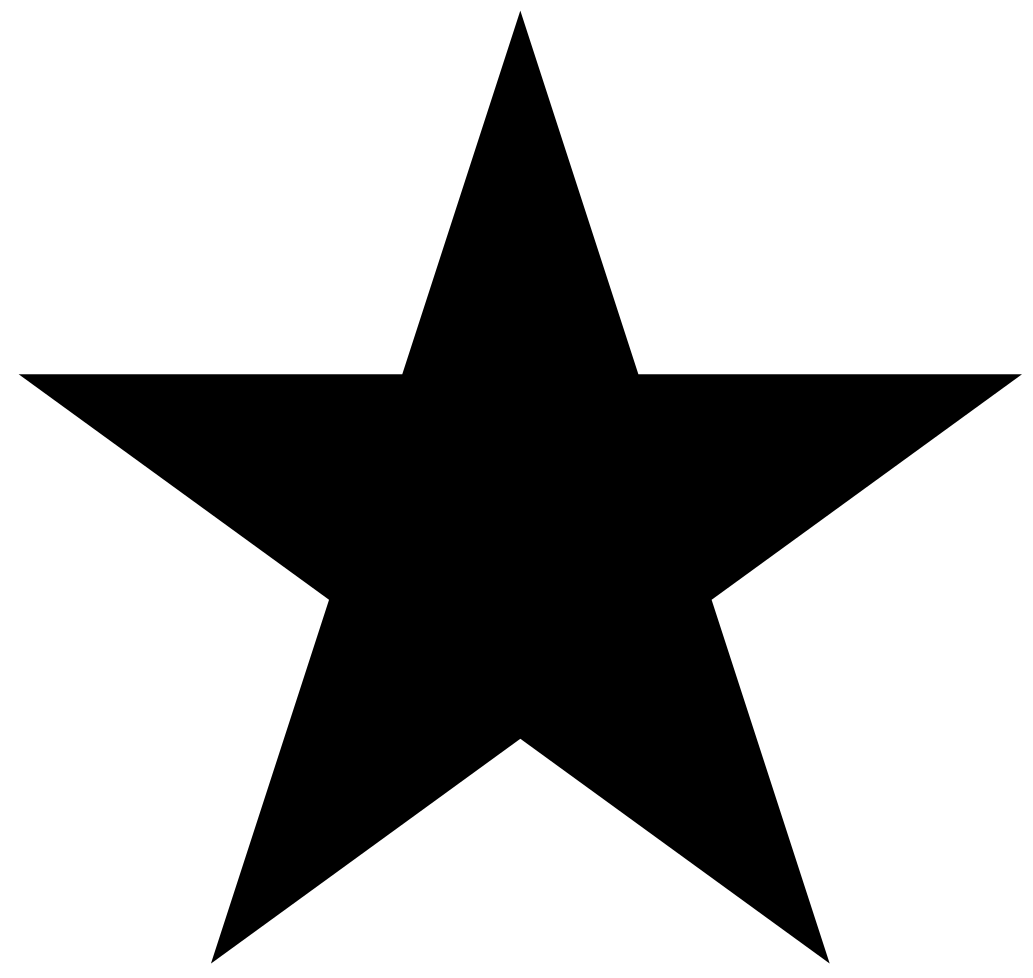 **2** |
| Odutayo et al. (2015) 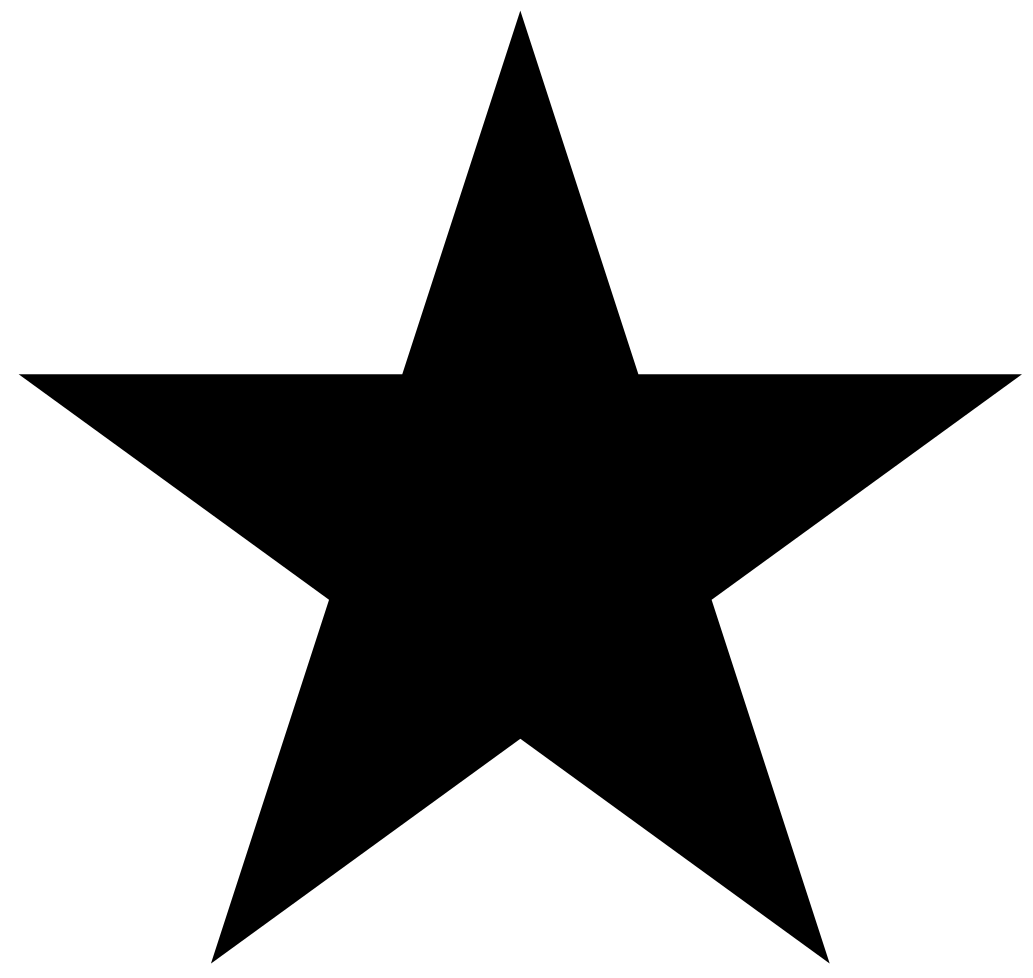 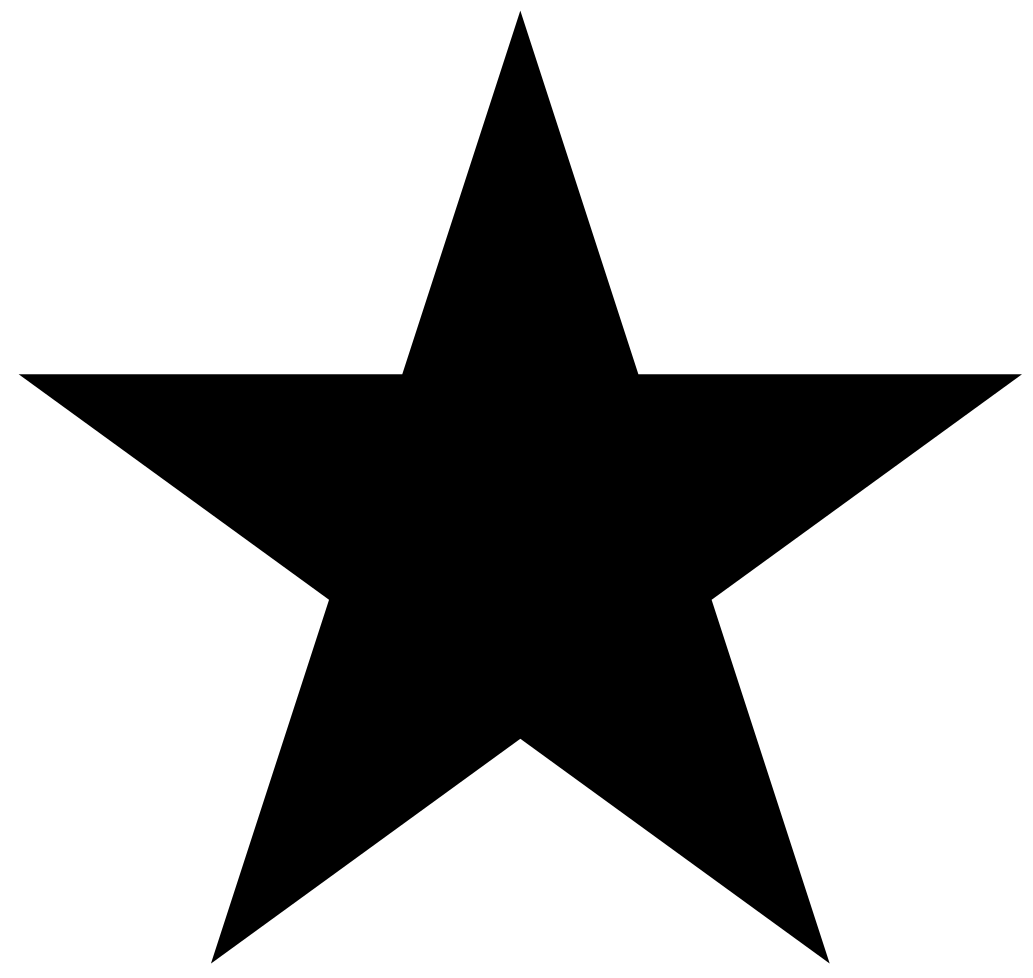 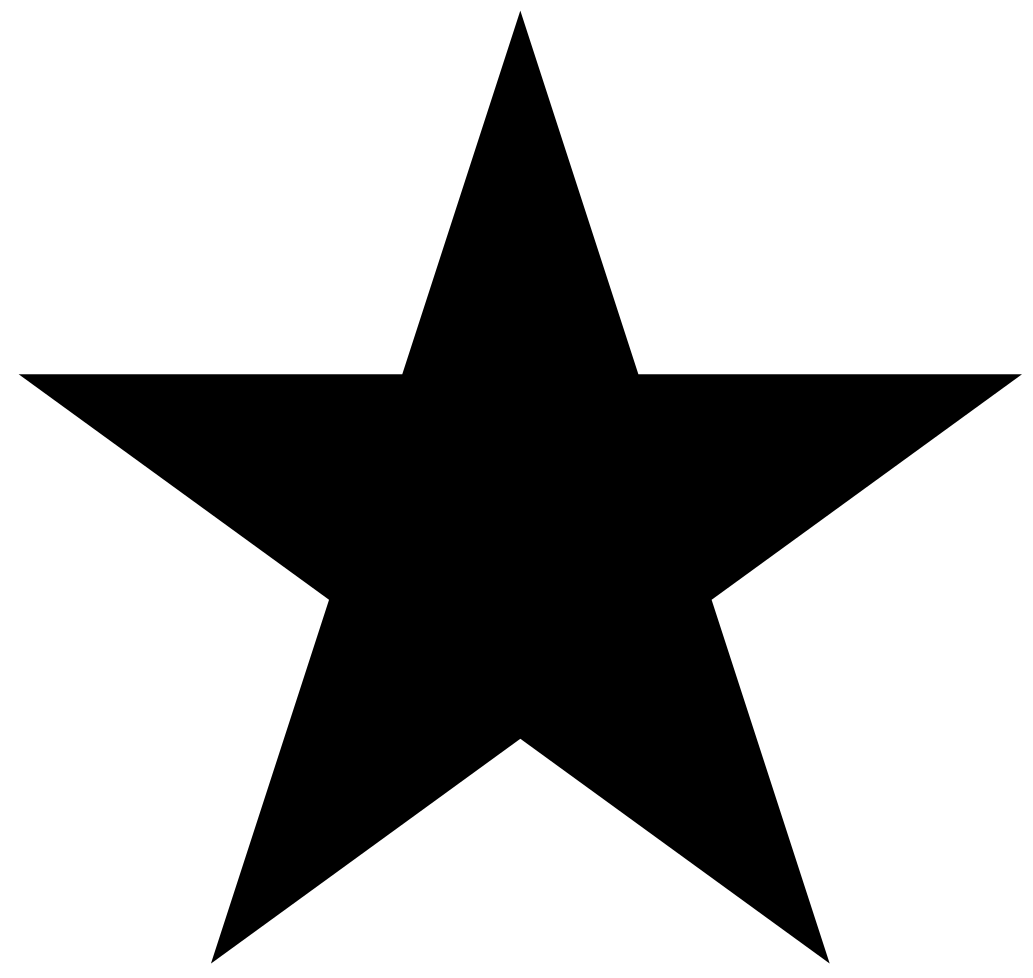 | 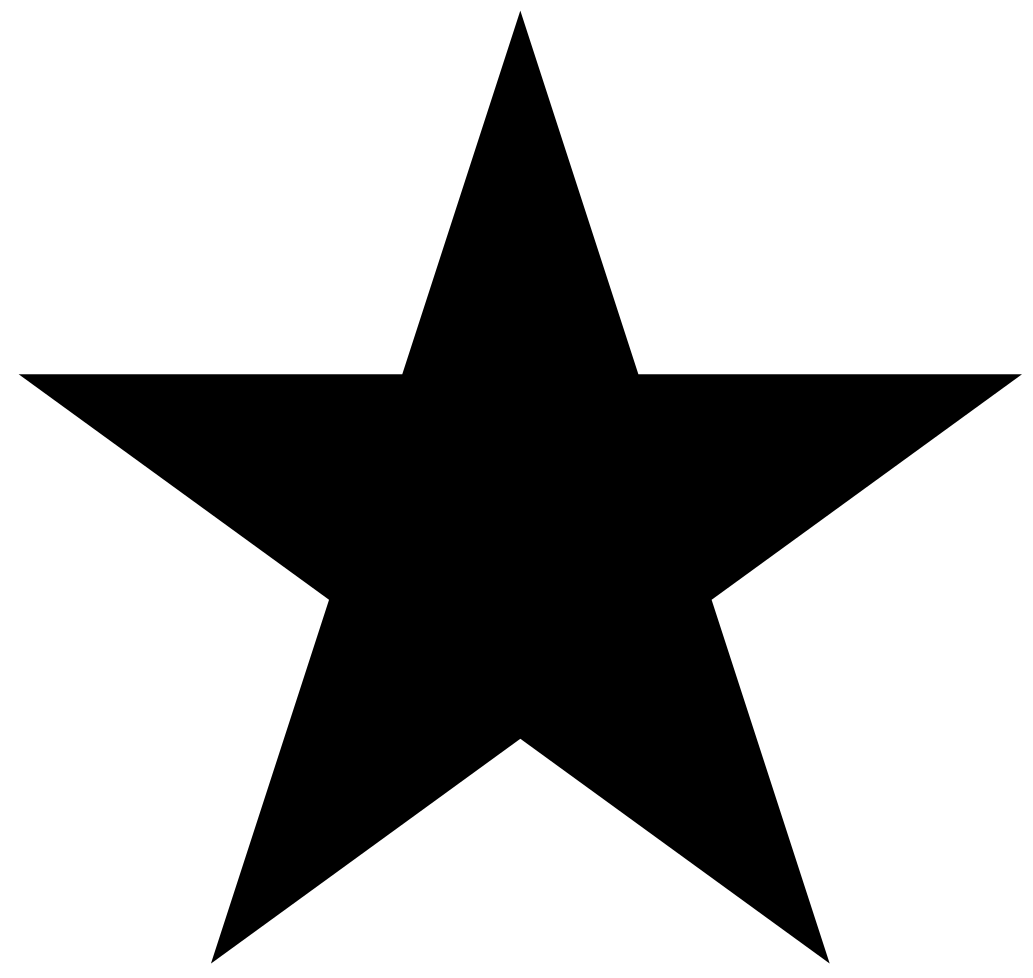 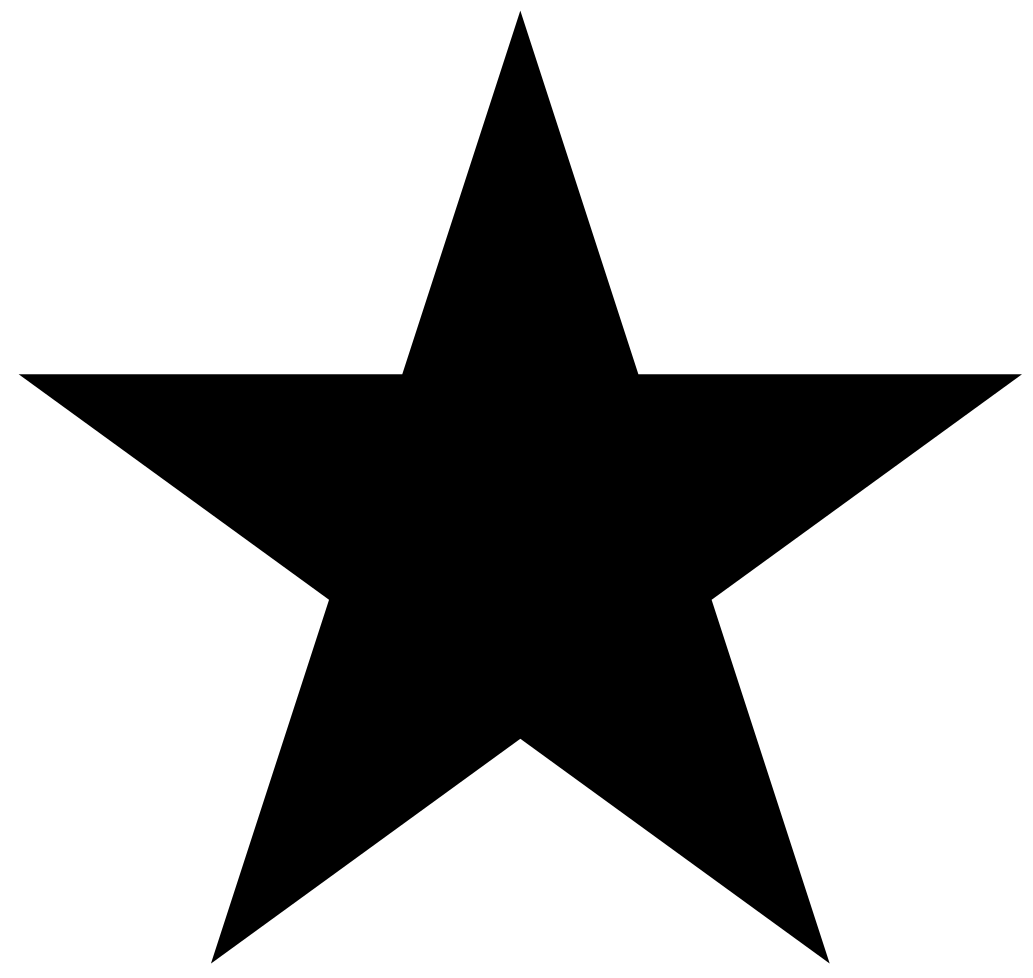 **6** |


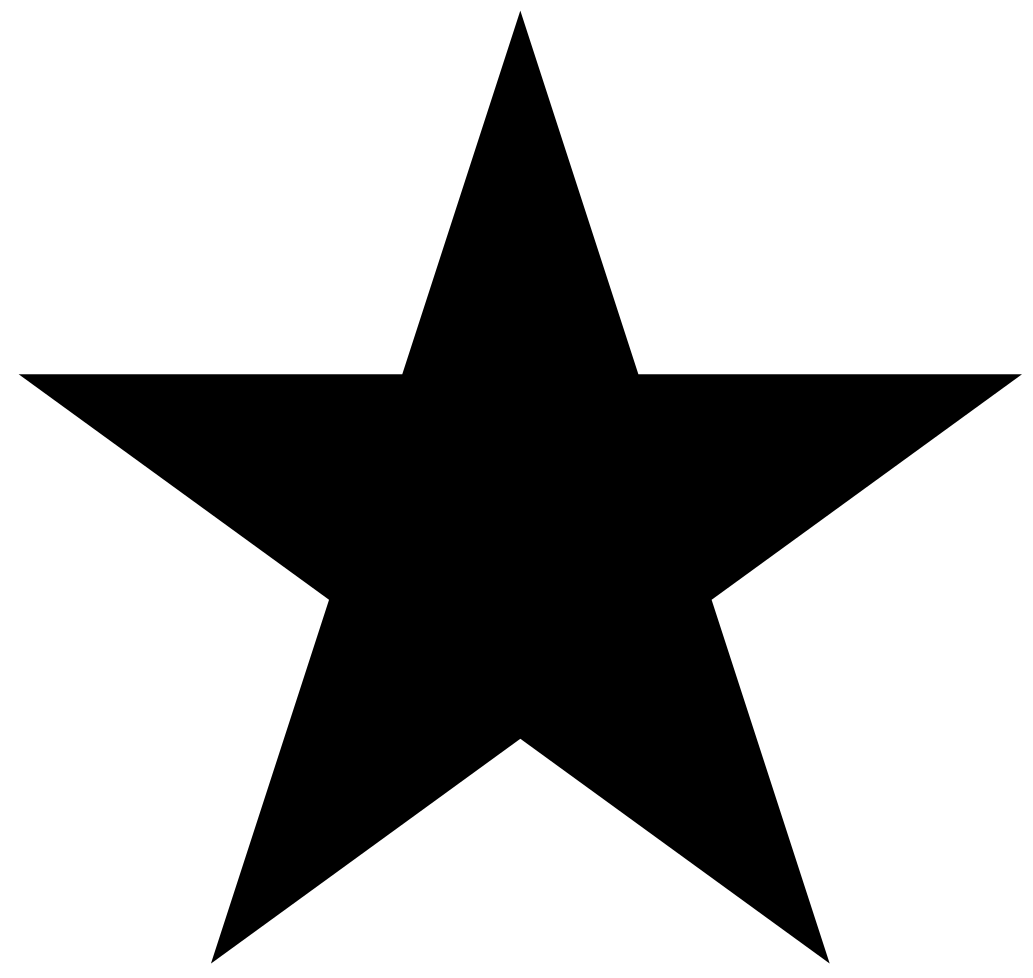
Note: (1) Is the case definition adequate; (2) Representativeness of the cases; (3) Selection of controls; (4) definition of controls; (5) comparability of cases and controls on the basis of the design of analysis; (6) ascertainment of exposure; (7) same method of ascertainment of cases and controls; (8) non-response rate.
