# Supplementary material for: The effect of non‐oral hormonal contraceptives on hypertension and blood pressure: A systematic review and meta‐analysis
Source: Physiol Rep. 2022 May 4;10(9):e15267. doi: 10.14814/phy2.15267 (PMC9069167; doi:10.14814/phy2.15267)
Supplement: Supplementary file 6 — Table S4 [file PHY2-10-e15267-s003.docx]

| Table 4: Cochrane Risk of Bias Tool for Randomized Controlled Trials  **Selection Performance Detection Attrition Reporting Other Bias Bias Bias Bias Bias Bias** | | | | | | | | | |
| --- | --- | --- | --- | --- | --- | --- | --- | --- | --- |
| **First Author (Year)** | **1** |  | **2** |  | **3** | **4** | **5** | **6** | **7** |
| Battaglia et al. (2010) |  | - |  | **+** | **-** | **-** | **-** | **-** | **?** |

(2011)

| Zueff et al. (2010) **?** | **-** | **?** | **+** | **+** | **+** | **?** |
| --- | --- | --- | --- | --- | --- | --- |
| Mohamed et al. **?** | **?** | **+** | **+** | **-** | **-** | **?** |

Note: (1) random sequence generation; (2) allocation concealment; (3) blinding of participants and personnel; (4) blinding of outcome assessment; (5) incomplete outcome data; (6) reporting bias sue to selective outcome reporting; (7) Other Bias. Reviewer assessment: + high bias; - low bias; ? unclear bias.
